# USP13 stabilizes NLRP3 to facilitate inflammasome activation by preventing TRIM31-mediated NLRP3 ubiquitination and degradation

**DOI:** 10.1126/sciadv.adx3827

**Published:** 2025-09-26

**Authors:** Ya-Ting Li, Ke-Ying Li, Shou-Song Tao, Ting Wang, Ying Lu, Hui Chen, Yi-Qun Zhan, Ke Zhao, Shen-Si Xiang, Jing-Jing Li, Hui-Ying Gao, Miao Yu, Chang-Yan Li, Lin Wang, Xiao-Ming Yang, Guang-Ming Ren, Rong-Hua Yin

**Affiliations:** ^1^ Faculty of Chemistry and Life Sciences, Beijing University of Technology, Beijing 100124, China.; ^2^ State Key Laboratory of Medical Proteomics, Beijing Proteome Research Center, National Center for Protein Sciences (Beijing), Beijing Institute of Radiation Medicine, Beijing 100850, China.; ^3^ Department of Immunology, College of Basic Medicine, Qingdao University, Qingdao 266071, Shandong Province, China.; ^4^ Department of Blood Transfusion, Sichuan Clinical Research Center for Cancer, Sichuan Cancer Hospital & Institute, Sichuan Cancer Center, University of Electronic Science and Technology of China, Chengdu 610000, Sichuan Province, China.; ^5^ Department of Pharmaceutical Engineering, School of Chemical Engineering and Technology, Tianjin University, Tianjin 300072, China.

## Abstract

NOD-, LRR-, and pyrin domain–containing protein 3 (NLRP3) has a fundamental role in host defense and is involved in diverse inflammatory diseases. NLRP3 protein expression is tightly controlled by the ubiquitin system. In particular, NLRP3 protein degradation has been extensively studied. In contrast, the mechanisms to stabilize NLRP3 protein are much less known. Here, we demonstrated the critical role of ubiquitin-specific protease 13 (USP13) in regulating NLRP3 protein stability and inflammasome activation independently of its deubiquitinating enzyme activity. USP13 competes with E3 ubiquitin ligase TRIM31 to interact with NLRP3 and prevents TRIM31-mediated NLRP3 ubiquitination at K192 and K496 sites, thereby inhibiting proteasomal degradation of NLRP3. USP13 deficiency reduces NLRP3 protein expression in both human and mouse macrophages, which consequently inhibits NLRP3 inflammasome assembly and activation. Accordingly, deficiency of USP13 attenuates monosodium urate crystal–induced mouse peritonitis. Overall, our findings reveal a previously unrecognized regulatory mechanism of NLRP3 stability by USP13 and provide a potential therapeutic target for NLRP3-driven diseases.

## Teaser

USP13 promotes NLRP3 inflammasome activation by enhancing NLRP3 protein stability.

## INTRODUCTION

NOD-, LRR (leucine-rich repeat)-, and pyrin domain (PYD)–containing protein 3 (NLRP3) is a cytosolic pattern recognition receptor that senses numerous pathogen-associated molecular patterns derived from exogenous invaders and damage-associated molecular patterns released from damaged host cells (*1*). Upon activation, NLRP3 undergoes oligomerization and then recruits apoptosis-associated speck-like protein containing a CARD (ASC) and pro–caspase-1 to form the NLRP3 inflammasome. The assembly of the NLRP3 inflammasome induces caspase-1 self-cleavage and activation, resulting in the maturation and release of the pro-inflammatory cytokines, such as interleukin-1β (IL-1β) and IL-18, and the induction of gasdermin D (GSDMD)–mediated pyroptosis (*2*). Canonical activation of the NLRP3 inflammasome requires two independent signals, namely priming and activation. The priming signals, such as lipopolysaccharide (LPS), increase the transcription of NLRP3 and pro–IL-1β and induce posttranslational modifications on NLRP3 to inhibit NLRP3 degradation and license NLRP3 to respond to further stimuli. After priming, NLRP3 adopts a cage-like formation. The activation signals, such as nigericin, adenosine 5′-triphosphate (ATP), and monosodium urate (MSU) crystals, trigger NLRP3 to transition from its inactive cage structure to the active form and initiate the inflammasome assembly together with ASC and pro–caspase-1. Multiple proposed mechanisms are involved in the activation of NLRP3 inflammasome assembly including ion homeostasis, lysosomal rupture, mitochondrial damage, and Golgi dispersion (*2*–*4*). Activation of the NLRP3 inflammasome is critical for host immune defenses against pathogenic infections; however, aberrant activation of the NLRP3 inflammasome contributes to the progression of various inflammatory diseases, such as cryopyrin-associated periodic syndromes, gout, arthritis, atherosclerosis, and Alzheimer’s disease (*5*). Therefore, the activation of the NLRP3 inflammasome is strictly controlled by numerous pathways and mechanisms to maintain inflammation homeostasis.

Multiple posttranslational modifications of NLRP3 have been identified in regulating the activation of the NLRP3 inflammasome, including phosphorylation, ubiquitination, sumoylation, acetylation, and S-nitrosylation (*6*). Among them, ubiquitination induces NLRP3 degradation to maintain an extremely low level of NLRP3 protein content in resting cells (*5*, *7*). At the resting state, NLRP3 undergoes ubiquitination with K48-, K63-, K27-, and K6-linked polyubiquitin chains, which promotes NLRP3 degradation through proteasomal or autophagic degradation (*8*–*11*). Multiple E3 ubiquitin ligases are involved in NLRP3 ubiquitination and negatively regulate NLRP3 inflammasome activation. Membrane-associated ring-CH-type finger 7 (MARCH7) mediates K48-linked ubiquitination and autophagic degradation of NLRP3 (*12*). Tripartite motif containing 31 (TRIM31), F-box and leucine-rich repeat protein 2 (FBXL2), and Cbl-b promote K48-linked ubiquitination and proteasomal degradation of NLRP3 (*13*–*15*). β-TrCP1 induces K27-linked ubiquitination and proteasomal degradation of NLRP3 (*10*). Therefore, deubiquitination of NLRP3 is required for its stability as well as a rapid and optimal activation of the NLRP3 inflammasome. The deubiquitinases (DUBs) ubiquitin-specific protease 1 (USP1) and OTU deubiquitinase 6A (OTUD6A) cleave K48-linked polyubiquitin chains from NLRP3 and prevent its proteasomal degradation (*16*, *17*). USP19 induces K48-linked deubiquitination of NLRP3 and inhibits its autophagic degradation (*11*). However, in contrast to the extensive studies on NLRP3 ubiquitination and degradation, the underlying regulatory mechanisms of NLRP3 deubiquitination and stability are relatively less known.

USP13 is a crucial member of the USP subfamily of DUBs that participates in various cellular functions, including cell cycle, DNA damage response, autophagy, endoplasmic reticulum–associated degradation, and signaling pathways (*18*, *19*). The impact on these processes implicates USP13 in multiple pathogenic conditions, contributing to infection, inflammation, fibrosis, neurodegenerative diseases, and tumorigenesis (*18*). USP13 has DUB activity against K48-, K63-, K27-, and K33-linked polyubiquitin chains and regulates the stability, activity, or function of substrate proteins. For example, USP13 interacts with and stabilizes ATG5 by removing the K48-linked polyubiquitin chains from ATG5, which induces prosurvival autophagy in gastrointestinal stromal tumors (*20*). USP13 deubiquitinates the K63-linked ubiquitin chain of RAP80 (receptor-associated protein 80), resulting in the recruitment of the RAP80-BRCA1 (breast cancer type 1 susceptibility protein) complex to double-strand breaks to facilitate DNA damage response (*21*). USP13 deconjugates K27- and K33-linked polyubiquitin chains from STING (stimulator of interferon genes) and prevents the recruitment of TBK1 (TANK binding kinase 1) to STING, negatively regulating cellular antiviral responses (*22*). Recent studies showed that USP13 plays important roles in inflammatory responses by targeting multiple substrates, such as Sigirr [single immunoglobulin and toll-interleukin-1 receptor (TIR) domain], IRAK4 (interleukin-1 receptor–associated kinase 4), PTEN (phosphatase and tensin homolog), Irhom2 (inactive rhomboid protein 2), and TAK1 (transforming growth factor–β–activated kinase 1) (*23*–*27*). In particular, several in vitro studies showed that USP13 was involved in NLRP3 inflammasome activation. USP13-induced NLRP3 deubiquitination is essential for paxillin-mediated activation of the P2X7 receptor and NLRP3 inflammasome upon ATP treatment (*28*). Besides, USP13 is also linked to NLRP3 protein expression (*29*). However, whether USP13 regulates NLRP3 stability and activation in vivo and the underlying mechanisms are still unclear.

In this study, we identified USP13 as an important stabilizer of NLRP3. USP13 binds to the PYD of NLRP3, which prevents the interaction of NLRP3 with its E3 ubiquitin ligase TRIM31. In turn, USP13 inhibits TRIM31-mediated K48-linked polyubiquitination of NLRP3 at K192 and K496 residues, thereby blocking NLRP3 proteasomal degradation. USP13-deficient macrophages show reduced NLRP3 protein levels and NLRP3 inflammasome activation. USP13 deletion mice are resistant to MSU-induced peritonitis. Overall, USP13 positively regulates NLRP3 inflammasome activation by enhancing NLRP3 protein stability.

## RESULTS

### Identification of USP13 as an NLRP3-binding DUB

**Fig. 1.**
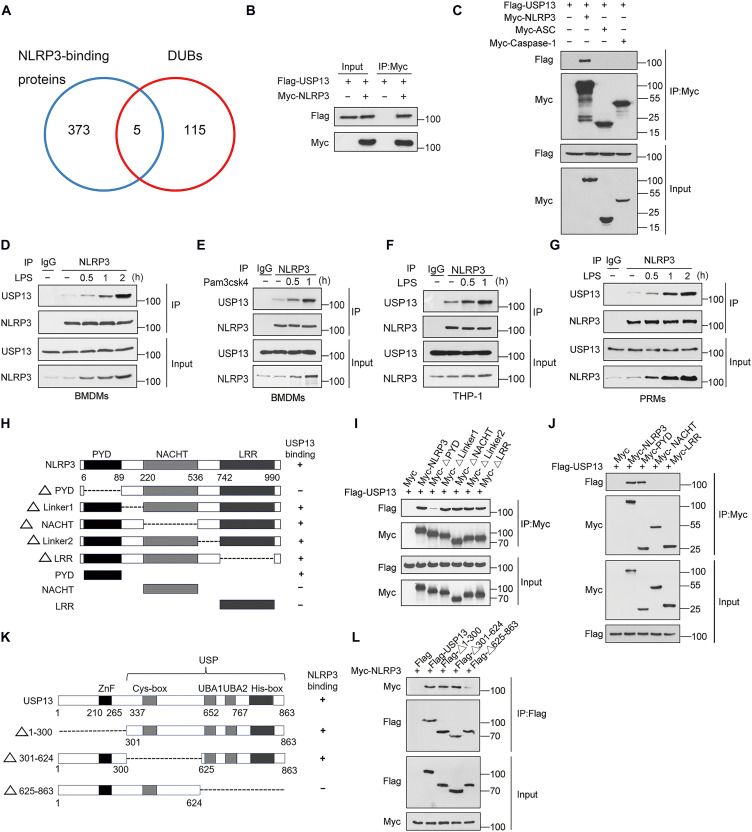
Identification of USP13 as an NLRP3-binding DUB. (**A**) Screening of NLRP3-interacting DUBs in Flag-tagged human NLRP3–overexpressed THP-1 cells by IP-MS. (**B** and **C**) HEK293T cells were transfected with the indicated plasmids. Immunoblot analysis of Myc- and Flag-tagged proteins in cell lysates immunoprecipitated with anti–c-Myc beads. (**D** and **E**) Murine BMDMs were treated with LPS (100 ng/ml) (D) or Pam3CSK4 (200 ng/ml) (E) for indicated times. Immunoblot analysis of USP13 and NLRP3 in cell lysates immunoprecipitated with a control IgG or anti-NLRP3 antibody. h, hours. (**F** and **G**) THP-1 cells and murine PRMs were treated with LPS for indicated times. Immunoblot analysis of USP13 and NLRP3 in cell lysates immunoprecipitated with a control IgG or anti-NLRP3 antibody. (**H**) Schematic representation of human NLRP3 and its truncation mutants. (**I** and **J**) HEK293T cells were transfected with various combinations (above lanes) of plasmids encoding Flag-tagged human USP13 and Myc-tagged human NLRP3 or its truncation mutants. Immunoblot analysis of Myc- and Flag-tagged proteins in cell lysates immunoprecipitated with anti–c-Myc beads. (**K**) Schematic representation of USP13 and its truncation mutants. (**L**) HEK293T cells were transfected with various combinations (above lanes) of plasmids encoding Myc-tagged human NLRP3 and Flag-tagged human USP13 or its truncation mutants. Immunoblot analysis of Myc- and Flag-tagged proteins in cell lysates immunoprecipitated with anti-Flag M2 beads. Data are representative of three independent experiments [(B) to (G), (I), (J), and (L)].

To identify the DUBs that stabilize NLRP3 protein, we screened NLRP3-binding proteins using an immunoprecipitation-mass spec-trometry (IP-MS) assay. Flag-tagged human NLRP3 was overexpressed in THP-1 cells by a lentivirus. The proteins coimmunoprecipitated with Flag-tagged NLRP3 were subjected to MS analysis. A total of 378 potential NLRP3-interacting proteins was obtained, including five DUBs, USP13, USP7, EIF3H (eukaryotic translation initiation factor 3 subunit H), USP9X, and BRCC3 (BRCA1/BRCA2–containing complex subunit 3) (Fig. 1A and table S1). Previous work by us and others has confirmed that BRCC3 catalyzes NLRP3 K63-linked deubiquitination and promotes NLRP3 inflammasome assembly (*8*, *30*). USP9X cleaves NLRP3 K48-linked polyubiquitination and stabilizes NLRP3 protein (*31*). USP7 increases NLRP3 inflammasome activation by reducing NLRP3 ubiquitination but has no effect on NLRP3 stability (*32*). EIF3H typically functions as a subunit of eIF3 complex involved in the translation initiation regulation (*33*). We noticed that USP13 has been shown to be an NLRP3-binding protein and positively regulates NLRP3 inflammasome activation in in vitro–cultured cells (*8*, *28*). However, the in vivo role of USP13 in NLRP3 ubiquitination and activation is still unknown. In particular, there is a faint hint indicating that USP13 is involved in the regulation of NLRP3 protein stability (*29*). Thus, we next focus on whether and how USP13 stabilizes NLRP3.

We first confirmed the interaction between NLRP3 and USP13 in human embryonic kidney (HEK) 293T cells overexpressed with Myc-tagged human NLRP3 and Flag-tagged human USP13 by coimmunoprecipitation assay (Fig. 1B). USP13 only binds to NLRP3 but not to ASC or pro–caspase-1, the other two subunits of the NLRP3 inflammasome, when coexpressed together in HEK293T cells (Fig. 1C). We further detected the endogenous association between NLRP3 and USP13 in murine bone marrow–derived macrophages (BMDMs). Our results showed that USP13 had a weak interaction with NLRP3 in resting BMDMs. Priming signals such as LPS and Pam3CSK4 could potently increase their interaction (Fig. 1, D and E). Similar results were also observed in THP-1 cells and murine peritoneal resident macrophages (PRMs) (Fig. 1, F and G). NLRP3 contains three canonical domains: a C-terminal LRR, a central nucleotide binding and oligomerization domain termed NACHT, and an N-terminal PYD (*34*). To search for the domains of NLRP3 that are responsible for the interaction with USP13, a series of human NLRP3-truncated mutants was constructed (Fig. 1H). By coimmunoprecipitation assays, we found that the truncated NLRP3 lacking the PYD (amino acid residues 6 to 98), but not the other domains, sharply reduced the ability to interact with USP13 (Fig. 1I). On the contrary, only the truncated NLRP3 containing the PYD was able to bind with USP13 (Fig. 1J). These data clearly confirmed that the PYD of NLRP3 is required for USP13 binding. Three truncated mutants of human USP13 were also constructed (Fig. 1K) (*19*). The results showed that the truncated USP13 lacking the N-terminal region (amino acid residues 625 to 863) almost lost its ability to interact with NLRP3, indicating that NLRP3 binds to the N-terminal region of USP13 (Fig. 1L).

### USP13 increases NLRP3 protein stability

**Fig. 2.**
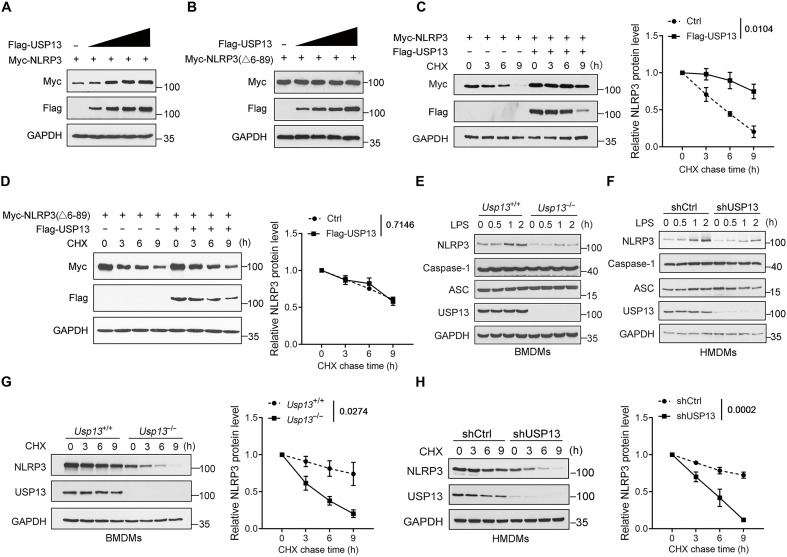
USP13 increases NLRP3 protein stability. (**A** and **B**) Immunoblot analysis of NLRP3 (A) or NLRP3-△6-89 (B) protein levels in HEK293T cells transfected with increasing amounts of Flag-tagged human USP13. (**C** and **D**) HEK293T cells transfected with Myc-tagged human NLRP3 (C) or Myc-tagged human NLRP3-△6-89 (D) together with Flag-tagged human USP13 or control vector were treated with CHX (50 μg/ml) for indicated times and then analyzed by Western blotting. The representative Western blot and quantification of relative protein levels are shown. (**E**) *Usp13*^+/+^ and *Usp13*^−/−^ BMDMs were treated with LPS for indicated times. Immunoblot analysis of NLRP3, caspase-1, and ASC protein levels in cell lysates. (**F**) HMDMs transduced with lentiviruses expressing shCtrl or shUSP13 were treated with LPS for indicated times. Immunoblot analysis of NLRP3, caspase-1, and ASC protein levels in cell lysates. (**G**) *Usp13*^+/+^ and *Usp13*^−/−^ BMDMs were treated with CHX for indicated times and then analyzed by Western blotting. (**H**) HMDMs transduced with lentiviruses expressing shCtrl or shUSP13 were treated with CHX for indicated times and then analyzed by Western blotting. Data are representative of three independent experiments [(A) to (H)] or presented as the means ± SEM from three independent experiments [(C), (D), (G), and (H)]. Statistical significance was assessed by two-way ANOVA with Bonferroni’s multiple comparisons test [(C), (D), (G), and (H)].

A previous study showed that the overexpression of USP13 increased the NLRP3 expression level in the PC12 neuronal cell line (*29*). As USP13 typically functions as a K48-linked DUB to control protein degradation, we thus examined whether USP13 regulates NLRP3 protein stability. Myc-tagged human NLRP3, together with an increasing amount of Flag-tagged human USP13, was transfected into HEK293T cells, and Western blot analysis showed that USP13 dose-dependently increased the NLRP3 protein level (Fig. 2A). In contrast, USP13 overexpression could not promote the protein level of the NLRP3-△6-89 mutant lacking the USP13-binding domain (Fig. 2B). Furthermore, we demonstrated that the overexpression of USP13 significantly extended the half-life of wild-type (WT) NLRP3 protein, but not the NLRP3-△6-89 mutant, in HEK239T cells by the cycloheximide (CHX) chase experiment (Fig. 2, C and D). Therefore, our results demonstrated that USP13 enhances the protein stability of NLRP3 by binding to its PYD.

We also tested the effect of USP13 deficiency on NLRP3 protein stability in mouse and human macrophages. The results showed that LPS priming time-dependently increased the NLRP3 protein level in WT murine BMDMs, while this process was potently inhibited in USP13 knockout cells (Fig. 2E). Besides, USP13 deficiency did not affect ASC and caspase-1 protein levels (Fig. 2E). Similar results were also observed in human monocyte-derived macrophages (HMDMs) with USP13 knockdown by a lentivirus (Fig. 2F). Moreover, the half-life of NLRP3 protein was markedly reduced in *Usp13*^−/−^ BMDMs, as well as in USP13 knockdown HMDMs, compared to control cells after CHX treatment (Fig. 2, G and H). It is worth noting that NLRP3 protein levels in resting USP13-deficient cells were lower than in control cells (Fig. 2, E to H), indicating that USP13 is required for maintaining the basal cellular level of NLRP3 protein. USP13 has been reported to inhibit LPS/Toll-like receptor 4 (TLR4)–induced pro-inflammatory responses (*24*), which indicates that USP13 may affect the transcription of NLRP3. We thus examined the effect of USP13 on the mRNA level of NLRP3 after LPS priming and found that USP13 deficiency had no effect on NLRP3 transcription in BMDMs after LPS treatment (fig. S1A). Consistently, USP13 deficiency also did not affect the transcription of IL-1β and the release of tumor necrosis factor–α (TNF-α) and IL-6 in BMDMs induced by LPS (fig. S1, A and B). These data excluded the possibility that USP13 deficiency reduced the NLRP3 protein level by decreasing NLRP3 transcription. Together, our results confirmed that USP13 is essential for maintaining NLRP3 protein stability.

### USP13 diminishes K48-linked ubiquitination of NLRP3

**Fig. 3.**
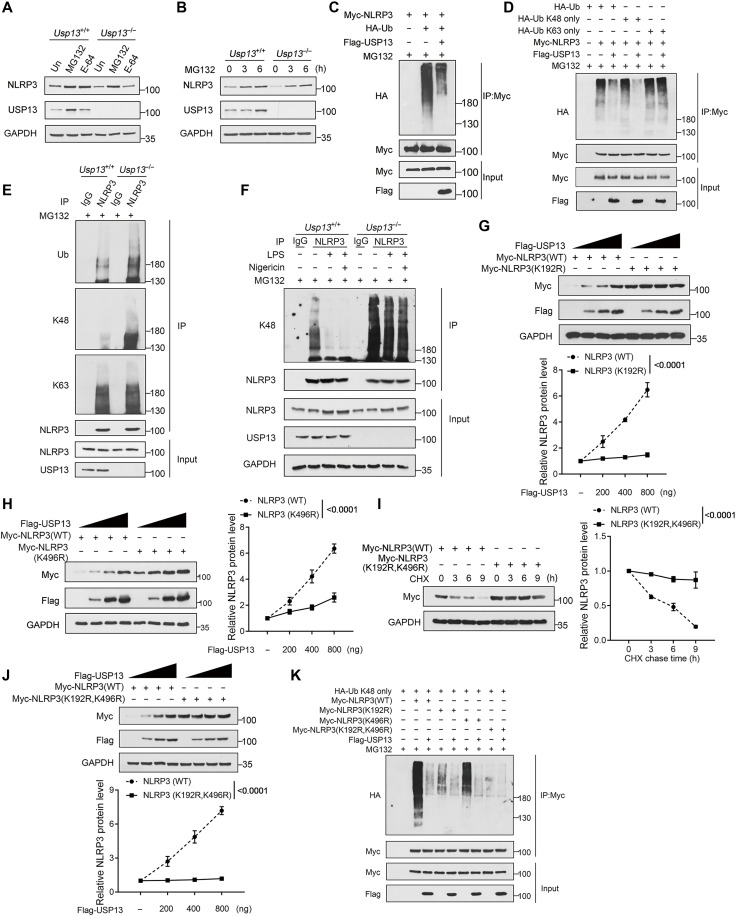
USP13 diminishes the K48-linked ubiquitination of NLRP3. (**A**) *Usp13*^+/+^ and *Usp13*^−/−^ BMDMs were treated with MG132 (20 μM) for 6 hours or E-64 (50 μM) for 12 hours. Immunoblot analysis of NLRP3 protein levels in cell lysates. (**B**) *Usp13*^+/+^ and *Usp13*^−/−^ BMDMs were treated with MG132 for indicated times. Immunoblot analysis of NLRP3 protein levels in cell lysates. (**C** and **D**) HEK293T cells transfected with various combinations of plasmids as indicated above the lanes were treated with MG132 for 6 hours. Immunoblot analysis of NLRP3 ubiquitination in cell lysates immunoprecipitated with anti–c-Myc beads. (**E** and **F**) *Usp13*^+/+^ and *Usp13*^−/−^ BMDMs treated with MG132 for 6 hours were left untreated or treated with LPS and nigericin. Immunoblot analysis of NLRP3 ubiquitination in cell lysates immunoprecipitated with a control IgG or anti-NLRP3 antibody. (**G** and **H**) HEK293T cells were transfected with increasing amounts of Flag-tagged human USP13 along with Myc-tagged human NLRP3 or its mutants and then analyzed by Western blotting. (**I**) HEK293T cells transfected with Myc-tagged human NLRP3 (WT) or Myc-tagged human NLRP3 (K192R and K496R) mutant were treated with CHX for indicated times. Immunoblot analysis of NLRP3 protein levels in cell lysates. (**J**) HEK293T cells were transfected with increasing amounts of Flag-tagged human USP13 together with Myc-tagged human NLRP3 (WT) or Myc-tagged human NLRP3 (K192R and K496R) mutant. Immunoblot analysis of NLRP3 protein levels in cell lysates. (**K**) HEK293T cells transfected with the indicated combinations of plasmids were treated with MG132 for 6 hours before collection. Immunoblot analysis of NLRP3 ubiquitination in cell lysates immunoprecipitated with anti–c-Myc beads. Data are representative of three independent experiments [(A) to (K)] or presented as the means ± SEM from three independent experiments [(G) to (J)]. Statistical significance was assessed by two-way ANOVA with Bonferroni’s multiple comparisons test [(G) to (J)].

It has been shown that NLRP3 can be degraded by both the ubiquitin-proteasome pathway and autophagy-lysosome pathway (*7*). We observed that the NLRP3 protein level in *Usp13*^−/−^ BMDMs was potently rescued by the treatment with MG132 (a proteasome inhibitor) but not E-64 (a lysosome inhibitor) (Fig. 3A). A time-gradient treatment with MG132 potently increased the NLRP3 protein level in *Usp13*^−/−^ BMDMs (Fig. 3B). These results suggested that USP13 may prevent NLRP3 degradation by the ubiquitin-proteasome pathway. Thus, we next investigated the role of USP13 in NLRP3 ubiquitination. In HEK293T cells, the forced expression of hemagglutinin (HA)-ubiquitin robustly triggered NLRP3 ubiquitination, while USP13 overexpression markedly diminished the ubiquitination of NLRP3 (Fig. 3C). Overexpression of USP13 selectively decreased K48-linked polyubiquitination of NLRP3 without affecting its K63-linked polyubiquitination (Fig. 3D). Consistently, deletion of USP13 specifically increased the K48-linked but not K63-linked ubiquitination of NLRP3 in resting murine BMDMs (Fig. 3E). LPS priming potently reduced NLRP3 K48-linked polyubiquitination in BMDMs, while this process was attenuated by USP13 deficiency (Fig. 3F). Together, USP13 stabilizes NLRP3 by preventing its K48-linked ubiquitination and degradation.

We further identified which lysine residues in NLRP3 may be associated with the regulation by USP13. By IP-MS analysis, we obtained 19 potential lysine ubiquitination sites on human NLRP3 protein. To determine which lysine sites are responsible for K48-linked ubiquitination of NLRP3, we replaced each of the 19 lysine sites with arginine residue and assessed the mutants for K48-linked ubiquitination. WT NLRP3 cotransfected with K48-only ubiquitin (a ubiquitin mutant that only forms K48-linked chains) in HEK293T cells exhibited a high level of polyubiquitination, while the ubiquitination of three mutants K192R, K496R, and K689R was substantially reduced (fig. S2, A to D). These data suggested that K48-linked polyubiquitin chains were mainly conjugated to K192, K496, and K689 residues of human NLRP3. In line with our results, K496 and K689 residues have been reported to be NLRP3 ubiquitination acceptor sites and are involved in NLRP3 degradation. Furthermore, we observed that the stabilizing effect of USP13 overexpression on the NLRP3 K192R mutant was sharply reduced, while a moderate stabilizing effect on the K496R mutant and a full stabilizing effect on the K689R mutant were retained when cotransfected in HEK293T cells (Fig. 3, G and H, and fig. S3). We thus generated an NLRP3 K192R and K496R double mutant. Our results showed that K192R/K496R double mutation profoundly extended the half-life of NLRP3 protein and USP13 almost lost the ability to stabilize this mutant (Fig. 3, I and J). When transfected together with K48-only ubiquitin in HEK293T cells, the NLRP3 K192R mutant only showed a mild level of K48-linked polyubiquitination and the K496R mutant showed a moderate level of K48-linked polyubiquitination, while K192R/K496R double mutation almost completely blocked NLRP3 K48-linked polyubiquitination (Fig. 3K). Overexpression of USP13 could still decrease the ubiquitination of NLRP3 K192R and K496R point mutants but had no effect on the K192R/K496R double mutant (Fig. 3K). Collectively, these results suggested that USP13 inhibits the K48-linked ubiquitination at the K192 and K196 sites of human NLRP3, thereby enhancing its protein stability.

### USP13 restrains TRIM31-mediated NLRP3 ubiquitination and degradation

**Fig. 4.**
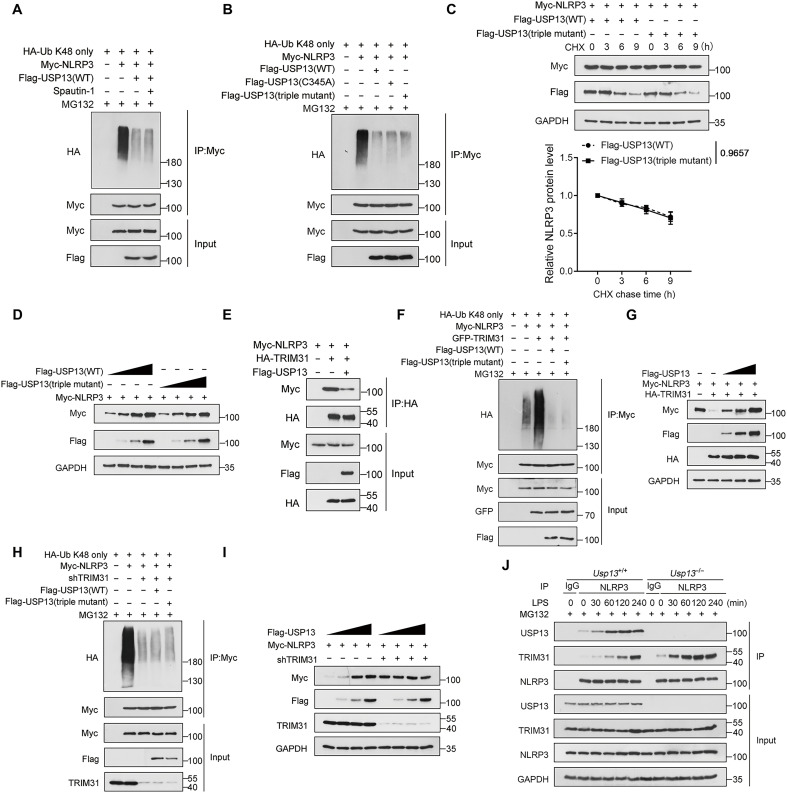
USP13 restrains TRIM31-mediated NLRP3 ubiquitination and degradation. (**A** and **B**) HEK293T cells transfected with the indicated plasmid combinations were treated with (A) or without (B) spautin-1 (10 μM) for 6 hours. Before collection, cells were treated with MG132 for 6 hours. Immunoblot analysis of NLRP3 ubiquitination in cell lysates immunoprecipitated with anti–c-Myc beads. (**C** and **D**) HEK293T cells transfected with the indicated plasmid combinations were treated with CHX for indicated times (C) or not (D). Immunoblot analysis of NLRP3 protein levels. (**E**) HEK293T cells were transfected with the indicated plasmid combinations. Immunoblot analysis of NLRP3 and TRIM31 in cell lysates immunoprecipitated with anti-HA beads. (**F**) HEK293T cells transfected with the indicated plasmid combinations were treated with MG132 for 6 hours before collection. Immunoblot analysis of NLRP3 ubiquitination in cell lysates immunoprecipitated with anti–c-Myc beads. (**G**) Immunoblot analysis of NLRP3, USP13, and TRIM31 protein levels in HEK293T cells transfected with the indicated plasmid combinations. (**H**) HEK293T cells transduced with lentiviruses expressing shCtrl or shTRIM31 were transfected with the indicated plasmid combinations. Before collection, cells were treated with MG132 for 6 hours. Immunoblot analysis of NLRP3 ubiquitination in cell lysates immunoprecipitated with anti–c-Myc beads. (**I**) HEK293T cells transduced with lentiviruses expressing shCtrl or shTRIM31 were transfected with the indicated plasmid combinations. Immunoblot analysis of NLRP3 protein levels in cell lysates. (**J**) *Usp13*^+/+^ and *Usp13*^−/−^ BMDMs were treated with MG132 for 6 hours and then stimulated with LPS for indicated times. Immunoblot analysis of NLRP3, TRIM31, and USP13 in cell lysates immunoprecipitated with an anti-NLRP3 antibody. Data are representative of three independent experiments [(A) to (J)] or presented as the means ± SEM from three independent experiments (C). Statistical significance was assessed by two-way ANOVA with Bonferroni’s multiple comparisons test (C).

We next tested whether USP13 regulates NLRP3 ubiquitination relying on its DUB activity. Unexpectedly, we found that treatment with spautin-1, an inhibitor of USP13 enzyme activity, could not reverse the inhibition of NLRP3 ubiquitination by USP13 (Fig. 4A). We further constructed two human USP13 catalytically inactive mutants, a single C345A mutant and a triple C345A/H814A/H823A mutant, as previously reported (*35*). The results showed that these two mutants were still able to diminish NLRP3 ubiquitination as WT USP13 did (Fig. 4B). In addition, the promotive effect of the USP13 triple mutant on NLRP3 protein stability was comparable to WT USP13 in HEK293T cells (Fig. 4, C and D). These data indicated that the deubiquitinating enzyme activity is not required for USP13 to regulate NLRP3 stability.

Our above results have demonstrated that USP13 promotes NLRP3 stability depending on their interaction; we thus hypothesized that USP13 may disrupt the interaction between NLRP3 and its E3 ligases. We have confirmed that USP13 binds to the PYD of NLRP3, which has been shown to be required for the binding of TRIM31 and FBXL2, two reported NLRP3 E3 ligases (*13*, *14*). By coimmunoprecipitation assays, we observed that USP13 overexpression inhibited the interaction between NLRP3 and TRIM31 but not FBXL2 (Fig. 4E and fig. S4A). Overexpression of human TRIM31 led to robust ubiquitination of WT NLRP3 when coexpressed in HEK293T cells. Although K192R and K496R mutation potently reduced NLRP3 K48-linked ubiquitination, TRIM31 overexpression still partially reversed the ubiquitination of these two mutants (fig. S4B). However, TRIM31 could not catalyze the polyubiquitination of the NLRP3 K192R/K496R double mutant and has no effect on the stability of this mutant (fig. S4, B and C). These data indicated that TRIM31 regulates NLRP3 ubiquitination on K192 and K496 residues. In line with our results, NLRP3 K496 has been reported to be a TRIM31 ubiquitination site, while FBXL2 facilitates ubiquitin ligation to K689 within the NLRP3 protein (*14*, *36*). We further observed that the forced expression of either WT USP13 or USP13 enzymatically inactive mutant potently blocked TRIM31-catalyzed NLRP3 ubiquitination (Fig. 4F). Overexpression of USP13 dose-dependently reversed NLRP3 degradation mediated by TRIM31 (Fig. 4G). In contrast, the knockdown of TRIM31 in HEK293T cells potently reduced NLRP3 K48-linked polyubiquitination and promoted its stability, while USP13 overexpression lost the ability to decrease NLRP3 ubiquitination and enhance NLRP3 protein stability in TRIM31 knockdown cells (Fig. 4, H and I). In murine BMDMs, LPS priming induced a rapid binding of USP13 to NLRP3 but a relatively later binding of TRIM31 to NLRP3; however, USP13 deficiency resulted in an early and robust interaction between TRIM31 and NLRP3 (Fig. 4J). Together, these results demonstrated that USP13 competes with TRIM31 to interact with NLRP3, thereby preventing TRIM31-mediated NLRP3 ubiquitination and degradation.

### USP13 facilitates NLRP3 inflammasome activation in macrophages

**Fig. 5.**
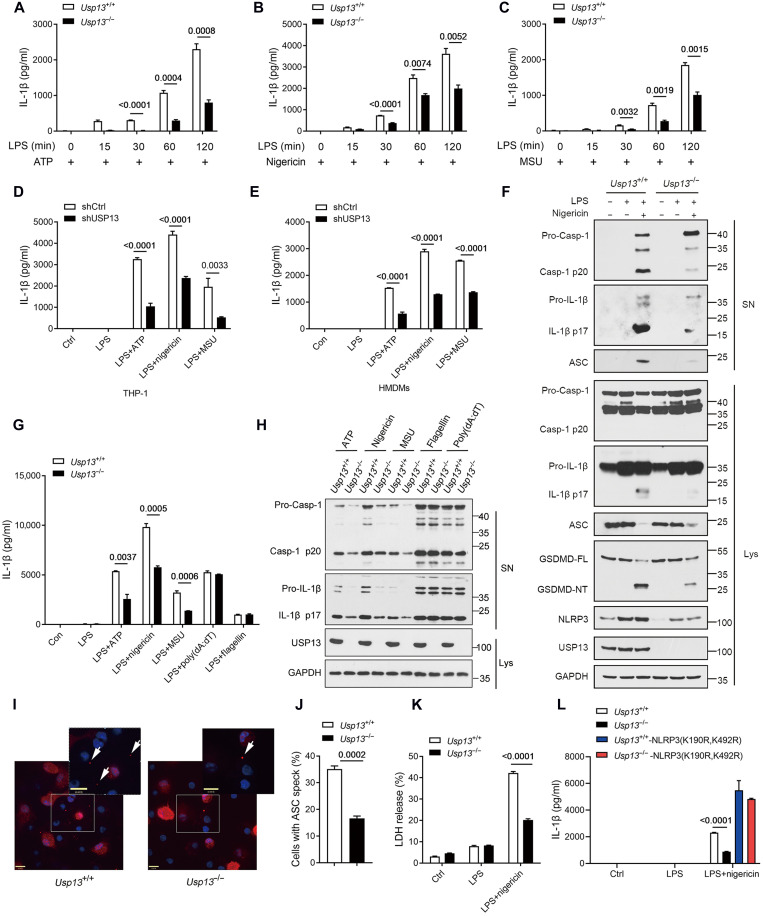
USP13 facilitates NLRP3 inflammasome activation in macrophages. (**A** to **C**) Cytometric bead array (CBA) analysis of IL-1β secretion from *Usp13*^+/+^ and *Usp13*^−/−^ BMDMs primed with LPS for indicated times before stimulation with ATP (A), nigericin (B), or MSU (C). (**D** and **E**) ELISA analysis of IL-1β from human THP-1 macrophages (D) or HMDMs (E) transduced with lentiviruses expressing shCtrl or shUSP13 before stimulation with LPS and ATP, nigericin, or MSU. (**F**) Immunoblot analysis of indicated proteins in culture supernatants and cell lysates from LPS-primed *Usp13*^+/+^ and *Usp13*^−/−^ BMDMs treated with nigericin. (**G**) CBA analysis of IL-1β secretion from LPS-primed *Usp13*^+/+^ and *Usp13*^−/−^ BMDMs before stimulation with ATP, nigericin, MSU, poly(dA:dT), or flagellin. (**H**) Immunoblot analysis of cleaved caspase-1 and IL-1β in culture supernatants from LPS-primed *Usp13*^+/+^ and *Usp13*^−/−^ BMDMs treated with ATP, nigericin, MSU, poly(dA:dT), or flagellin. (**I** and **J**) Immunostaining of intracellular ASC specks in LPS-primed *Usp13*^+/+^ and *Usp13*^−/−^ BMDMs treated with nigericin (I). Scale bars, 10 μm. Quantification of ASC specks from (I) was performed by counting cells in five random areas of each image in triplicate experiments and described as a percentage of ASC specks for total cell nuclei (J). At least 100 cells from each treatment condition were quantified. (**K**) Analysis of LDH in supernatants of untreated or LPS-primed BMDMs stimulated with nigericin. (**L**) *Usp13*^+/+^ and *Usp13*^−/−^ BMDMs transduced with control or mouse NLRP3 (K190R and K492R) mutant overexpression lentiviruses were primed with LPS and stimulated with nigericin. CBA analysis of IL-1β in the culture supernatants. Data are representative of three independent experiments [(F), (H), and (I)] or presented as the means ± SEM from three independent experiments [(A) to (E), (G), and (J) to (L)]. Statistical significance was assessed by two-way ANOVA with Bonferroni’s multiple comparisons test [(A) to (C)] or two-tailed unpaired *t* test [(D), (E), (G), and (J) to (L)].

We further defined whether the positive regulation of NLRP3 stability by USP13 might have any effect on the activation of the NLRP3 inflammasome in macrophages. WT and *Usp13*^−/−^ BMDMs were primed with LPS for various times and then stimulated with three different NLRP3 inflammasome activators, ATP, nigericin, and MSU. The results showed that IL-1β release in WT BMDMs was time-dependently increased by LPS priming, with a small amount at 15 to 30 min but remarkably increased at 60 to 120 min; however, USP13 deficiency potently inhibits IL-1β release at all the tested time points (Fig. 5, A to C). Consistently, the knockdown of USP13 significantly reduced IL-1β release in THP-1 cells and HMDMs treated with LPS and ATP, nigericin, or MSU (Fig. 5, D and E). LPS- and nigericin-induced generation of mature IL-1β and the p20 subunit of activated caspase-1 in the supernatant of BMDMs was prevented by USP13 deficiency (Fig. 5F and fig. S5A). Moreover, USP13 deficiency selectively prevented IL-1β production, as well as pro–caspase-1 and pro–IL-1β cleavage, triggered by the NLRP3 inflammasome–specific activators but not the AIM2 (absent in melanoma 2) inflammasome activator flagellin and the NLRC4 inflammasome activator poly(deoxyadenylic-deoxythymidylic) acid sodium salt [poly(dA:dT)] in LPS-primed BMDMs (Fig. 5, G and H), which indicates that USP13 specifically regulates NLRP3 inflammasome activation. We also found that the formation of ASC specks, a hallmark of inflammasome activation, was potently reduced in *Usp13*^−/−^ BMDMs treated with LPS and nigericin (Fig. 5, I and J). Accordingly, USP13 deficiency inhibited NLRP3-mediated pyroptosis in LPS-primed BMDMs stimulated with nigericin, as manifested by the reduced cleavage of GSDMD and the release of lactate dehydrogenase (LDH) (Fig. 5, F and K, and fig. S5A). Together, our above results demonstrated that USP13 is essential for the optimal activation of the NLRP3 inflammasome. Furthermore, the forced expression of the mouse NLRP3 K190R/K492R double mutant lacking K48-linked polyubiquitination (corresponding to the human K192R/K496R mutant) effectively reversed IL-1β release in *Usp13*^−/−^ BMDMs (Fig. 5L and fig. S6, A and B), which confirmed that USP13 regulates NLRP3 inflammasome activation by preventing NLRP3 ubiquitination and degradation.

### USP13 deficiency inhibits NLRP3 inflammasome activation in vivo

**Fig. 6.**
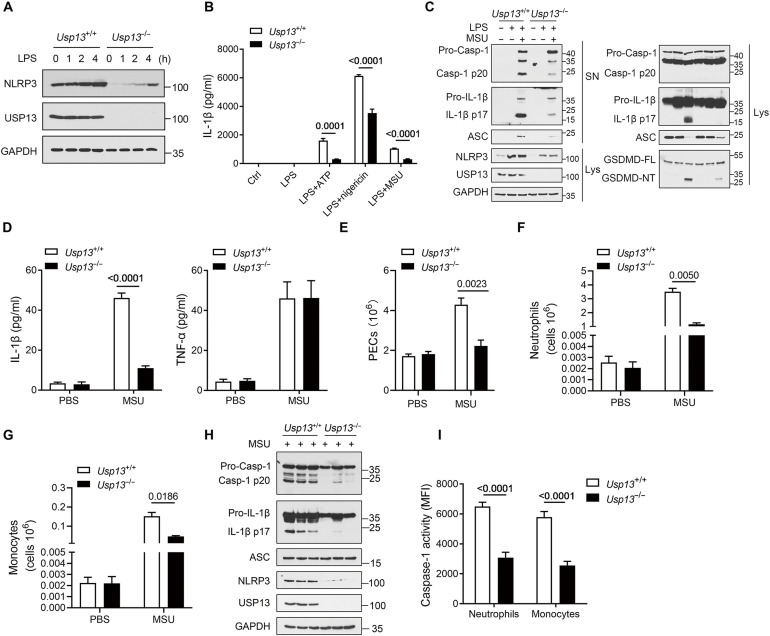
USP13 deficiency inhibits NLRP3 inflammasome activation in vivo. (**A**) Immunoblot analysis of NLRP3 protein levels in *Usp13*^+/+^ and *Usp13*^−/−^ PRMs treated with LPS for indicated times. (**B**) CBA analysis of IL-1β secretion from *Usp13*^+/+^ and *Usp13*^−/−^ PRMs stimulation with LPS and ATP, nigericin, or MSU. (**C**) Immunoblot analysis of indicated proteins in culture supernatants and cell lysate from LPS-primed *Usp13*^+/+^ and *Usp13*^−/−^ PRMs treated with MSU. (**D** to **I**) *Usp13*^+/+^ and *Usp13*^−/−^ mice were intraperitoneally injected with MSU (1 mg per mouse) or vehicle (PBS) for 4 hours. *n* = 6 per group. ELISA of IL-1β and TNF-α in the peritoneal lavage fluid (D). Flow cytometric analysis of PECs [(E) to (G)]. Immunoblot analysis of cleaved caspase-1, cleaved IL-1β, and NLRP3 protein levels in PECs (H). Flow cytometric analysis of caspase-1 activity in peritoneal exudate monocytes and neutrophils (I). MFI, mean fluorescence intensity. Data are representative of three independent experiments [(A), (C), and (H)] or presented as the means ± SEM from three independent experiments (B) or six biological replicates [(D) to (G) and (I)]. Statistical significance was assessed by a two-tailed unpaired *t* test [(B), (D) to (G), and (I)].

The MSU-induced peritonitis model was used to investigate the regulatory role of USP13 in the activation of the NLRP3 inflammasome in vivo, which is a well-established NLRP3-dependent acute inflammatory model characterized by IL-1β secretion and massive neutrophil influx into the peritoneal cavity (*37*). As the PRMs contribute to the initial response to MSU and are the key resource of IL-1β (*38*), we first examined the effect of USP13 deficiency on NLRP3 inflammasome activation in PRMs. Like *Usp13*^−/−^ BMDMs, *Usp13*^−/−^ PRMs showed potently reduced NLRP3 protein levels regardless of whether treated with LPS or not (Fig. 6A). USP13 deficiency profoundly inhibited IL-1β release in LPS-primed PRMs stimulated with either ATP, nigericin, or MSU (Fig. 6B). The cleavage of pro–caspase-1, pro–IL-1β, and GSDMD induced by LPS and MSU was also prevented by USP13 ablation (Fig. 6C and fig. S5B). Therefore, these data verified that USP13 deficiency inhibited NLRP3 inflammasome activation by reduced NLRP3 stability in murine PRMs.

Next, we injected MSU into the peritoneum of *Usp13*^+/+^ and *Usp13*^−/−^ mice. As expected, MSU injection induced significant increases in IL-1β and TNF-α in the peritoneal lavage fluid, while USP13 deficiency selectively reduced the release of IL-1β but not TNF-α (Fig. 6D). Accordingly, the peritoneal recruitment of leukocytes, especially the Ly6G^+^ neutrophils and Ly6C^+^ monocytes, was remarkably decreased in *Usp13*^−/−^ mice by flow cytometry analysis (Fig. 6, E to G). In addition, we observed potently reduced levels of NLRP3 protein, as well as cleaved caspase-1 and IL-1β, in the lysates of peritoneal exudate cells (PECs) from *Usp13*^−/−^ mice injected with MSU compared to control mice (Fig. 6H). The caspase-1 activity in infiltrated neutrophils and monocytes was also inhibited by USP13 deficiency (Fig. 6I). These data indicated that USP13 ablation may decrease NLRP3 stability and inflammasome activation in multiple immune cells. Collectively, our findings confirmed that USP13 is critical for NLRP3 protein stability and inflammasome activation in vivo.

## DISCUSSION

In this study, we confirmed the essential role of USP13 in the regulation of NLRP3 stability and NLRP3 inflammasome activation. Our results showed that USP13 binds to the PYD of NLRP3 protein and prevents NLRP3 K48-linked polyubiquitination on K192 and K496 residues, which in turn inhibits NLRP3 proteasomal degradation. We found that USP13 stabilizes NLRP3 in a DUB activity–independent manner by competing with TRIM31, a well-known E3 ligase of NLRP3, to bind to the PYD of NLRP3 (fig. S7). In resting macrophages, USP13 displays a weak interaction with NLRP3 to maintain a basic level of NLRP3 protein. In response to LPS treatment, the interaction between USP13 and NLRP3 rapidly increases in the early phase, while the interaction between TRIM31 and NLRP3 potently increases at relatively later time points. Thus, we proposed that USP13 functions in the initiation of NLRP3 inflammasome activation, while TRIM31 prevents the overactivation of the NLRP3 inflammasome at later stages. USP13 deficiency leads to a rapid and potent binding of TRIM31 to NLRP3, resulting in NLRP3 degradation, which subsequently reduces NLRP3 inflammasome assembly and activation. USP13-deficient human and mouse macrophages show decreased IL-1β production induced by NLRP3-specific ligands. In particular, USP13 ablation in mice attenuated MSU-induced peritonitis, as manifested by decreased IL-1β release and inflammatory cell infiltration. Collectively, our study provided the first in vivo evidence confirming that USP13 promotes NLRP3 stability and inflammasome activation. Besides, USP13 has been reported to be a key enzyme for paxillin-mediated NLRP3 deubiquitination and NLRP3 inflammasome activation upon ATP treatment (*28*). Treatment with the USP13 inhibitor spautin-1 inhibits NLRP3 inflammasome activation in oxygen-glucose deprivation/reoxygenation–treated PC12 cells (*29*) but instead enhances NLRP3 inflammasome activation in mouse coronary artery smooth muscle cells treated with 7-ketocholesterol (*39*). These studies indicate that USP13 may regulate NLRP3 inflammasome activation through distinct mechanisms under different conditions.

In the resting state, NLRP3 is ubiquitylated and its protein level is relatively low to avoid aberrant inflammasome assembly and activation. Multiple E3 ligases are involved in NLRP3 ubiquitination and degradation, such as MARCH7, TRIM31, FBXL2, Cbl-b, β-TrCP1, and so on (*7*, *10*, *12*–*15*). However, a rapid activation of the NLRP3 inflammasome is critical for host defense against pathogens. In macrophages, several hours of stimulation is required to achieve optimal levels of NLRP3 transcription. Therefore, antagonization of NLRP3 ubiquitination to rapidly increase the NLRP3 protein level is of great importance in the early stage of NLRP3 inflammasome activation. Several DUBs are reported to regulate NLRP3 deubiquitination and stability, including OTUD6A, USP19, and the UAF1/USP1 complex (*11*, *16*, *17*). Here, we showed that USP13 competes with TRIM31 to interact with NLRP3, which consequently prevents NLRP3 K48-linked polyubiquitination and proteasomal degradation. Our results revealed an additional layer of the regulatory mechanism of NLRP3 ubiquitination by targeting the interaction between NLRP3 and its E3 ubiquitin ligases. Similarly, USP5 serves as a key scaffold protein recruiting the E3 ligase MARCH7 to NLRP3 and promoting the autophagic degradation of NLRP3 (*40*).

Up until now, four lysine residues, K380, K430, K496, and K689, have been identified to be involved in NLRP3 degradation. β-TrCP1 increases the proteasomal degradation of NLRP3 via K27-linked ubiquitination at K380 (*10*). TRIM31 and Cbl-b target NLRP3 at K496 for K48-linked ubiquitination and proteasome-mediated degradation (*15*, *36*). FBXL2 mediates NLRP3 proteasomal degradation by catalyzing NLRP3 ubiquitination at K689 (*14*). In contrast, OTUD6A directly binds to the NACHT domain of NLRP3 and selectively removes K48-linked polyubiquitin chains from NLRP3 at K430 and K689 to stabilize NLRP3 (*17*). USP19 inhibits the proteasomal degradation of NLRP3 by specifically cleaving the K6-linked polyubiquitin chain of NLRP3 at K689 (*11*). Our study revealed that K192 and K496 residues are required for TRIM31-mediated NLRP3 ubiquitination. In particular, the K192 residue is a previously unknown site for NLRP3 K48-linked polyubiquitination. In addition, the LRR domain (amino acids 742 to 991) is the key region of NLRP3 for MARCH7-mediated K48-linked ubiquitination and autophagic degradation; however, the exact ubiquitination sites in this region are still uncovered (*12*). Therefore, further studies are needed to fully reveal the ubiquitination sites associated with NLRP3 degradation.

USP13 has been shown to play a critical role in the regulation of inflammatory responses. USP13 deubiquitinates and stabilizes IL-1R8/Sigirr, a negative regulator of IL-1 and TLR signaling, to dampen inflammatory responses. USP13 deficiency enhances LPS-induced TLR4 signaling and cytokine release, leading to increased susceptibility to LPS-induced inflammatory lung injury in mice (*23*). Besides, USP13 inhibits the LPS-induced production of multiple inflammatory cytokines by deubiquitinating and inactivating IRAK4 and thus negatively regulates LPS-induced mouse sepsis shock (*24*). USP13 ameliorates nonalcoholic steatohepatitis by targeting Irhom2 or TAK1. USP13 removes the K63 ubiquitination of Irhom2, resulting in a decrease in Irhom2 stability, consequently suppressing mitogen-activated protein kinase and nuclear factor κB activation (*26*). USP13 catalyzes the K63-linked deubiquitination of TAK1 and reduces TAK1 activation, thereby dampening downstream inflammatory pathways (*27*). USP13 directly binds to and stabilizes PTEN, which alleviates inflammation, apoptosis, and oxidative stress in osteoarthritis (*25*). These studies highlight the anti-inflammatory properties of USP13. In contrast, USP13 also has pro-inflammation activity. USP13 positively regulates type I and type II interferon signaling by deubiquitinating and stabilizing the STAT1 (signal transducers and activators of transcription 1) protein (*41*). USP13 interacts with TLR4 and inhibits the ubiquitin-mediated degradation of TLR4 (*42*). USP13 promotes HMGB1 (high-mobility group box 1) stability and secretion through its DUB activity (*43*). USP13 is recruited by paxillin to remove the K63 ubiquitination of NLRP3 and promotes NLRP3 inflammasome activation upon ATP treatment (*28*). Our present study also showed a pro-inflammatory role of USP13 by stabilizing NLRP3 and enhancing NLRP3 inflammasome activation. Typically, USP13 functions through its DUB activity. However, our study reveals that USP13 can exert its function in a DUB activity–independent manner. Collectively, these studies indicate the diversity and complexity of USP13’s regulatory role in inflammatory responses, which is worthy of further investigation.

In summary, our study uncovers a previously unrecognized mechanism by which USP13 regulates NLRP3 stability and inflammasome activation. USP13 competes with TRIM31 to bind to NLRP3, which in turn prevents TRIM31-mediated NLRP3 ubiquitination and proteasomal degradation. USP13 may serve as a promising target for manipulating NLRP3 inflammasome activation.

## MATERIALS AND METHODS

### Mice

*Usp13*^−/−^ mice were a gift from L.-Q. Zhang (Beijing Institute of Lifeomics, Beijing, China), which were generated by CRISPR-Cas9 technology on a C57BL/6J background, harboring a short frameshifting deletion in exon 1. All these mouse strains were maintained under specific pathogen–free conditions in a controlled environment (12-hour light/dark cycle; 25 ± 2°C) at the Animal Facility of our institute. Both male and female mice aged 8 to 10 weeks were used in this study. All animal experiments were reviewed and approved by the Institutional Animal Care and Use Committee of our institution (no. IACUC-DWZX-2020-568).

### Reagents and antibodies

Ultrapure LPS (tlrl-pb5lps), Pam3CSK4 (tlrl-pms), ATP (tlrl-atp), nigericin (tlrl-nig), MSU (tlrl-msu), flagellin (tlrl-epstfla), and poly(dA:dT) (tlrl-patn-1) were purchased from Invivogen. CHX (2112) and MG132 (2194) were obtained from Cell Signaling Technology. E-64 (HY-15282) and spautin-1 (HY-12990) were purchased from MedChemExpress. Phorbol 12-myristate 13-acetate (PMA; P8139) was purchased from Sigma-Aldrich. Antibodies used were as follows: rabbit monoclonal anti-NLRP3 (1:1,000, Cell Signaling Technology, 15101), rabbit polyclonal anti-USP13 (1:500, ABclonal, A10264), rabbit polyclonal anti-TRIM31 (1:1000, ABclonal, A10639), mouse monoclonal anti-Flag (1:4,000, Sigma, F3165), rabbit monoclonal anti-Flag (1:2,000, Cell Signaling Technology, 14793), mouse monoclonal anti-Myc (1:2,000, ABclonal, AE010), rabbit monoclonal anti-Myc (1:2,000, ABclonal, AE070), mouse monoclonal anti-HA (1:2,000, Santa Cruz, sc-7392), mouse monoclonal anti-GFP (1:2000, ABclonal, AE012), mouse monoclonal anti-Ubiquitin (1:500, BostonBiochem, A-104), rabbit monoclonal anti–K48-Ubiquitin (1:500, Cell Signaling Technology, 4289), rabbit monoclonal anti–K63-Ubiquitin (1:500, Cell Signaling Technology, 5621), mouse monoclonal anti-mouse caspase-1 (1:1,000, Adipogen, AG-20B-0042), rabbit monoclonal anti-GSDMD (1:1000, Abcam, ab209845), rabbit polyclonal anti-ASC (1:1,000, Adipogen, AG-25B-0006), goat polyclonal anti–mouse-IL-1β (1:4,000, R&D Systems, AF-401-NA), and rabbit monoclonal anti–GAPDH (glyceraldehyde-3-phosphate dehydrogenase) (1:5,000, ABclonal, AC002). Secondary horseradish peroxidase–conjugated antibodies used were goat anti-mouse IgG (1:5,000, Santa Cruz, sc-2005), goat anti-rabbit IgG (1:5,000, Santa Cruz, sc-2004), and bovine Anti-Goat IgG (1:2,000, Jackson ImmunoResearch, 805-035-180). Alexa Fluor 594–conjugated Goat anti-rabbit IgG (1:2,000, A-11012) was from Invitrogen.

### Cell culture

To obtain mouse BMDMs, bone marrow cells were isolated and cultured in RPMI 1640 medium complemented with 10% heated inactivated fetal bovine serum (FBS) (Gibco, 10270), 1% penicillin/streptomycin, and 30% L929 culture supernatant for 7 days. To obtain mouse primary peritoneal macrophages, C57BL/6J mice were injected intraperitoneally with 5 ml of ice-cold RPMI 1640 medium (with 5 mM EDTA). PECs were harvested and washed two times with ice-cold RPMI 1640 medium to remove EDTA. PECs were resuspended and seeded in 12-well plates. Two hours later, nonadherent cells were discarded and adherent monolayer cells were used as primary peritoneal macrophages. THP-1 cells were maintained in RPMI 1640 medium supplemented with 10% FBS and 50 μM 2-mercaptoethanol. Differentiated THP-1 cells were acquired by coculturing with 100 nM PMA (Sigma-Aldrich, P8139) for 3 hours. After 3 hours, media were removed and fresh media were added. Experiments were carried out the following day. HMDMs were generated from CD14^+^ monocytes by a 7-day cultivation with human macrophage colony-stimulating factor (Peprotech, 300-25). Human umbilical cord blood units were donated by healthy mothers. All participants provided their written informed consent. All investigations were approved by the Institutional Research Ethics Committee of our institute. HEK293T cells were cultured in Dulbecco’s modified Eagle’s medium supplemented with 10% FBS and 1% penicillin/streptomycin. Cells were incubated in a humidified chamber with 5% CO_2_ at 37°C for growth.

### Plasmids and transfection

Myc-mouse NLRP3 was a gift from T. Li (National Center of Biomedical Analysis, Beijing, China). Myc-human NLRP3, Myc-human ASC, and Myc-human pro–caspase-1 plasmids were provided by J. Wang (Beijing Proteome Research Center, China). Flag-human USP13, HA-human TRIM31, GFP-human TRIM31, and GFP-human FBXL2 plasmids were purchased from Hunan Fenghui Biotechnology Co., Ltd. HA-ubiquitin, HA-ubiquitin K48-only (all lysines mutated to arginine except K48; forms only K48-linked chains), and HA-ubiquitin K63-only (all lysines mutated to arginine except K63; forms only K63-linked chains) plasmids were a gift from X.-M. Zhang (National Center of Biomedical Analysis, Beijing, China). Construction mutants of NLRP3 and USP13 were used by the KOD-Plus-Mutagenesis kit (Toyobo, Osaka, Japan). Human TRIM31 short hairpin RNAs (shRNAs) (shTRIM31: 5-GCTCTCAGGATACGAAGACAT-3), human USP13 shRNAs (shUSP13: 5-CGATTTAAATAGCGACGATTA-3), and scramble shRNAs (shCtrl: 5-CCTAA-GGTTAAGTCGCCCTCG-3) were cloned into the pLKO.1-Puro vector (Addgene). Lentiviral expression constructs for human Flag-NLRP3 and mouse NLRP3 (K190R and K492R) were cloned into the pCDH-MCS-T2A-copGFP-MSCV expression vector (pCDH-GFP; System Biosciences, CD523A-1). All constructs were confirmed by DNA sequencing. Transient transfections were performed using Lipofectamine 2000 Transfection Reagent (Invitrogen, 11668019), according to the manufacturer’s instructions, in HEK293T cells.

### Lentivirus production and infection

HEK293T cells were transfected with 15 μg of expression vectors together with 10 μg of psPAX2 (Addgene, 12260) and 5 μg of pMD2.G (Addgene, 12259) using Lipofectamine 3000 (Invitrogen, L3000015). The media were collected and centrifuged at 900*g* for 15 min at 4°C at 48 and 72 hours. The virus-containing supernatant was precipitated by PEG-*it* virus precipitation solution (System Biosciences, LV810A), resuspended in 100 μl of phosphate-buffered saline (PBS), and stored at −80°C. For lentivirus infection, cells were added with 5 or 15 MOI (multiplicity of infection) lentiviral particles in the presence of polybrene (8 μg/ml). After 12 hours, cells were washed and incubated with fresh media. On day 3, puromycin (1 μg/ml; Invivogen, ant-pr-1) was added to select target cells.

### Western blotting, immunoprecipitation, and ubiquitination assay

Cells were lysed with SDS sample buffer, and supernatants were concentrated with trichloroacetic acid and then analyzed directly by SDS–polyacrylamide gel electrophoresis immunoblotting. For immunoprecipitation assays, cells were washed with PBS and lysed in IP lysis buffer [150 mM NaCl, 100 mM tris-HCl (pH 8.0), 0.5% Triton X-100, and 2 mM EDTA] with a protease inhibitor for 45 min at 4°C. The lysates were sonicated and centrifuged at 12,000*g* at 4°C for 15 min. Supernatants were gently shaken and incubated with anti–c-Myc agarose (Sigma-Aldrich, A7470), anti-Flag M2 beads (Sigma-Aldrich, A2220), anti-HA beads (Beyotime, P2121), anti-GFP beads (Beyotime, P2132), or 2 to 4 μg of indicated primary antibody with protein A/G agarose (Santa Cruz Biotechnology, sc-2003) at 4°C overnight. The beads were washed three times with IP lysis buffer, and 50 μl of SDS sample buffer was added. For the ubiquitination assay, cells were washed with PBS and lysed in 100 μl of IP lysis buffer with 1% SDS, 50 μM *N*-ethylmaleimide (Sigma-Aldrich, E3876), and a protease inhibitor (Roche, 04693116001). Lysates were boiled for 10 min, sonicated, and added with 900 μl of IP lysis. After centrifugation, lysates were incubated with an anti–c-Myc agarose (Sigma-Aldrich, A7470) or anti-NLRP3 (Cell Signaling Technology, 15101) antibody with protein A/G agarose (Santa Cruz Biotechnology, sc-2003) at 4°C overnight. The beads were washed five times with wash buffer, and 50 μl of SDS sample buffer was added.

### Immunoprecipitation-mass spectrometry (IP-MS)

THP-1 cells expressing Flag-tagged NLRP3 were harvested and lysed with IP lysis buffer [150 mM NaCl, 100 mM tris-HCl (pH 8.0), 0.5% Triton X-100, and 2 mM EDTA] containing a protease inhibitor. Cell lysates were centrifuged at 12,000*g* for 30 min and incubated with anti-Flag M2 beads (Sigma-Aldrich, A2220) for 6 hours at 4°C. The resulting precipitates were washed five times with lysis buffer and then subjected to SDS–polyacrylamide gel electrophoresis. IP samples were reduced with 5 mM dithiothreitol, alkylated with 10 mM iodoacetamide, and digested with trypsin (1:50, w/w) at 37°C overnight. Tryptic peptides were desalted using C18 StageTips (Thermo Fisher Scientific, no. SP301) and were analyzed by LTQ-Orbitrap Velos (Thermo Fisher Scientific). The resulting MS/MS data were processed using Proteome Discoverer 2.4. Tandem mass spectra were searched against the UniProt Homo_sapiens database.

### CHX chase experiments

HEK293T cells transfected with various indicated plasmids and BMDM cells were treated with CHX (50 μg/ml; Cell Signaling Technology, 2112) for the indicated durations before collection. Cells were washed with ice-cold PBS, then lysed in 2× Laemmli sample buffer, and subjected to Western blot analysis.

### Inflammasome stimulation

Mouse peritoneal macrophages, BMDMs, or HMDMs were primed with LPS (100 ng/ml) for indicated times. Following this, the priming stimulus was removed. NLRP3 inflammasome activation was achieved as follows: ATP (2 mM) for 45 min, nigericin (10 μM) for 1 hour, and MSU (200 μg/ml) for 6 hours. NLRC4 inflammasome activation was achieved by transfection of recombinant flagellin (1 μg/ml) using PULSin (Polyplus-transfection, 501-01) for 12 hours. AIM2 inflammasome activation was achieved by transfection of poly(dA:dT) (1 μg/ml) using Lipofectamine 3000 (Invitrogen, L3000015) for 12 hours. Differentiated THP-1 cells were acquired by coculturing with 100 nM PMA for 3 hours. After overnight incubation, cells were primed with LPS (100 ng/ml) for 2 hours, followed by treatment similar to those described above.

### Measurement of cytokines

For cytokines in the cell culture medium with high detection levels, samples were diluted and analyzed with the Mouse IL-1β Enhanced Sensitivity Flex Set (BD Biosciences, 550232), Mouse TNF-α Flex Set (BD Biosciences, 558299), Mouse IL-6 Flex Set (BD Biosciences, 558301), and Human IL-1β ELISA Kit (Abcam, ab214025). For serum and peritoneal lavage fluid with low detection levels, samples were concentrated and analyzed with the Mouse IL-1β ELISA Kit (Abcam, ab197742) and Mouse TNF-α ELISA Kit (Abcam, ab208348) according to the manufacturer’s instructions.

### Immunofluorescence

BMDMs were seeded at 2 × 10^5^/ml on the 24-well glass-bottom plates, primed with LPS (100 ng/ml) for 2 hours, and stimulated with nigericin (10 μM) for 1 hour. After treatment, cells were washed three times with ice-cold PBS, fixed in 4% paraformaldehyde for 15 min, and then permeabilized with 0.25% Triton X-100 for 15 min at room temperature. The cells were blocked with 5% bovine serum albumin for 1 hour at room temperature and then incubated with rabbit polyclonal anti-ASC (1:100) overnight at 4°C. After washing three times, cells were incubated with an Alexa Fluor 594–conjugated Goat anti-rabbit IgG (1:2,000, Invitrogen, A-11012) secondary antibody for 1 hour at room temperature. Nuclei were costained with Hoechst 33342. Cells were visualized by a Zeiss LMS 710 confocal microscope.

### In vivo peritonitis

For induction of peritonitis, *Usp13*^+/+^ and *Usp13*^−/−^ mice (8 to 10 weeks old) were injected intraperitoneally with MSU (1 mg) in 200 μl of PBS and euthanized 4 hours after injection. Peritoneal cavities were lavaged with 5 ml of ice-cold PBS (with 5 mM EDTA). Peritoneal lavage fluid was centrifuged at 800*g* for 5 min. Cells were stained and analyzed by flow cytometry. The levels of IL-1β and TNF-α in lavage fluids were concentrated and detected by the enzyme-linked immunosorbent assay (ELISA).

### Flow cytometry

PECs were washed with PBS and incubated with Fixable Viability Dye eFluor 780 (eBioscience, 65-0865), CD45-PE (eBioscience, 12-0454), CD11b-efluor450 (eBioscience, 48-0112), Ly6C-PE-Cy7 (eBioscience, 25-5932), and Ly6G-APC (eBioscience, 17-9668) for 30 min at 4°C. Cells were gated first on live CD45^+^ cells. Inflammatory neutrophils were identified as CD45^+^CD11b^+^Ly-6G^+^. Inflammatory monocytes were identified as CD45^+^CD11b^+^Ly-6G^−^Ly6C^+^. The number of leukocytes (CD45^+^ cells) was counted by 123-count eBeads Counting Beads (eBioscience, 01-1234). Flow cytometric analysis was performed using a BD LSRFortessa (BD Biosciences), and at least 10,000 target cells were acquired.

### Statistical analysis

All data are presented as the means ± SEM. The distribution of levels was tested by the Kolmogorov-Smirnov test. The variance was similar in the groups being compared. Statistical analysis was carried out using a standard two-tailed unpaired Student’s *t* test for single comparisons and two-way analysis of variance (ANOVA) for multiple comparisons. Statistics were calculated with GraphPad Prism 7 (GraphPad Software). A *P* value <0.05 was considered to be a statistically significant difference.
