# Supplementary material for: USP13 stabilizes NLRP3 to facilitate inflammasome activation by preventing TRIM31-mediated NLRP3 ubiquitination and degradation
Source: Sci Adv. 2025 Sep 26;11(39):eadx3827. doi: 10.1126/sciadv.adx3827 (PMC12466843; doi:10.1126/sciadv.adx3827)

Science Advances
AAAS

Supplementary Materials for

USP13 stabilizes NLRP3 to facilitate inflammasome activation by preventing TRIM31-mediated NLRP3 ubiquitination and degradation

Ya-Ting Li *et al.*

Corresponding author: Xiao-Ming Yang, xiaomingyang@sina.com; Guang-Ming Ren, max19920503@163.com; Rong-Hua Yin, yrh1980110@126.com

Sci. Adv. **11**, eadx3827 (2025)
DOI: 10.1126/sciadv.adx3827

The PDF file includes:

Figs. S1 to S7
Legend for table S1
Legend for data S1
Data S2

Other Supplementary Material for this manuscript includes the following:

Table S1
Data S1

Supplementary Figures

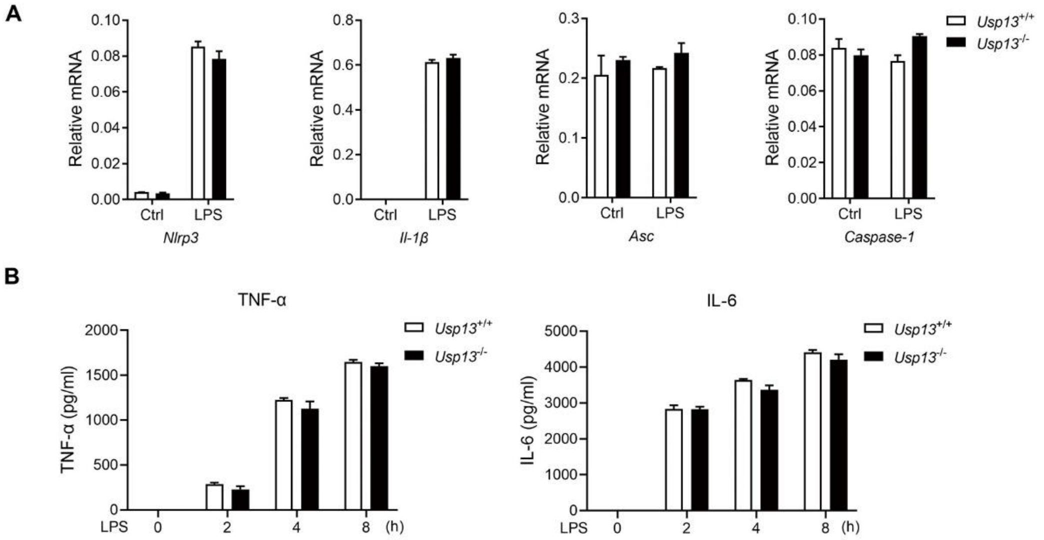

Supplementary Fig. 1. USP13 deficiency has no effect on LPS-induced transcription of NLRP3 and IL-1β and release of TNF-α and IL-6 in BMDMs. (**A**) Real-time PCR analysis of *Nlrp3*, *Il-1β*, *Asc*, and *Caspase-1* mRNA levels in *Usp13*$^{+/+}$ and *Usp13*$^{-/-}$ BMDMs stimulated with LPS for 3 h or not (normalized to *Gapdh* levels). (**B**) CBA analysis of TNF-α and IL-6 secretion in *Usp13*$^{+/+}$ and *Usp13*$^{-/-}$ BMDMs stimulated with LPS for indicated times. Data are presented as mean ± SEM from three independent experiments (A and B).

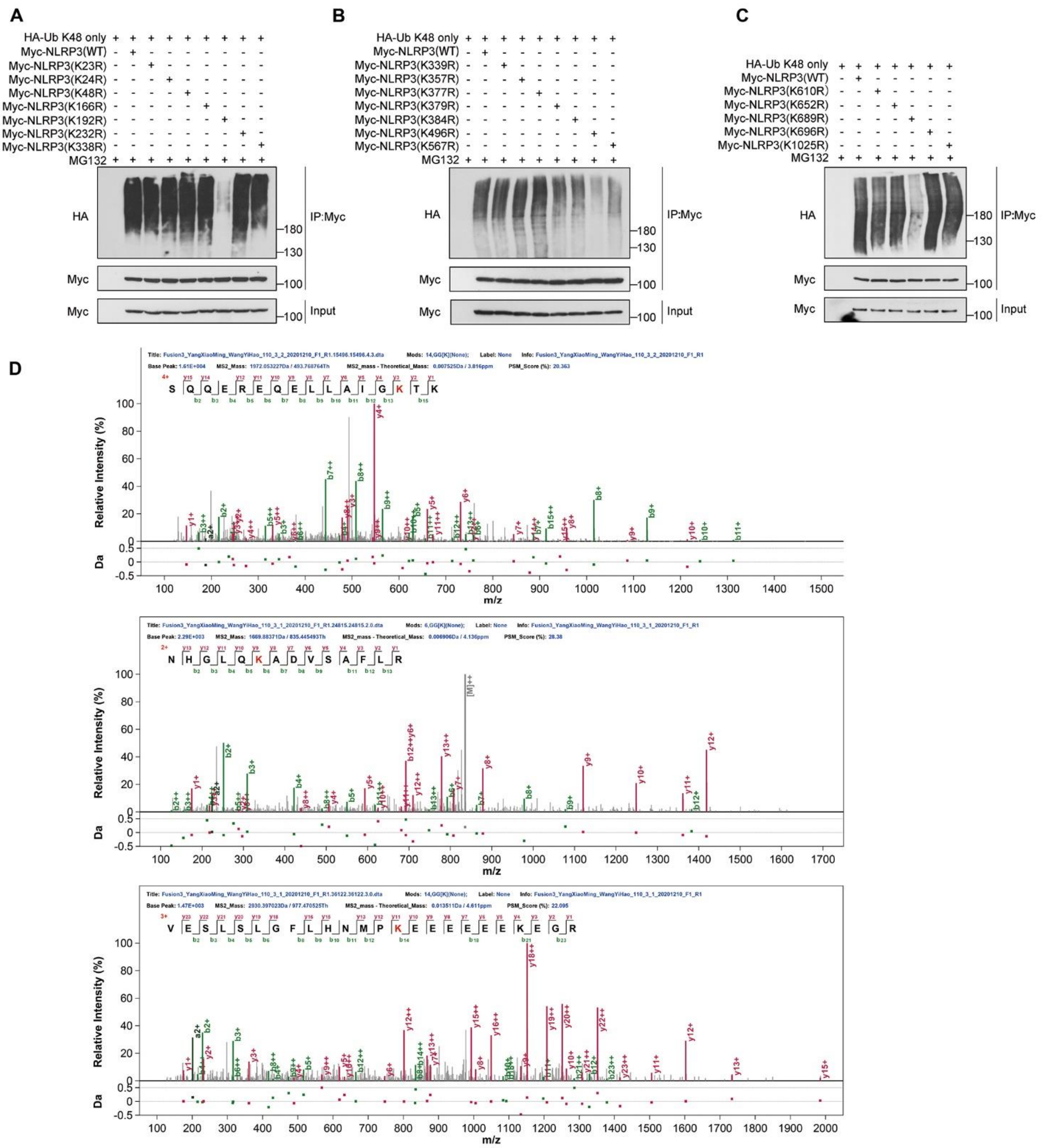

Supplementary Fig. 2. K192, K496, and K689 are the major residues of NLRP3 for K48-linked ubiquitination. (**A to C**) HEK293T cells were transfected with indicated combinations of plasmids. Before collection, cells were treated with MG132 (20 μM) for 6 h. Immunoblot analysis of NLRP3 K48-linked ubiquitination in cell lysates immunoprecipitated with anti-c-Myc beads. (**D**) Identification of NLRP3 ubiquitination sites (K192, K496, K689) by mass spectrometry. Data are representative of three independent experiments (A to C).

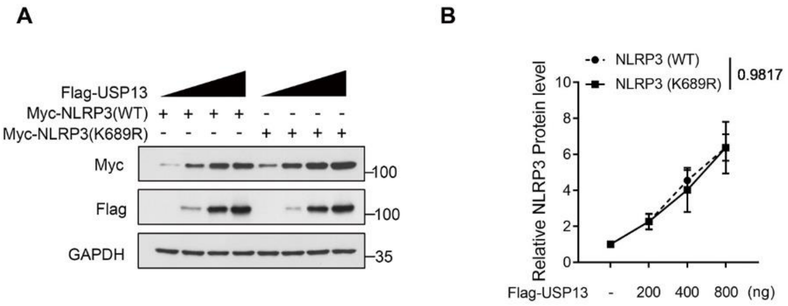

Supplementary Fig. 3. NLRP3 K689R mutation does not affect the stabilizing effect of USP13 on NLRP3. (**A and B**) HEK293T cells were transfected with increasing amounts of Flag-tagged human USP13 along with Myc-tagged human NLRP3 or NLRP3 (K689R) and then analyzed by western blot. Representative western blot and quantification of relative protein levels are shown. Data are representative of three independent experiments (A) or presented as mean ± SEM from three independent experiments (B). Statistical significance was assessed by two-way ANOVA with Bonferroni's multiple comparisons test (B).

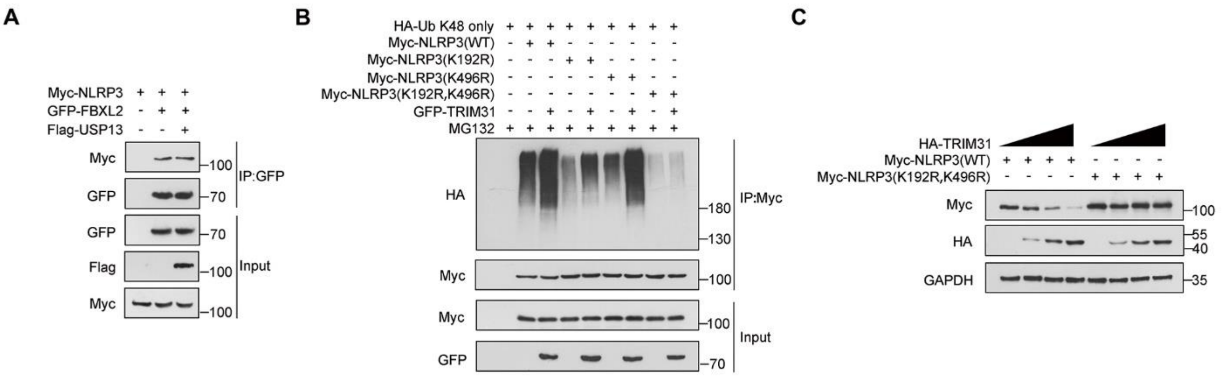

Supplementary Fig. 4. TRIM31 regulates NLRP3 ubiquitination on K192 and K496 residues. (**A**) HEK293T cells were transfected with indicated combinations of plasmids. Immunoblot analysis of NLRP3 and FBXL2 in cell lysates immunoprecipitated with anti-GFP beads. (**B**) HEK293T cells were transfected with various combinations of plasmids as indicated above the lanes. Before collection, cells were treated with MG132 for 6 h. Immunoblot analysis of NLRP3 ubiquitination in cell lysates immunoprecipitated with anti-c-Myc beads. (**C**) Immunoblot analysis of NLRP3 protein levels in HEK293T cells transfected with Myc-tagged human NLRP3 (WT), Myc-tagged human NLRP3 (K192R, K496R), and increasing amounts of HA-tagged human TRIM31. Data are representative of three independent experiments (A to C).

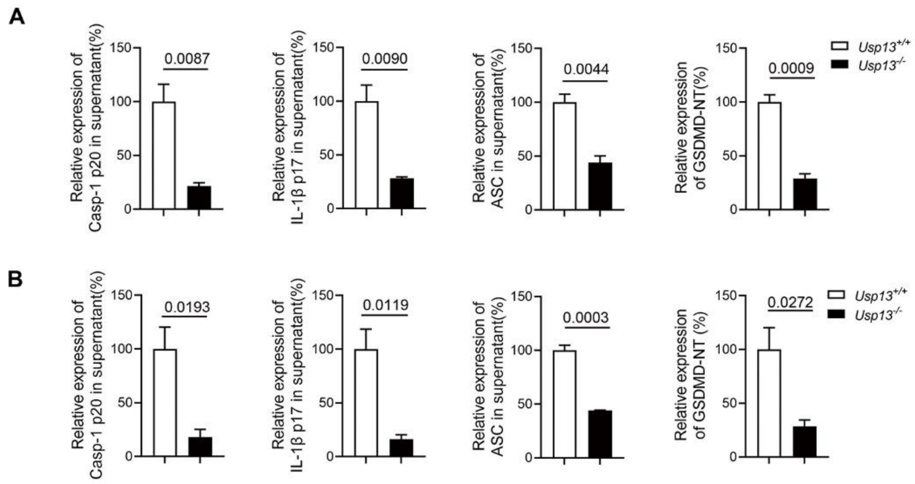

Supplementary Fig. 5. USP13 deficiency inhibits NLRP3 inflammasome activation in BMDMs and PRMs. (**A**) Quantification of relative protein levels as shown in Figure 5F. (**B**) Quantification of relative protein levels as shown in Figure 6C. Data are presented as mean ± SEM from three independent experiments (A and B). Statistical significance was assessed by a two-tailed unpaired *t*-test (A and B)

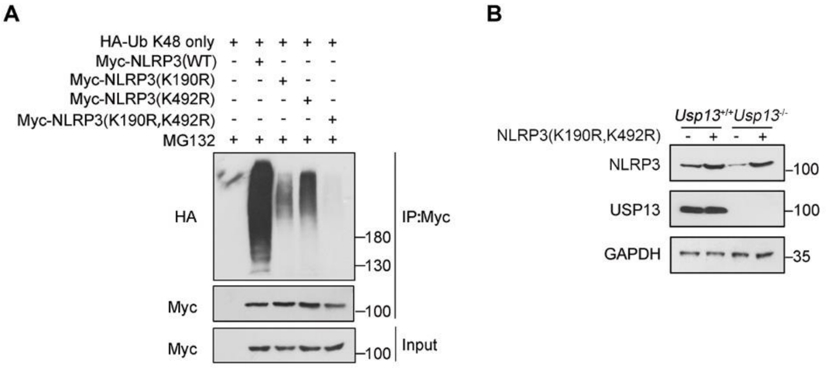

Supplementary Fig. 6. K190 and K492 are the major residues of mouse NLRP3 for K48-linked ubiquitination in BMDMs. (**A**) HEK293T cells were transfected with indicated combinations of plasmids. Before collection, cells were treated with MG132 for 6 h. Immunoblot analysis of NLRP3 ubiquitination in cell lysates immunoprecipitated with anti-c-Myc beads. (**B**) Immunoblot analysis of NLRP3 protein levels in *Usp13*$^{+/+}$ and *Usp13*$^{-/-}$ BMDMs transduced with control or mouse NLRP3 (K190R, K492R) overexpression lentiviruses. Data are representative of three independent experiments (A and B).

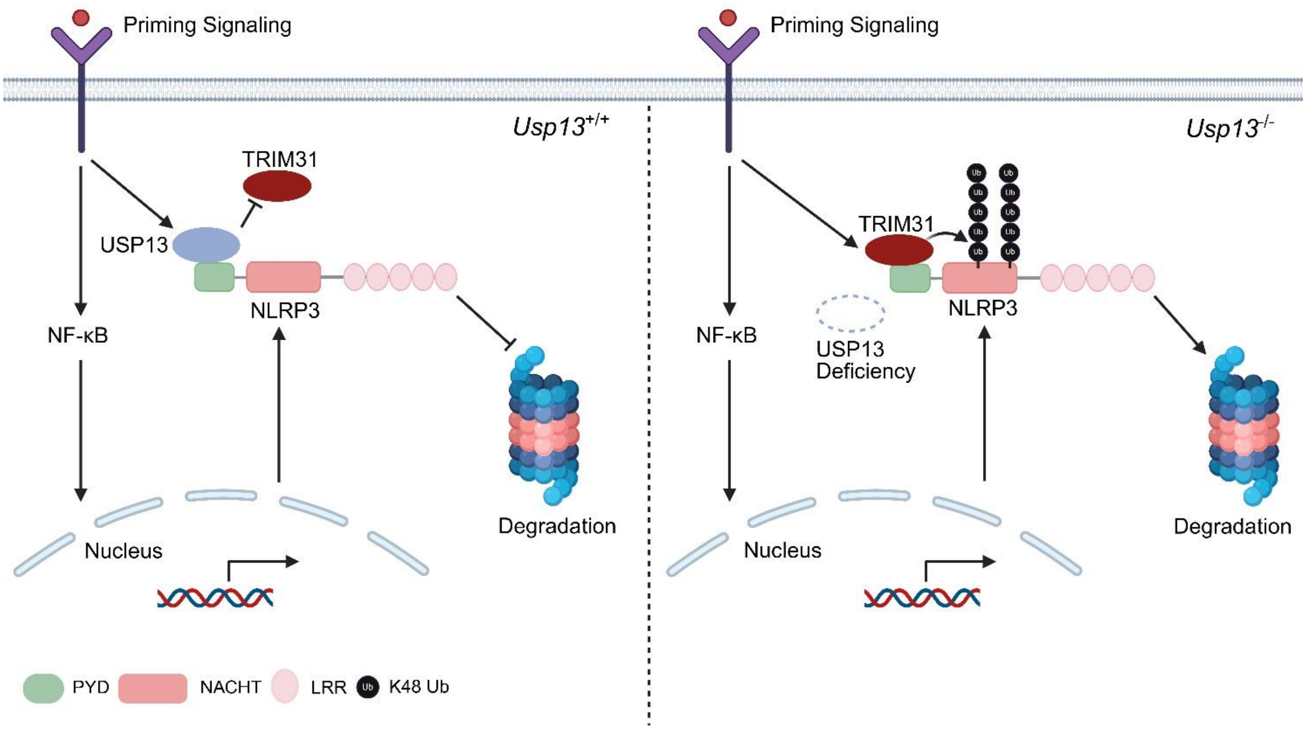

Supplementary Fig. 7. Schematic diagram of the mechanism by which USP13 stabilizes NLRP3. USP13 competes with TRIM31 for binding to the PYD domain of NLRP3 protein, thereby preventing TRIM31-mediated K48-linked polyubiquitination of NLRP3 on K192 and K496 residues, which ultimately inhibits NLRP3 proteasomal degradation.

Supplementary Table S1. Identification of NLRP3-binding proteins by IP-MS. Screening of NLRP3-binding proteins in Flag-tagged human NLRP3 overexpressed THP-1 cells by IP-MS.

Data S1. Raw data for all figures.

Data S2. Uncropped western blots for all figures.

Fig.1B

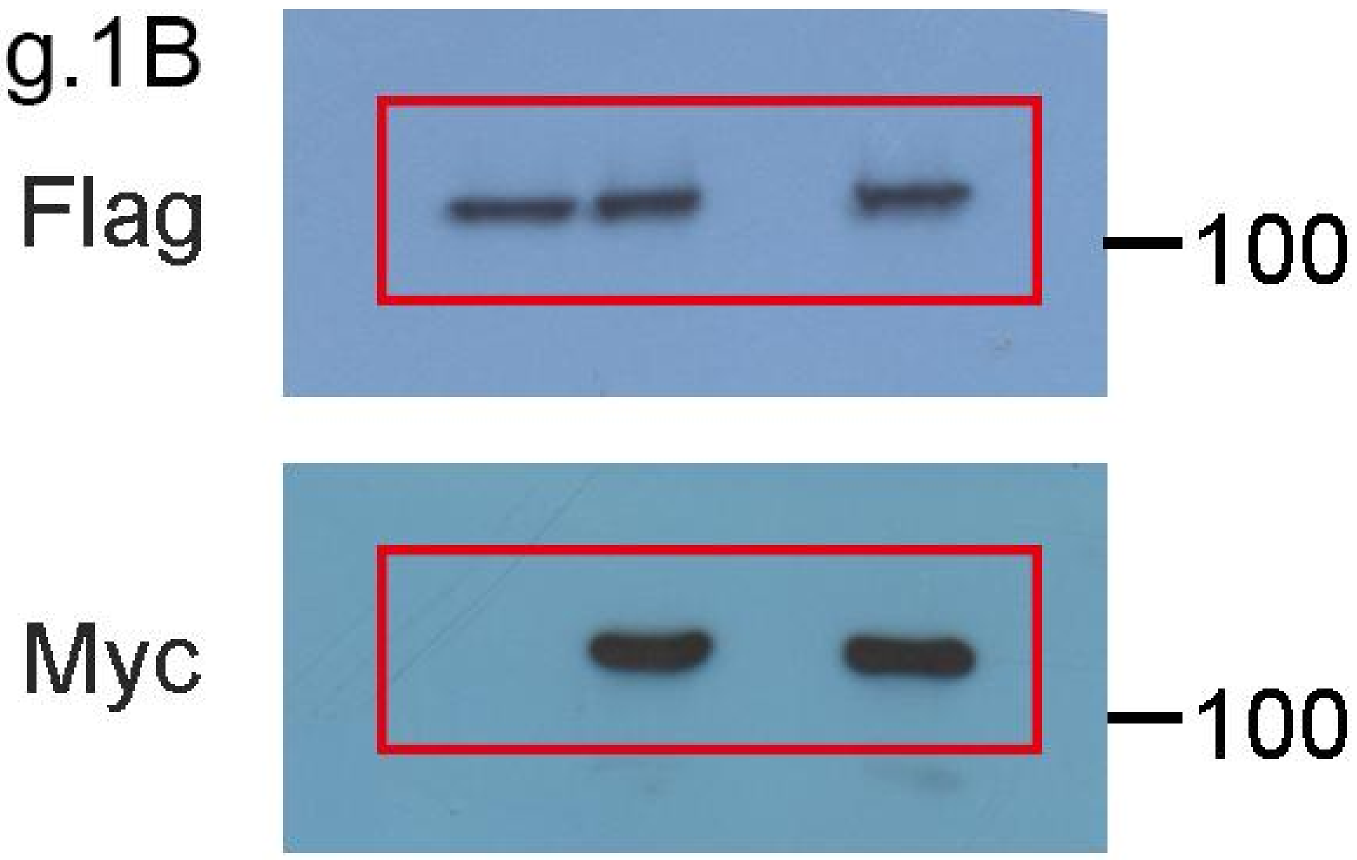

Fig.1C

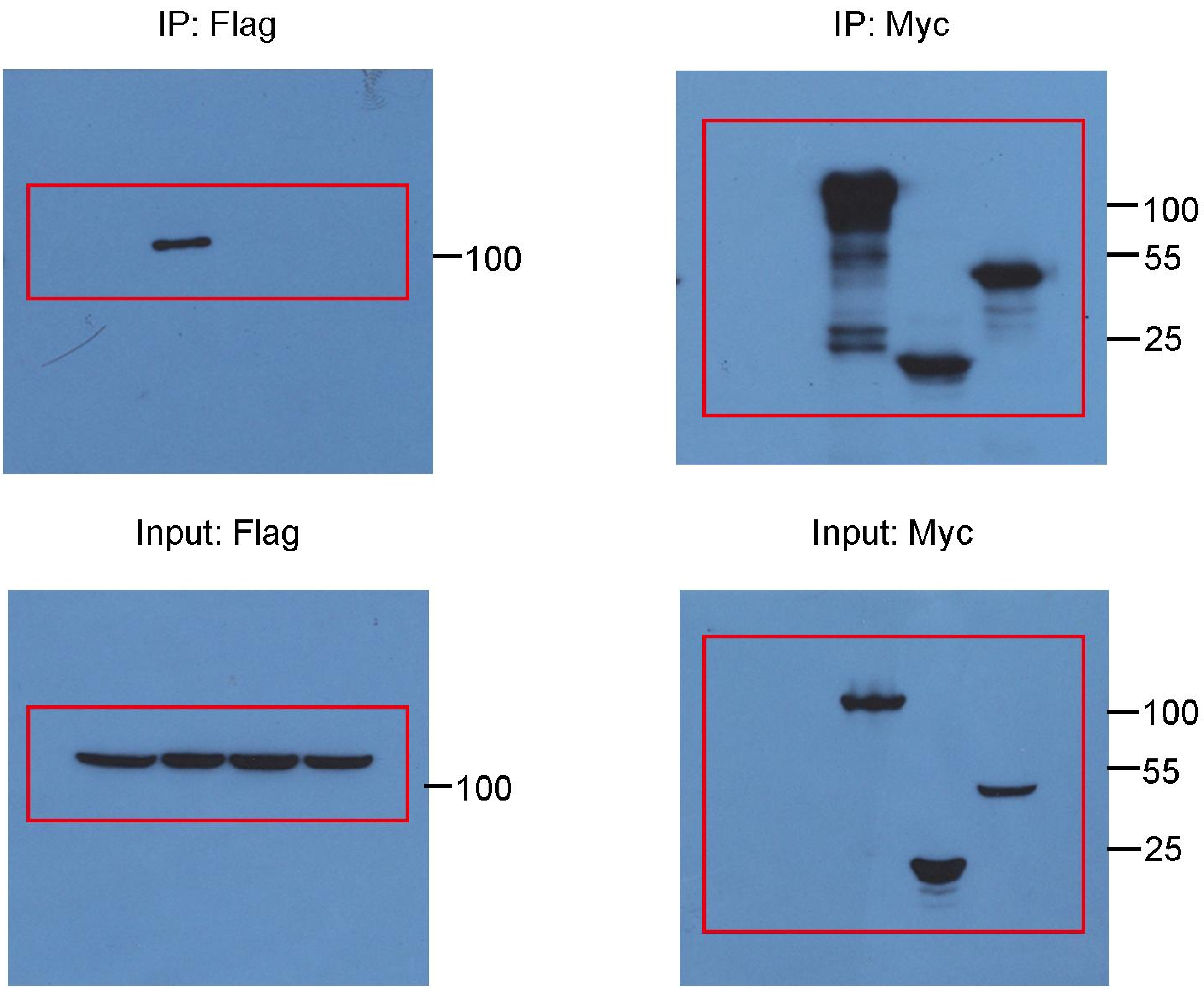

Fig.1D

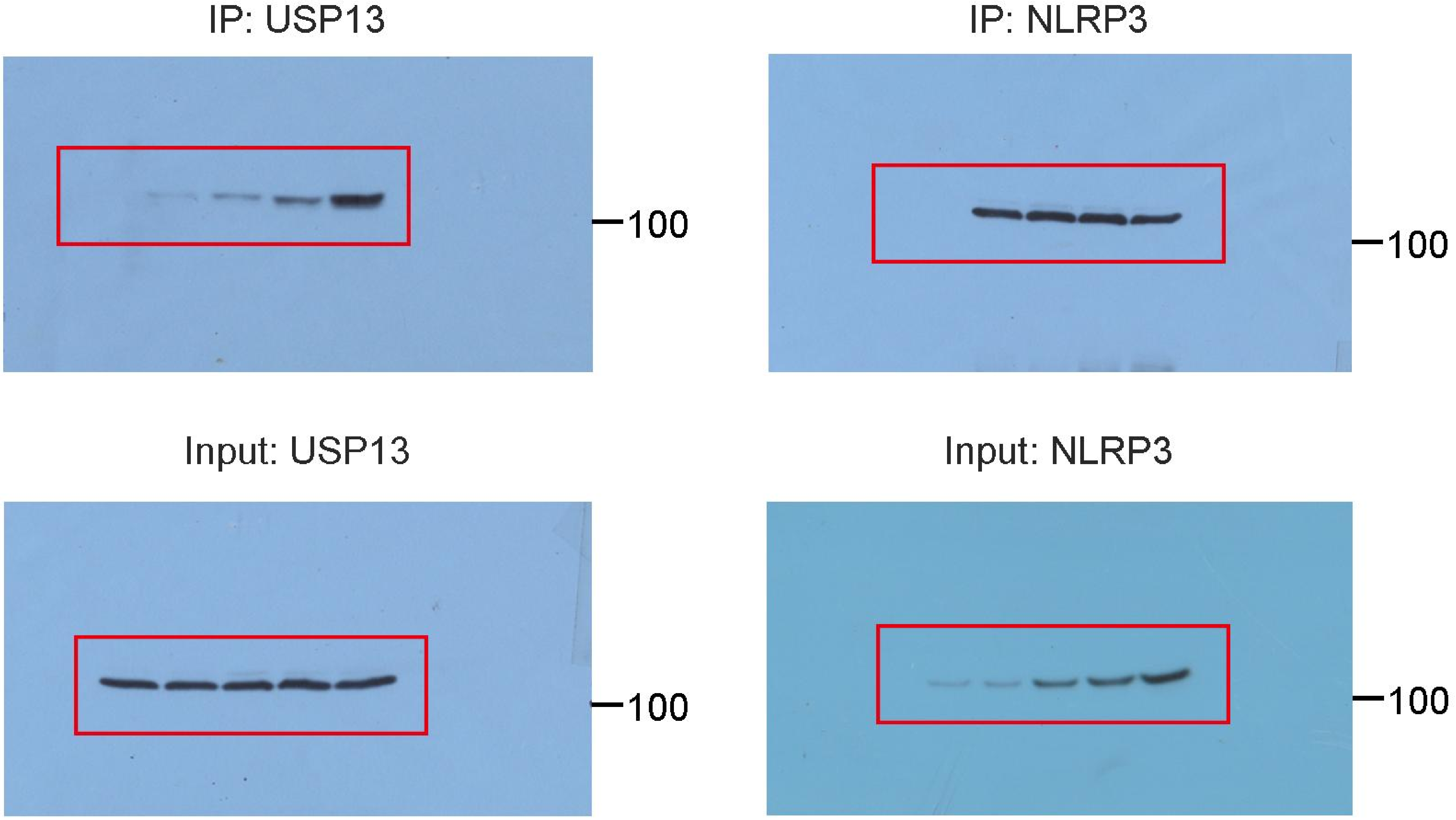

Fig.1E

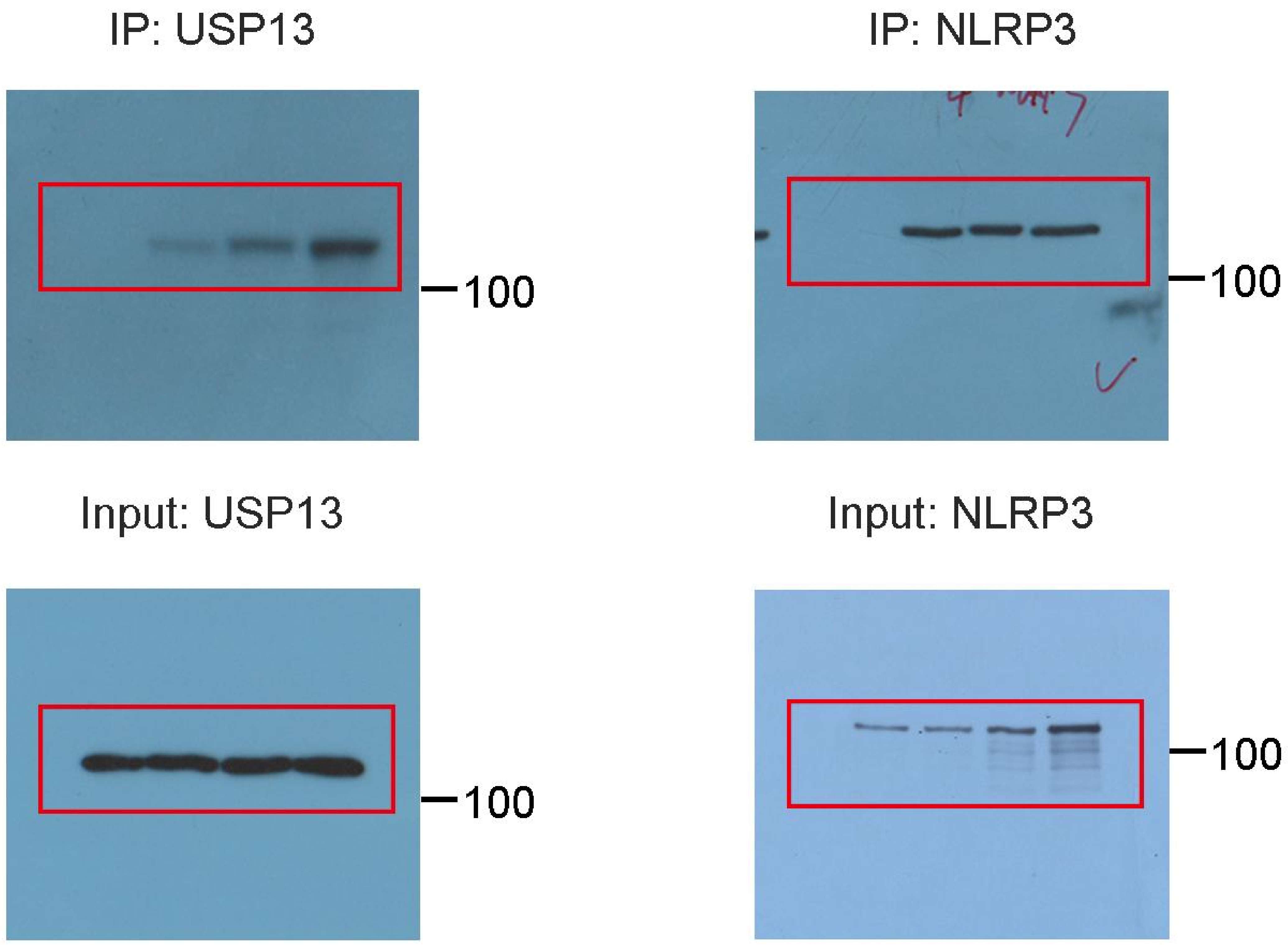

Fig.1F

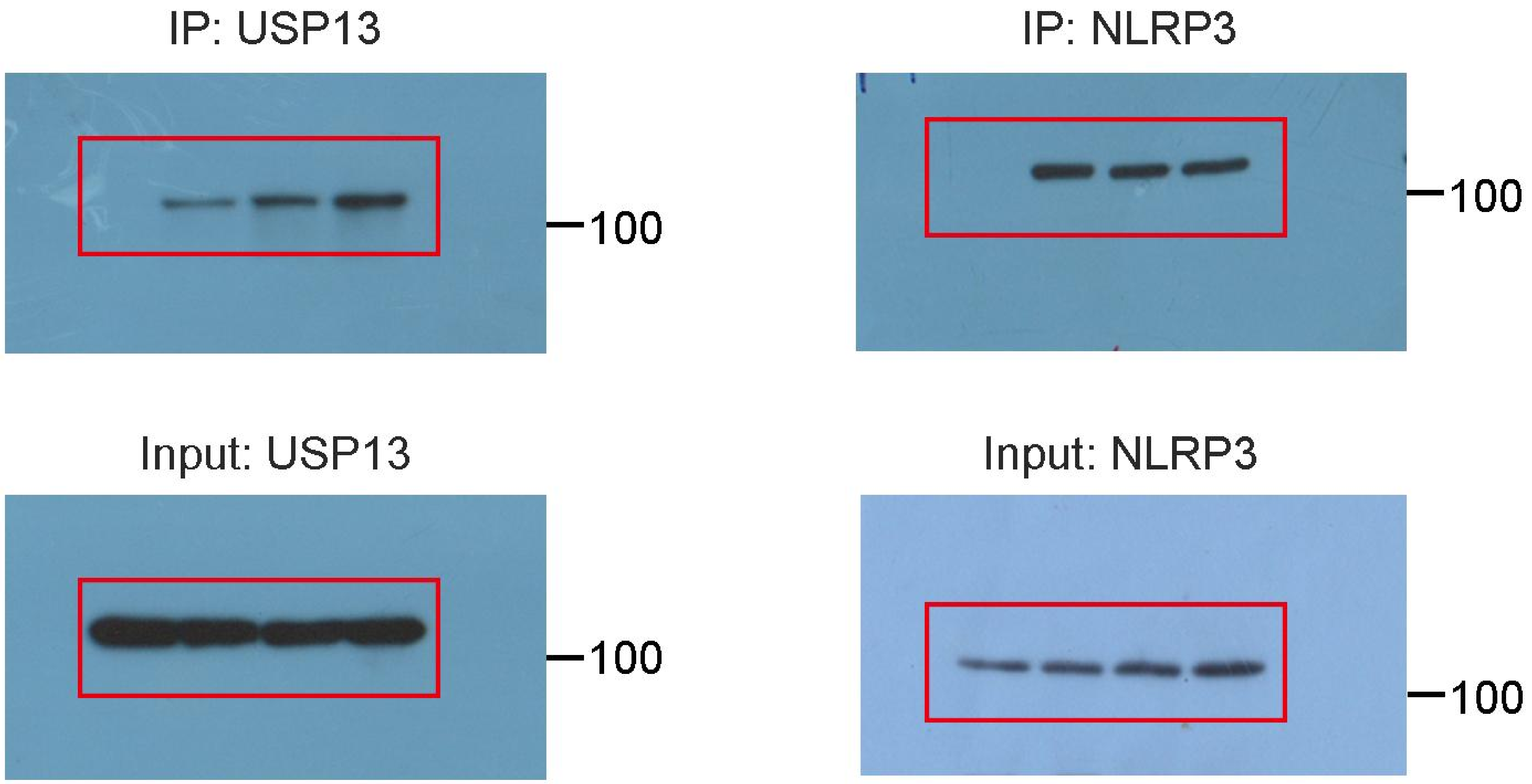

Fig.1G

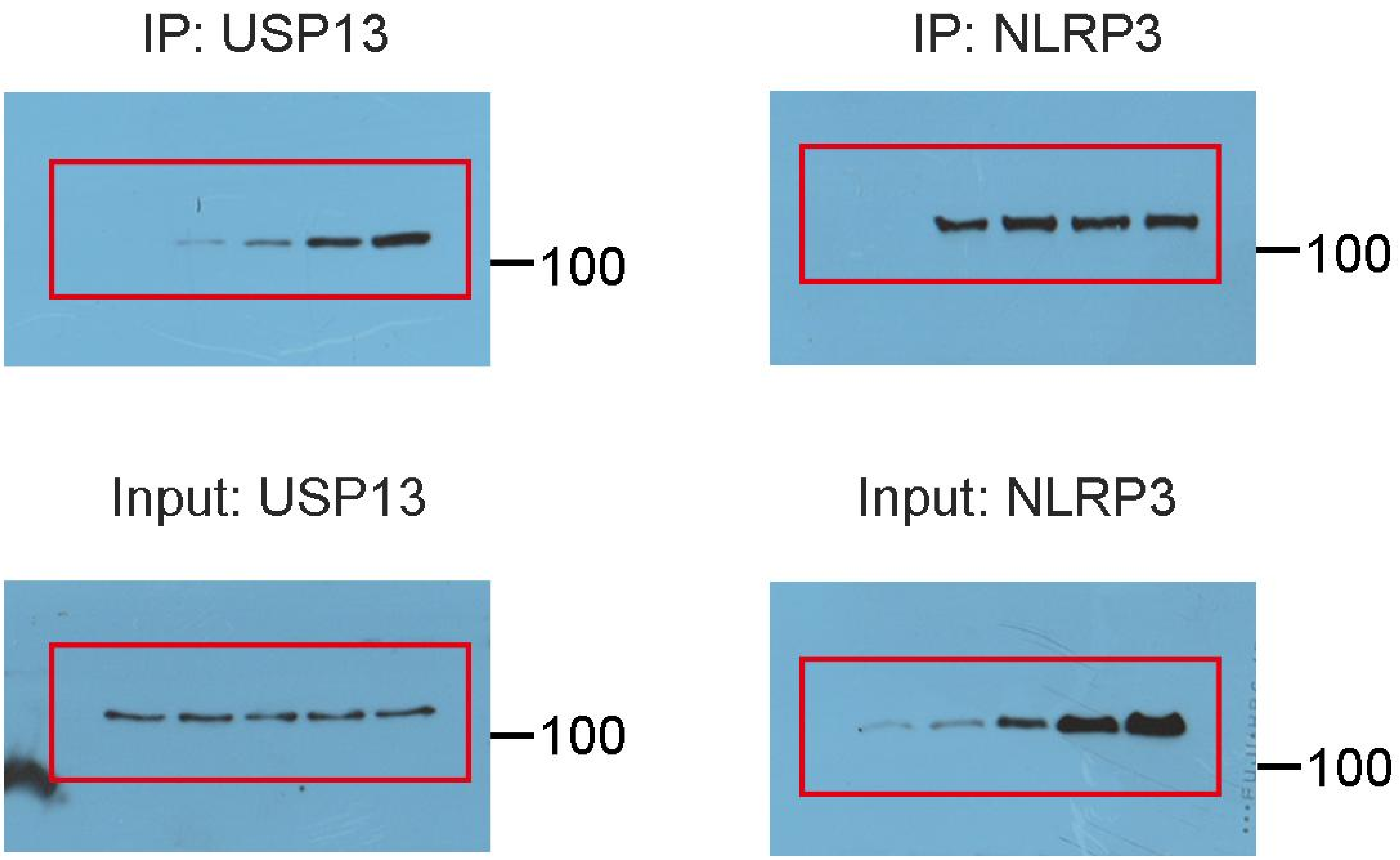

Fig.1l

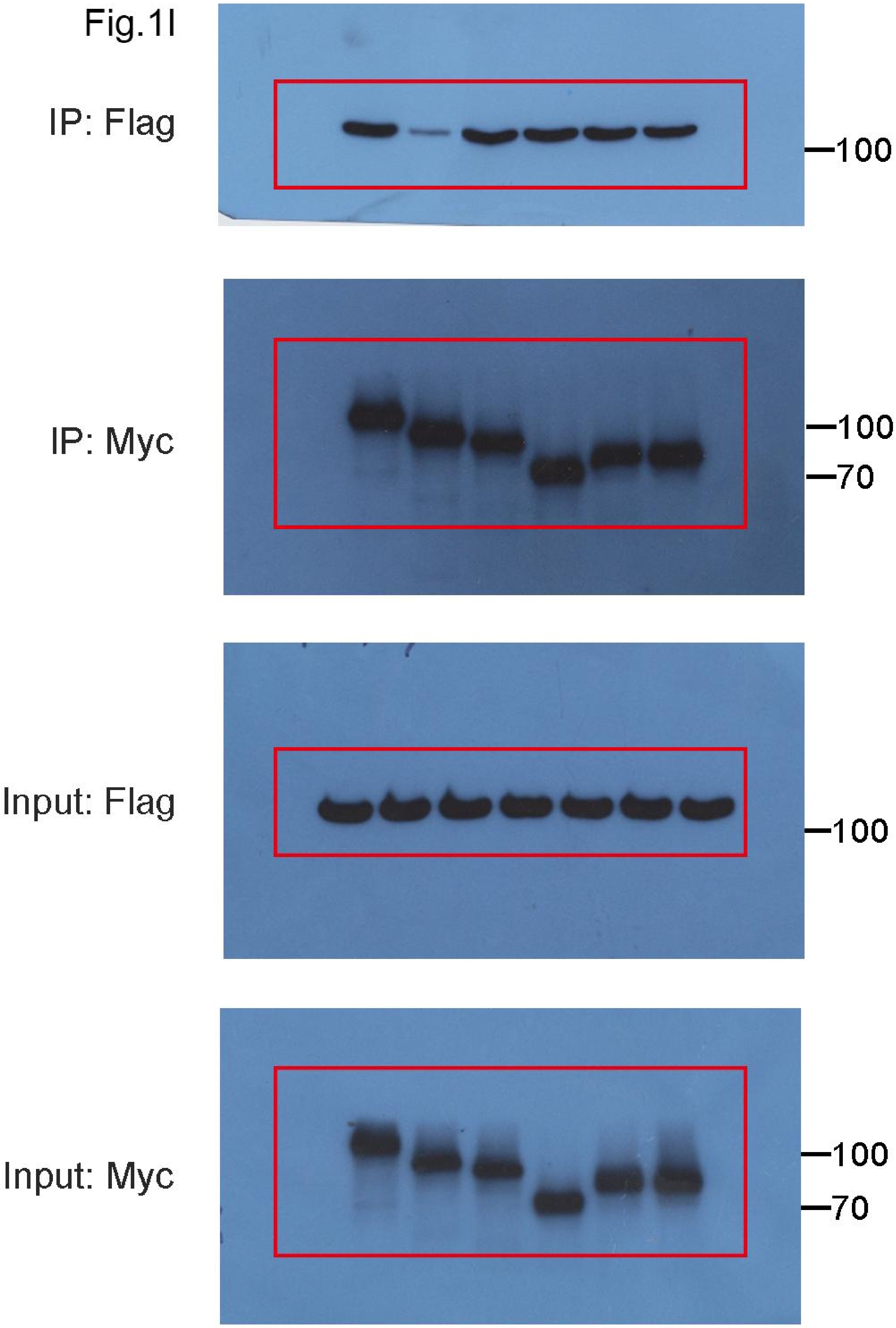

Fig.1J

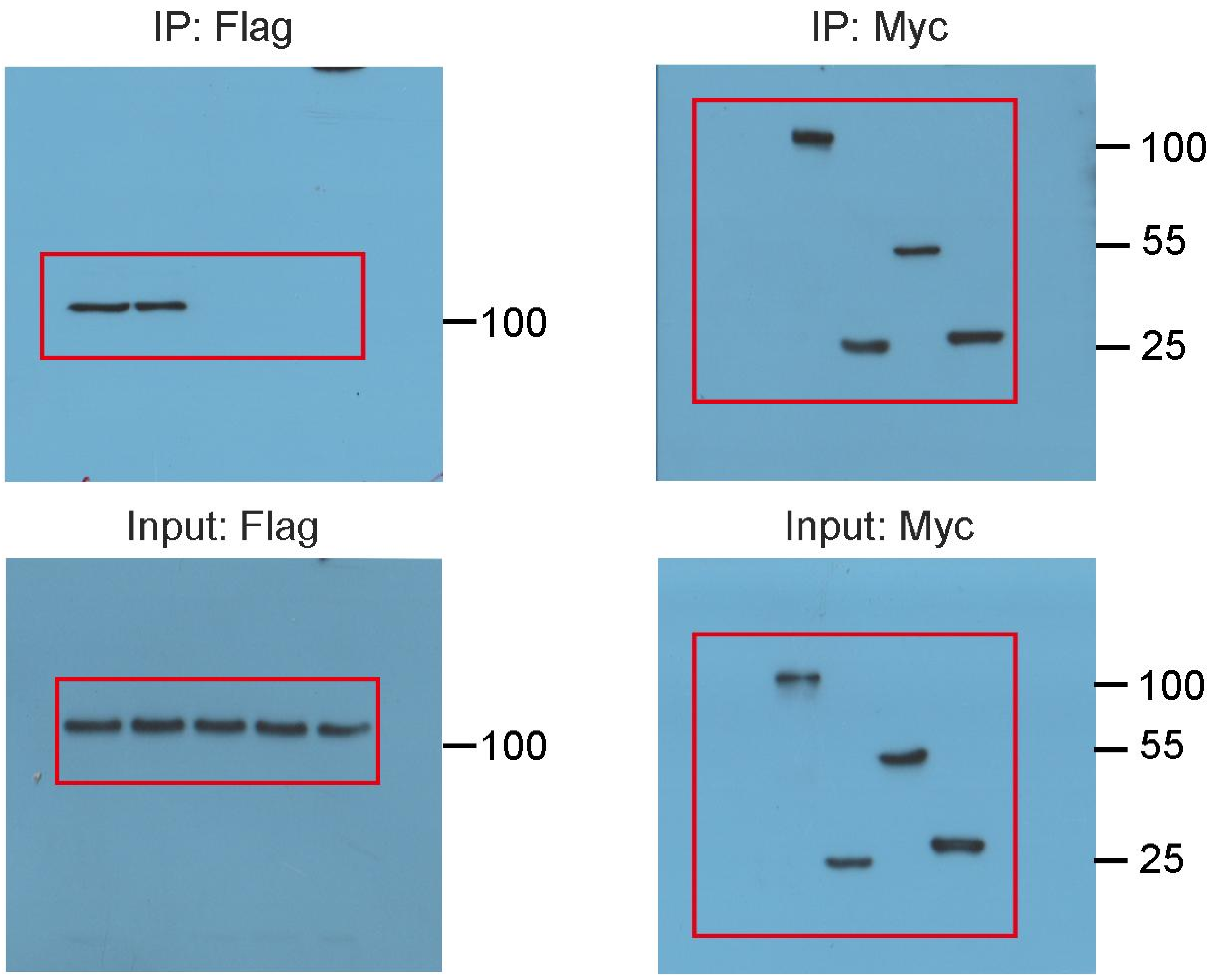

Fig.1L

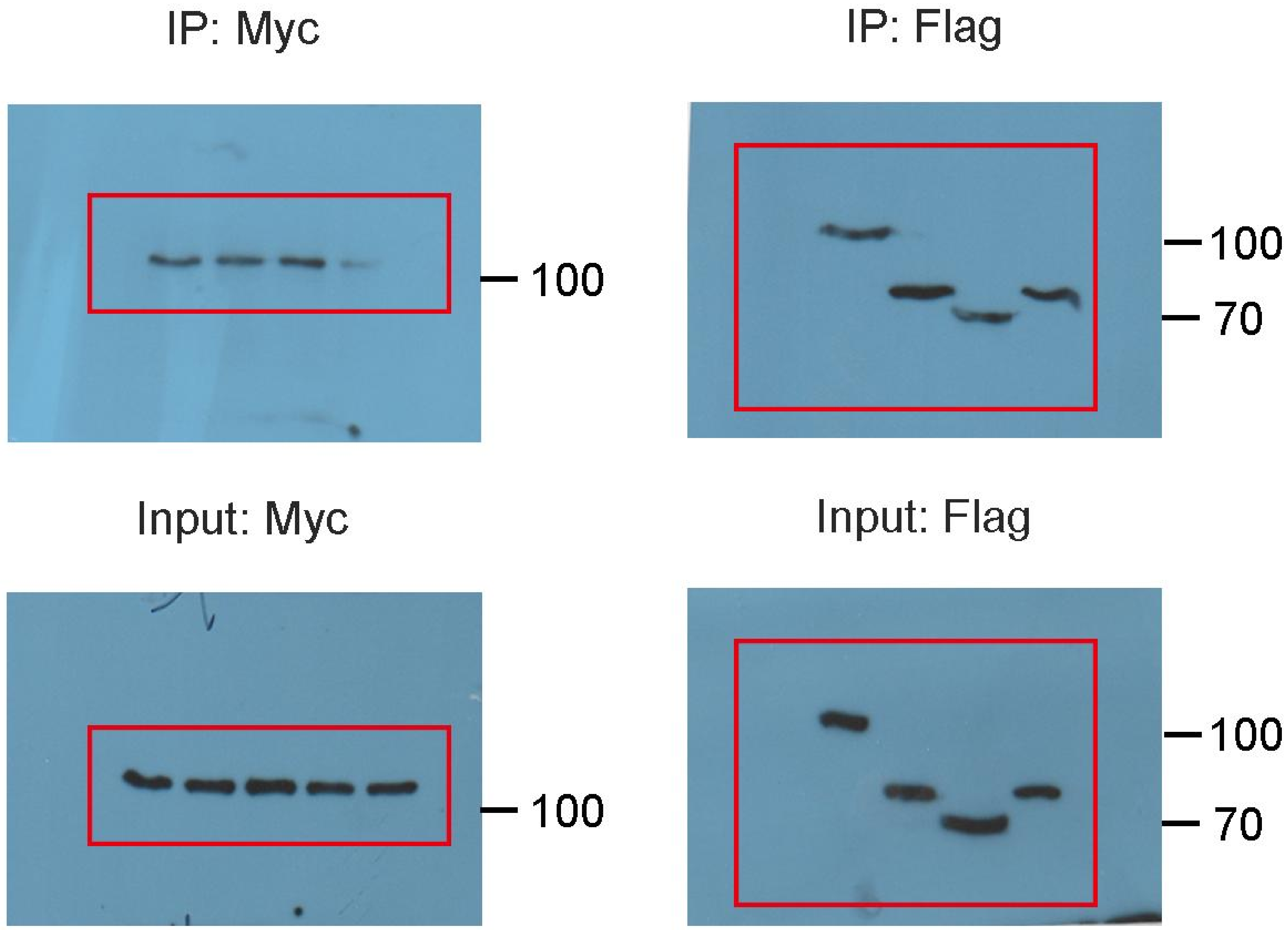

Fig.2A

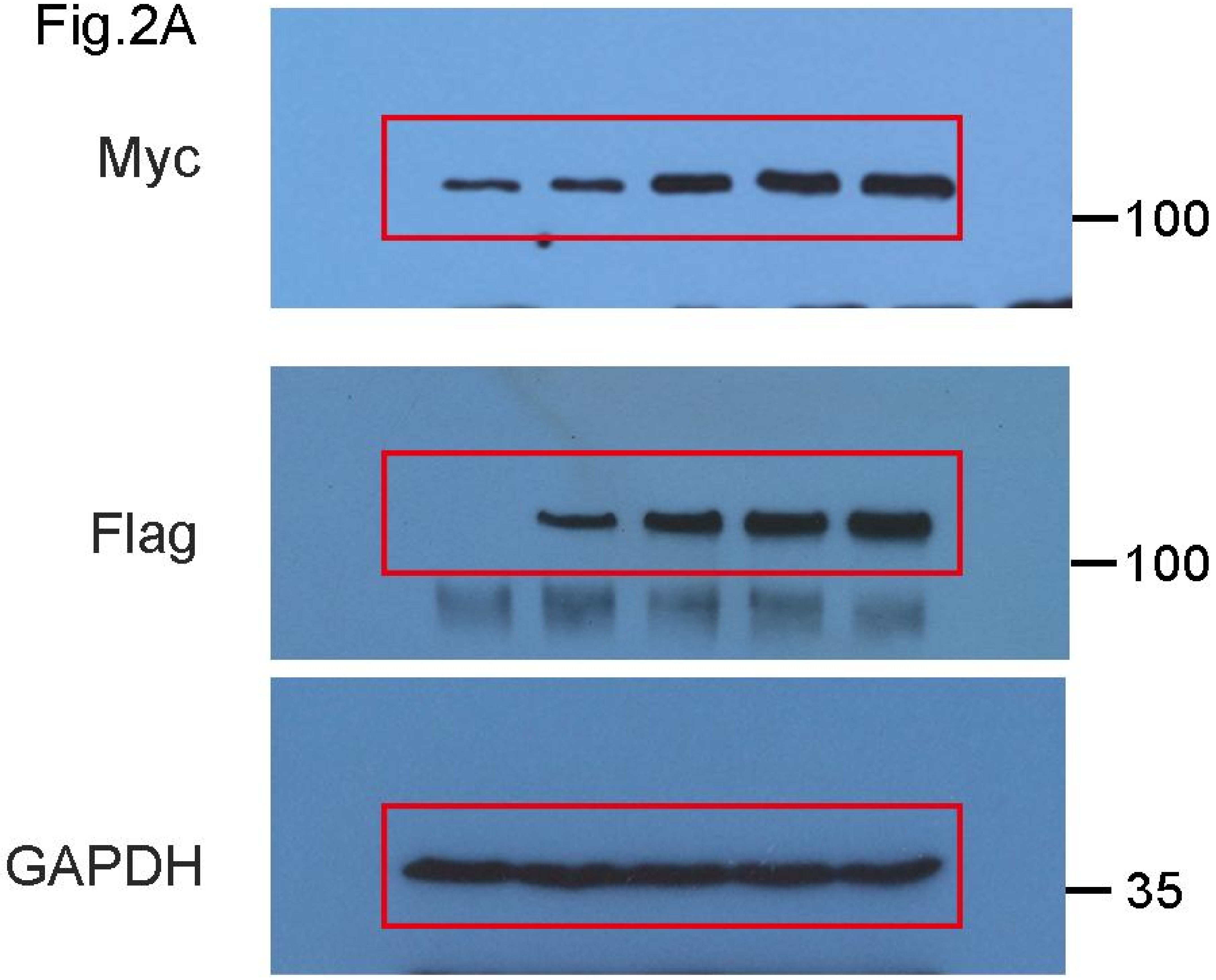

Fig.2B

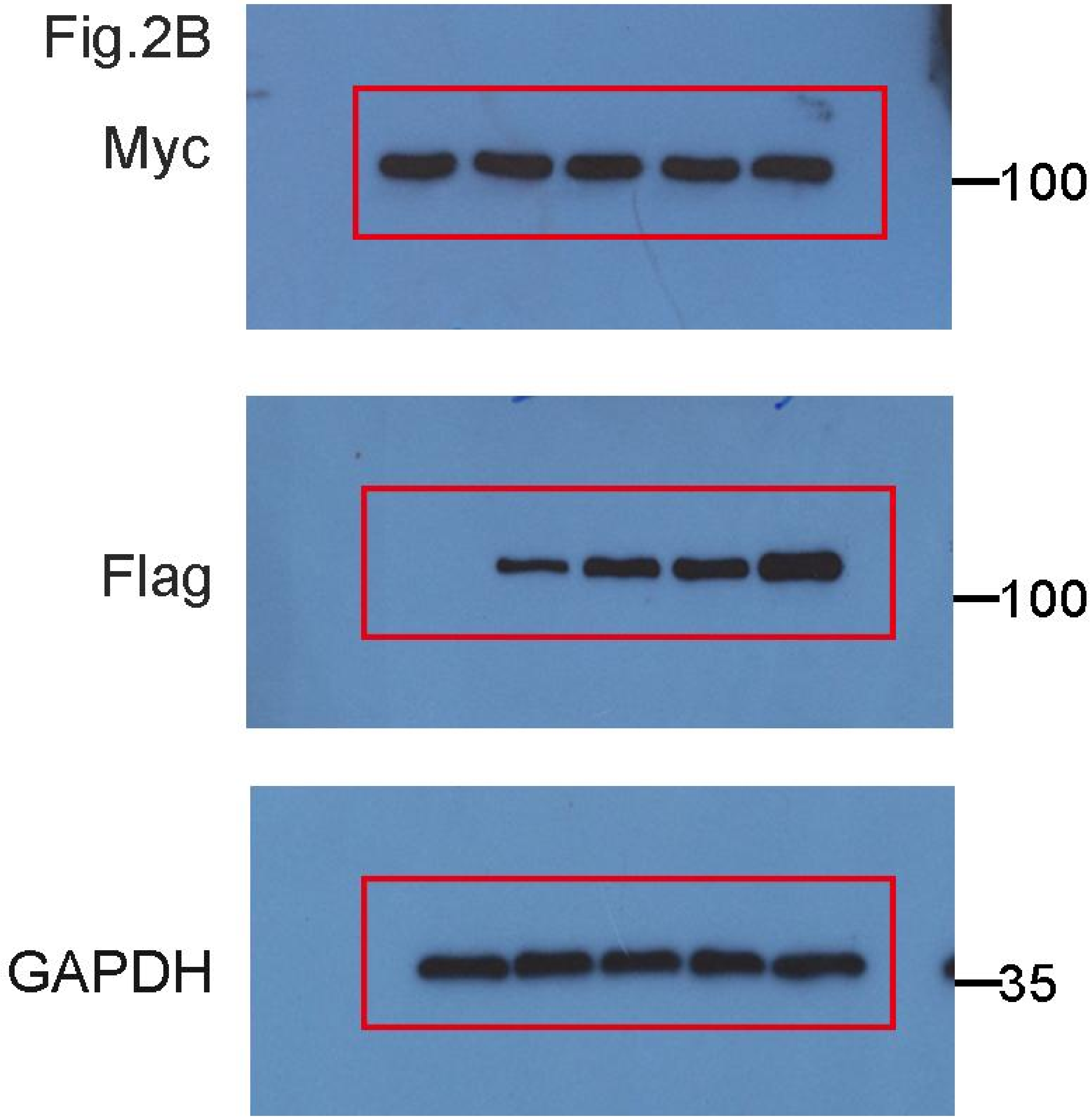

Fig.2C

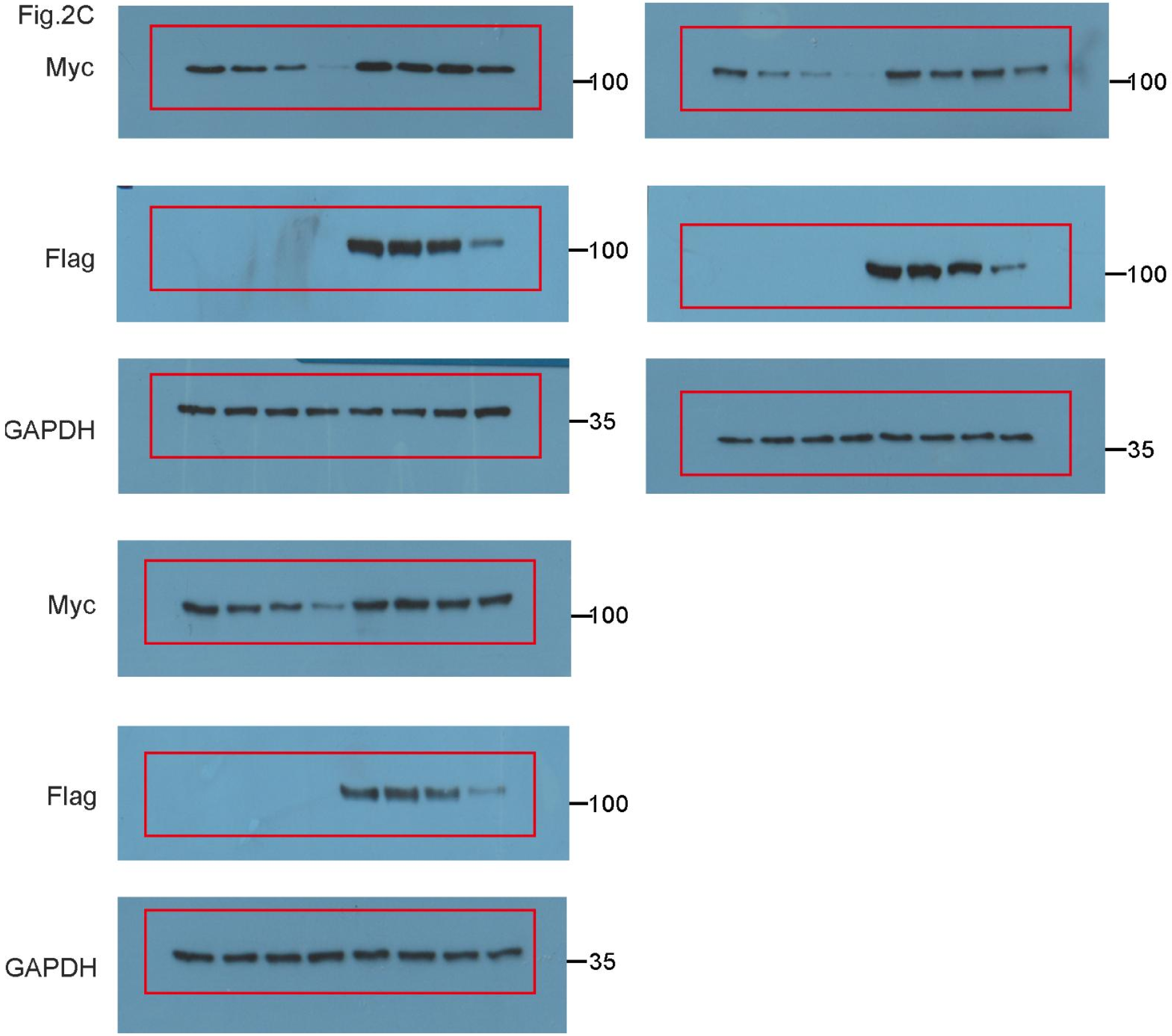

Fig.2D

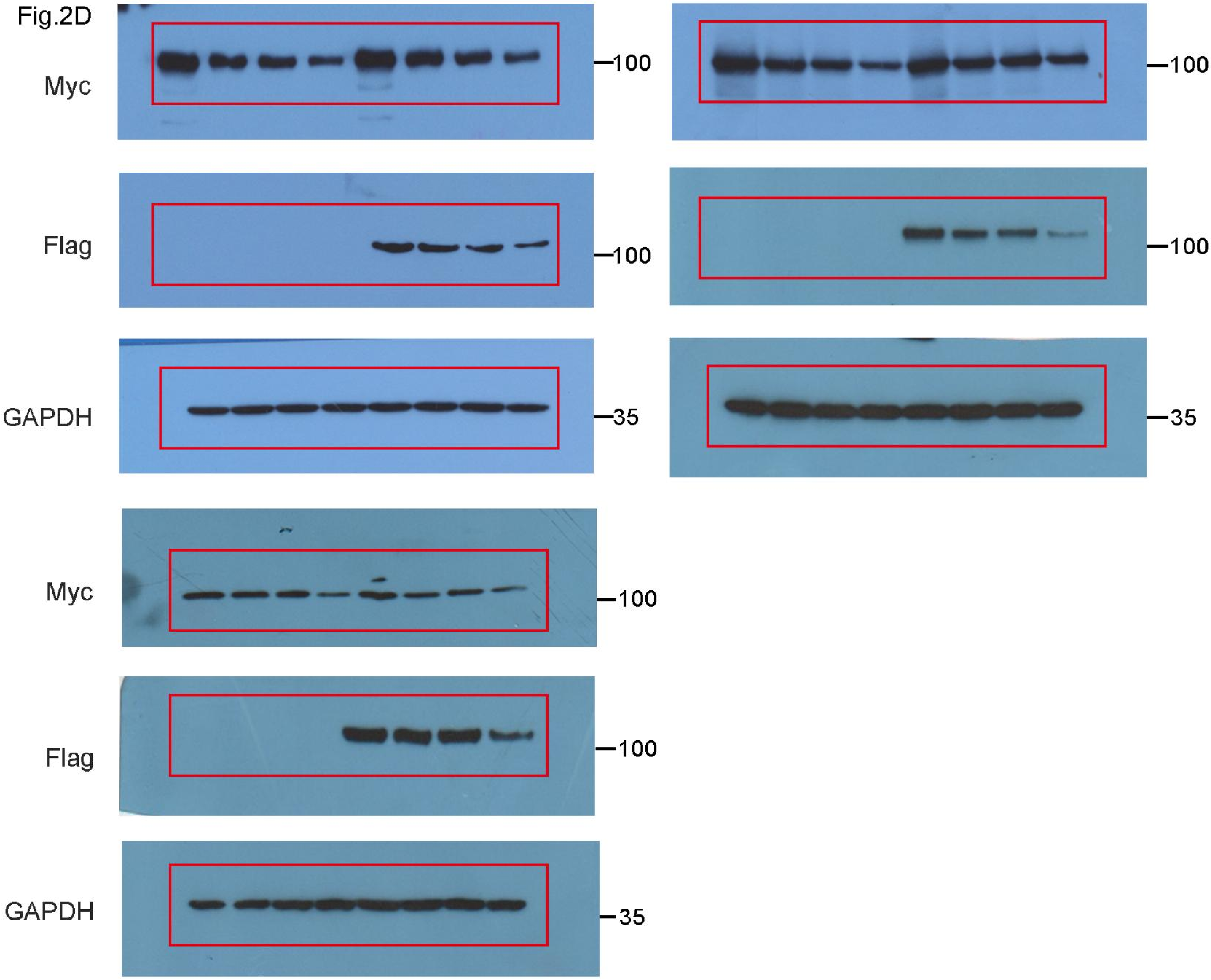

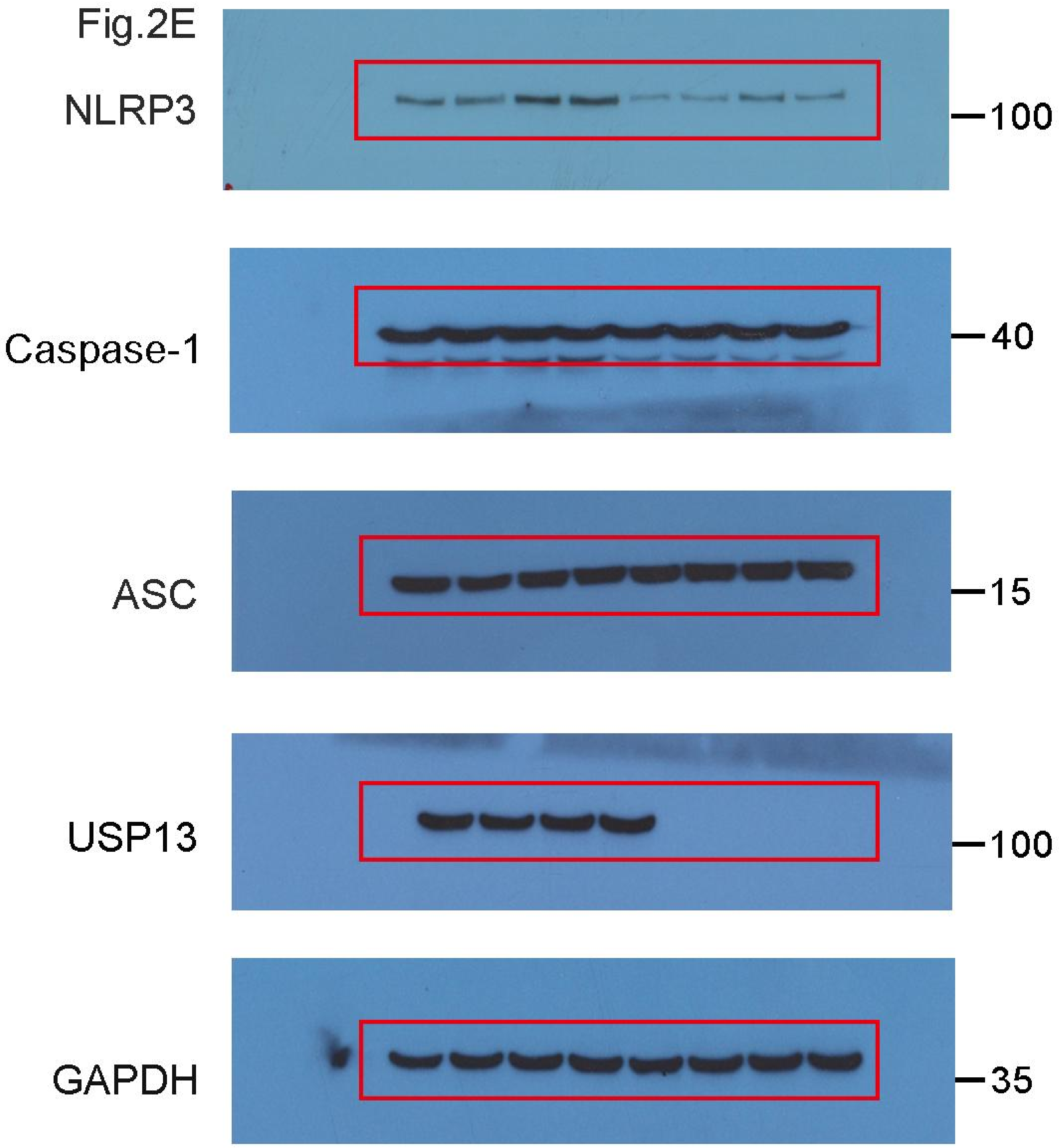

Fig.2E
NLRP3
100
Caspase-1
40
ASC
15
USP13
100
GAPDH
35

Fig.2F

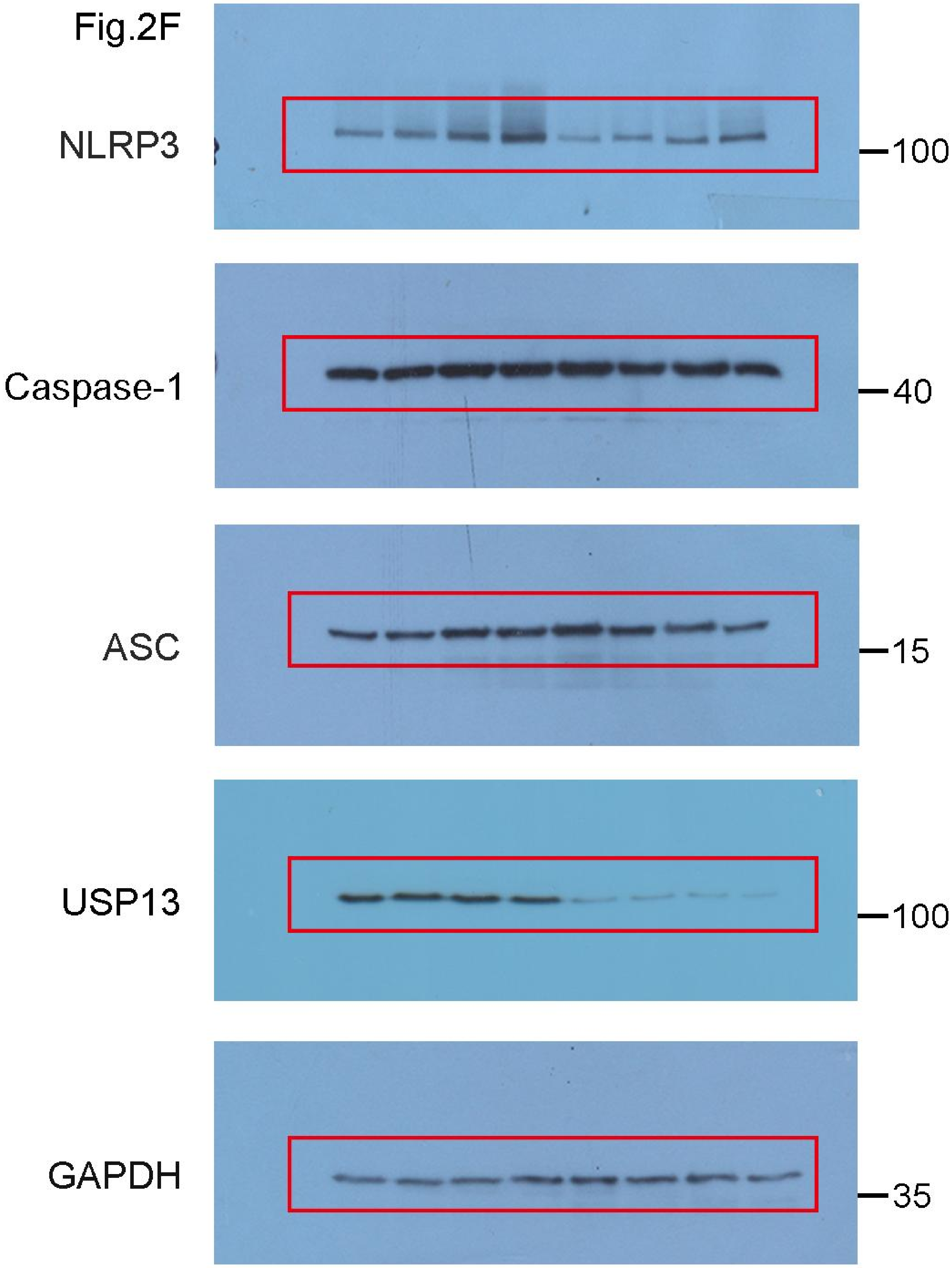

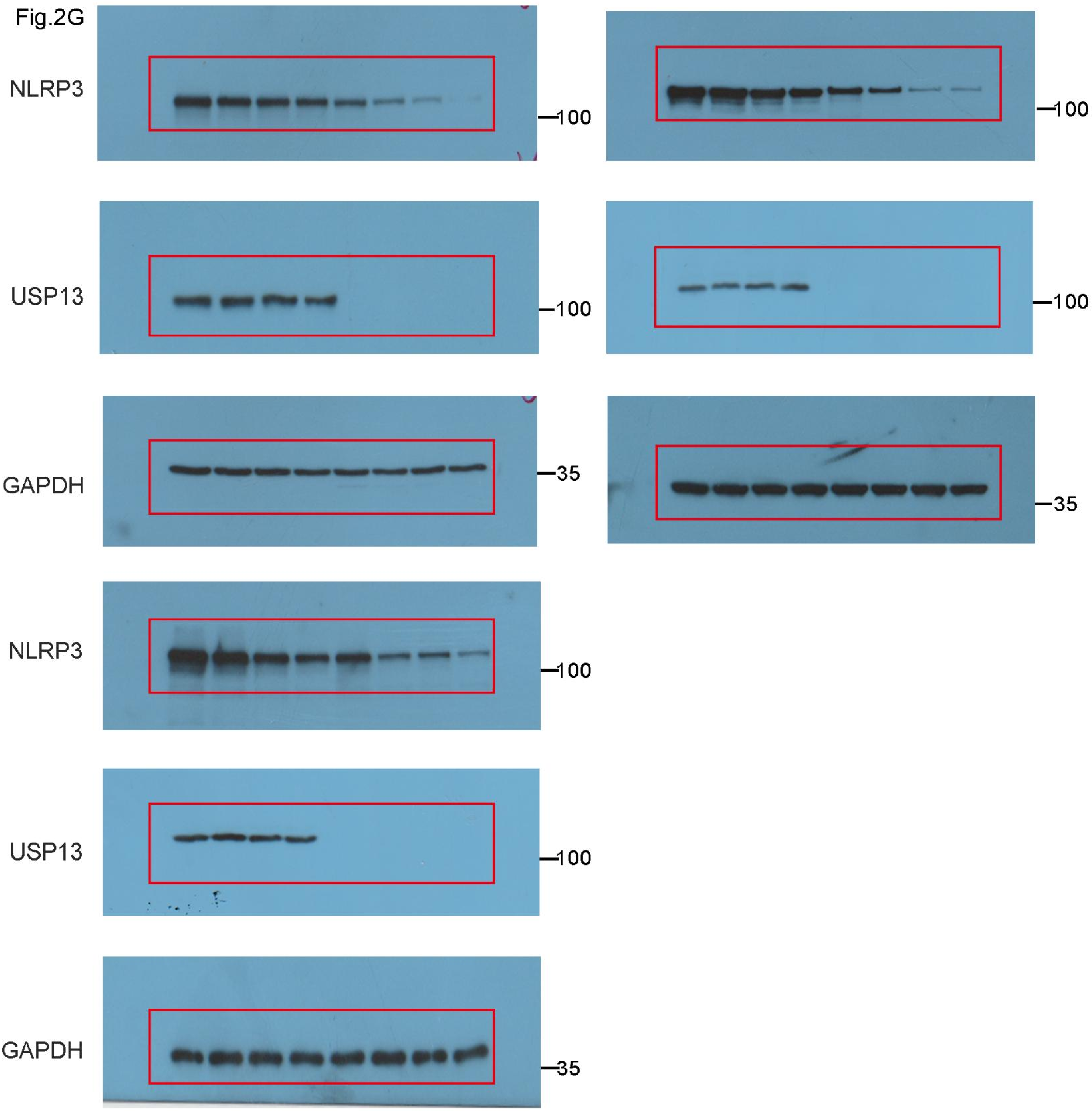
Fig.2G
NLRP3
USP13
GAPDH
NLRP3
USP13
GAPDH
100
35

Fig.2H

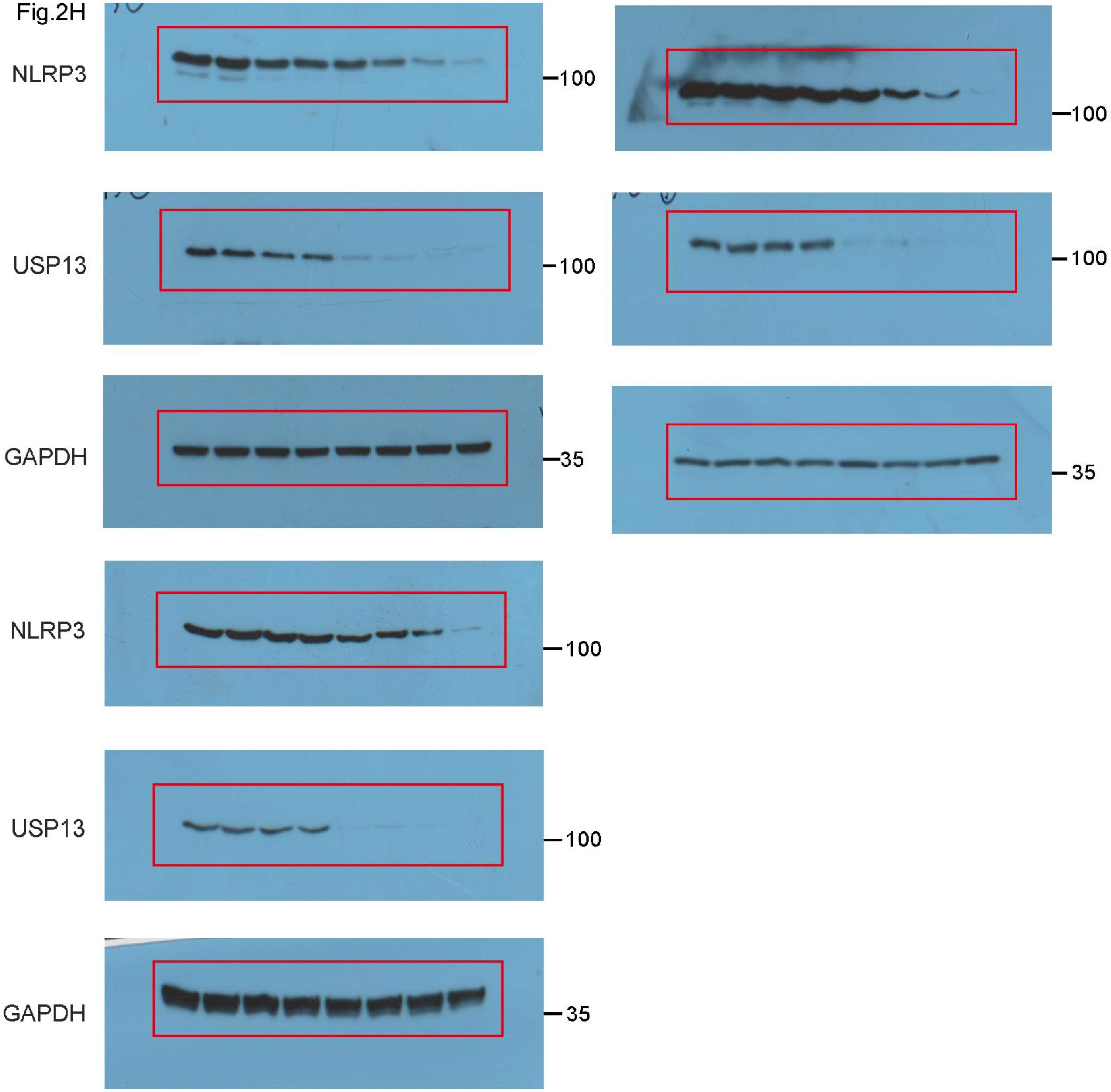
NLRP3
USP13
GAPDH
NLRP3
USP13
GAPDH
100
100
35
100
100
35
100
100
35

Fig.3A

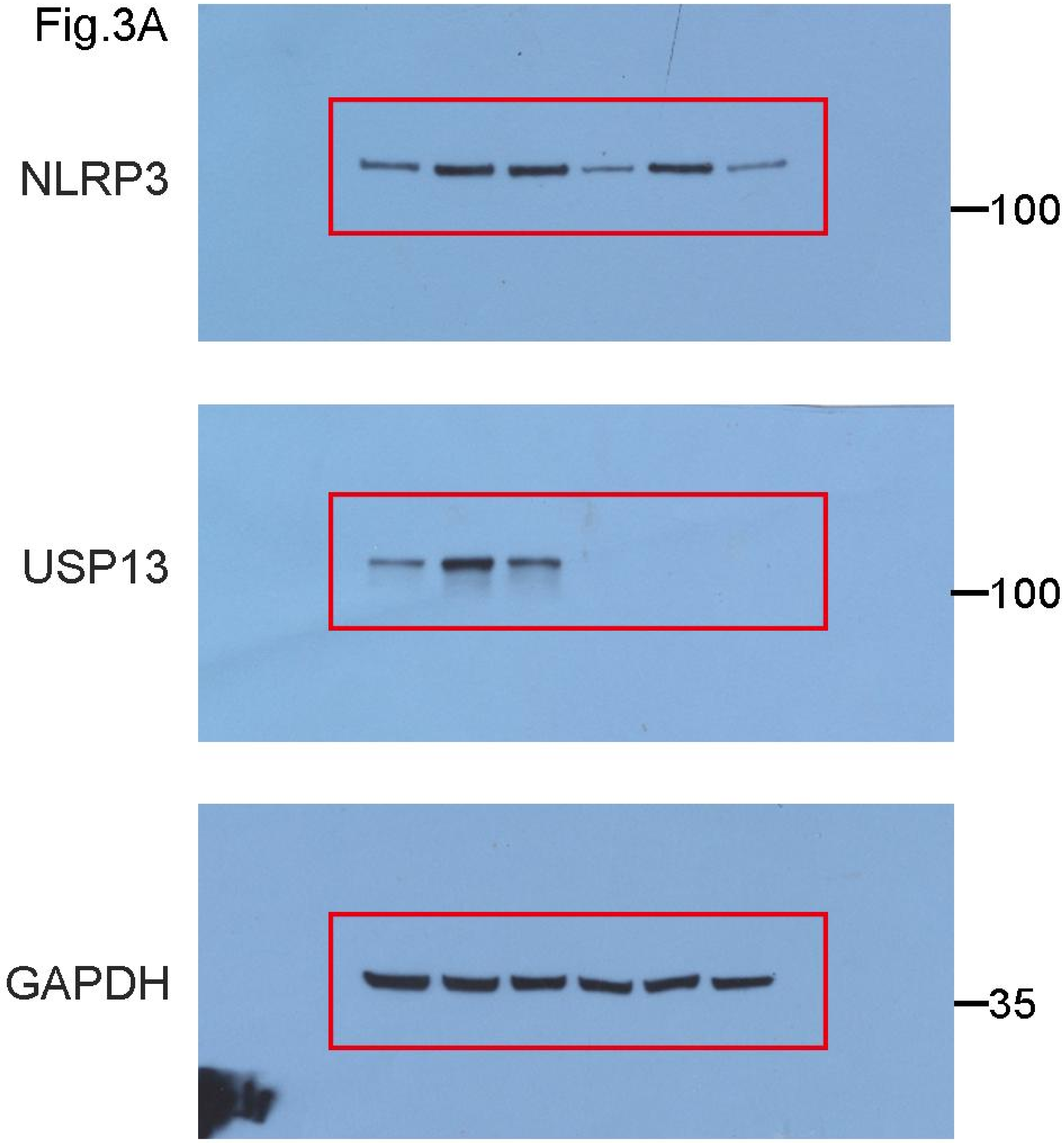

Fig.3B

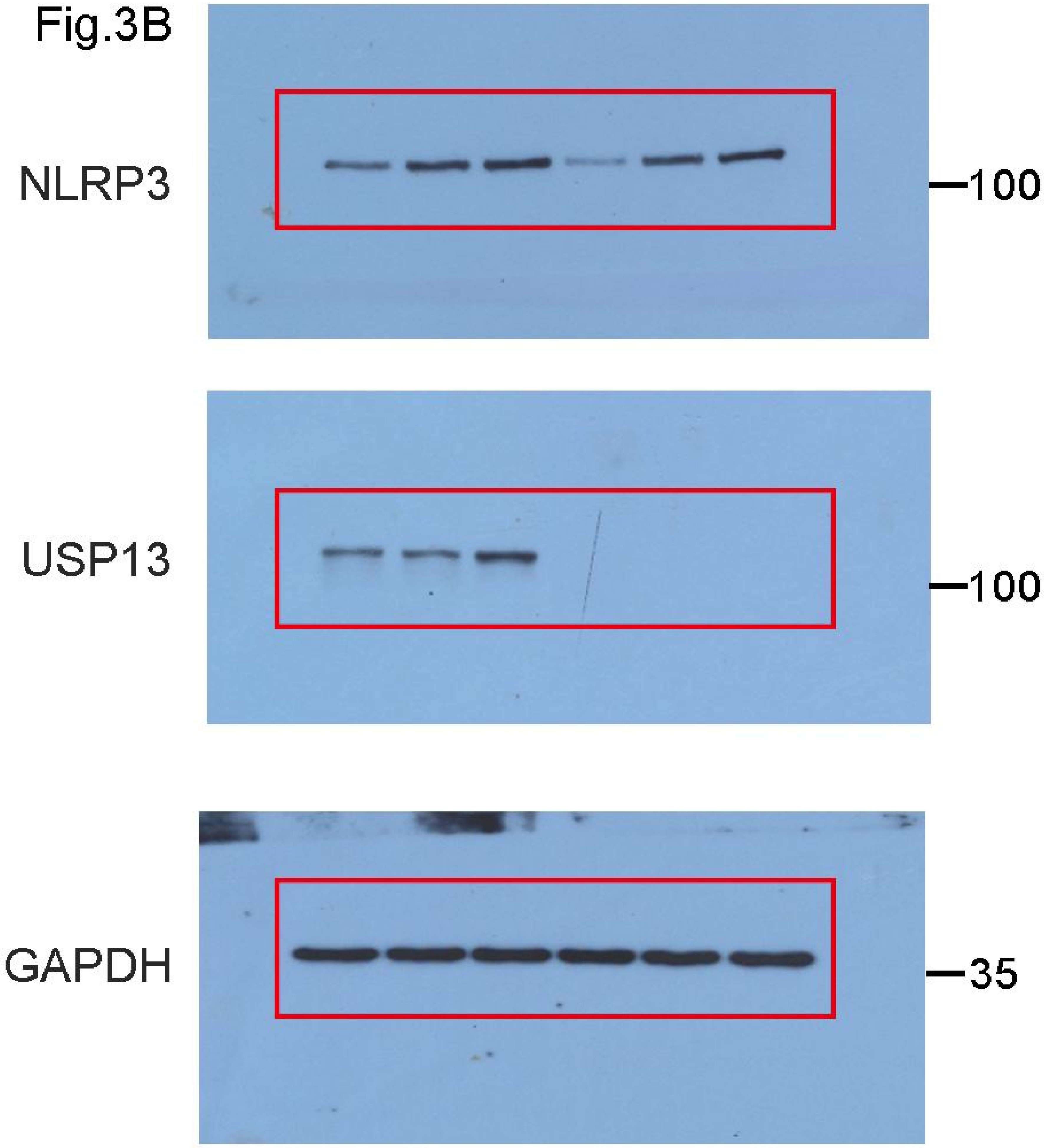

Fig.3C

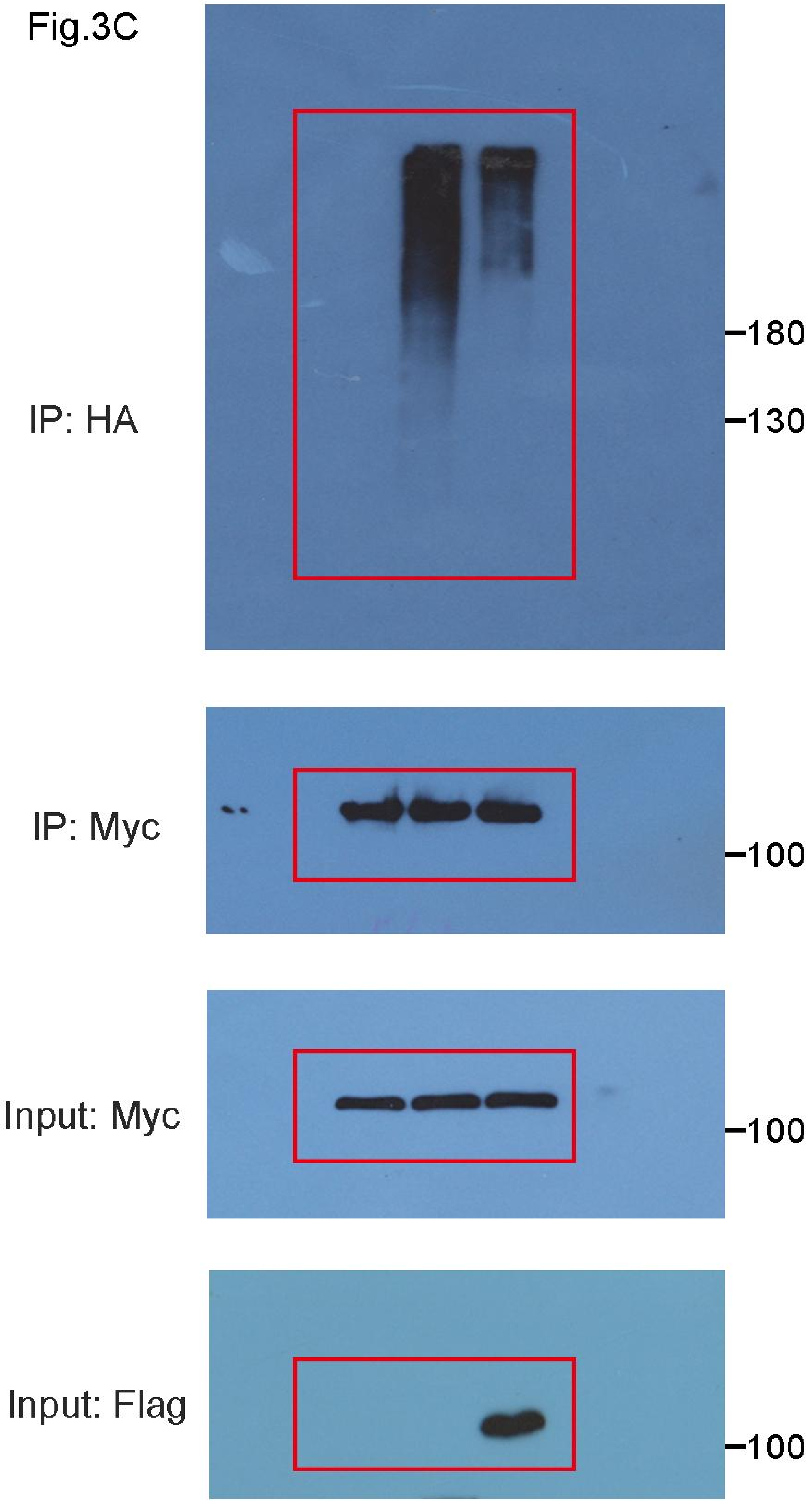

Fig.3D

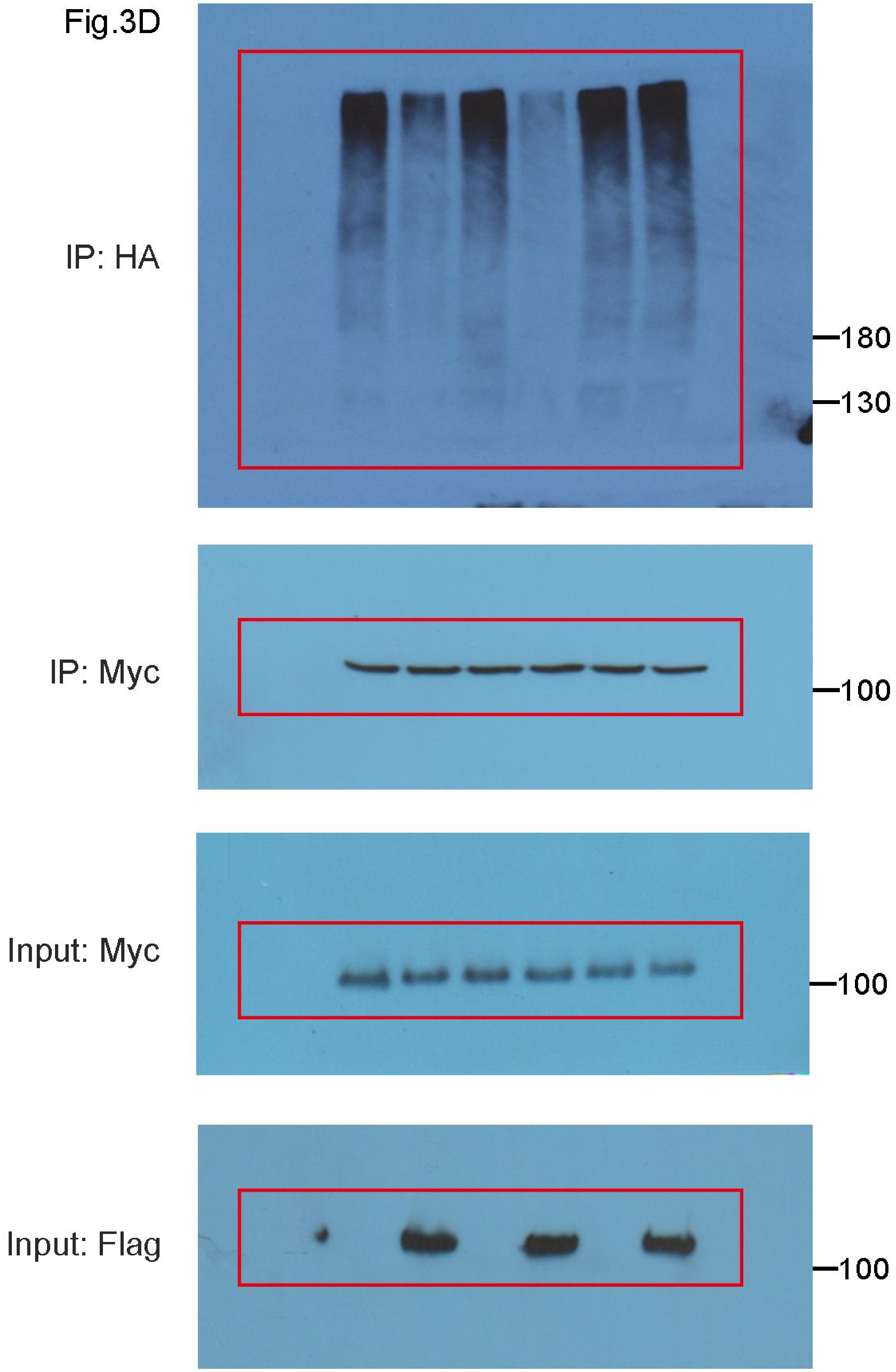

Fig.3E

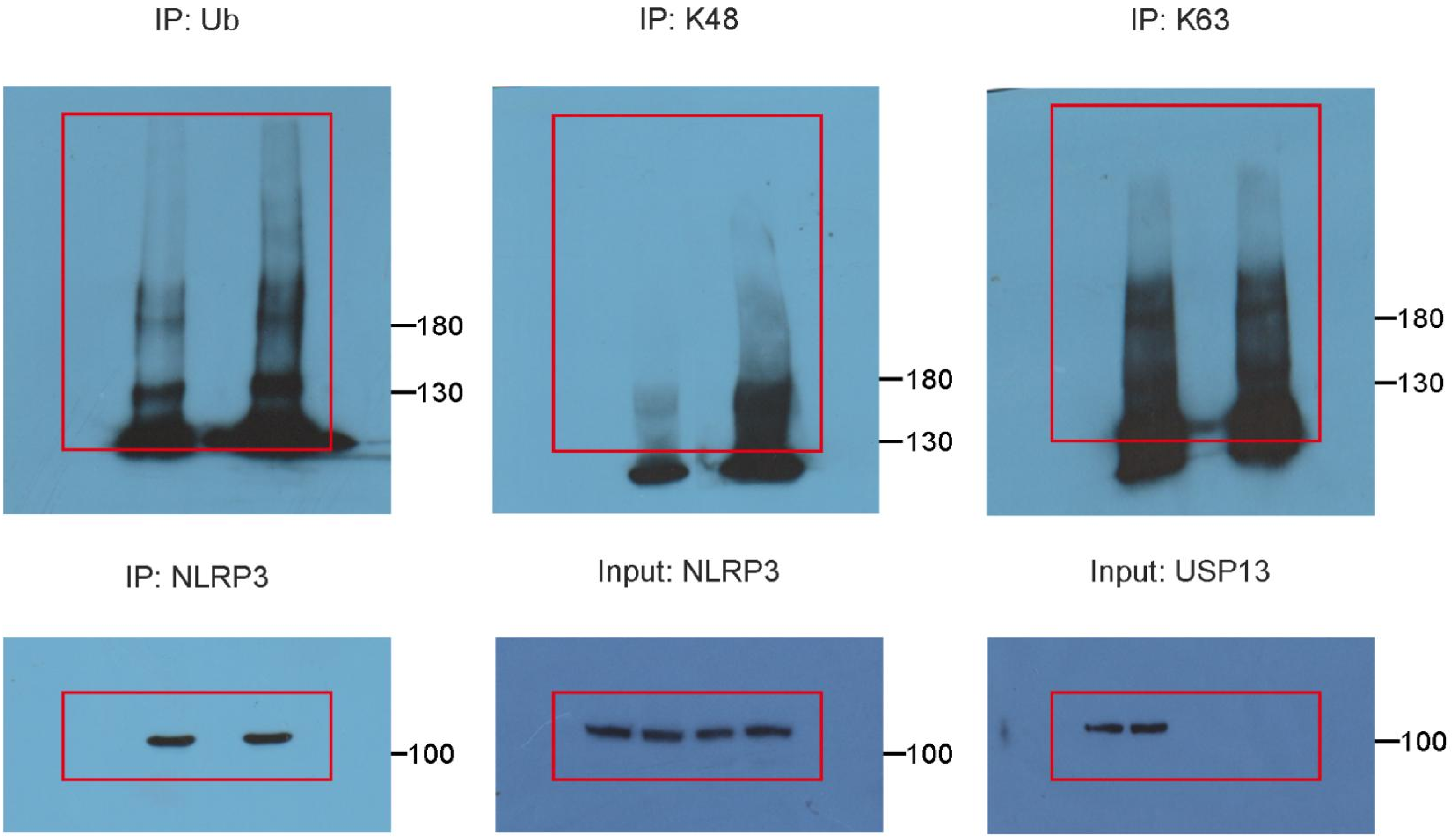

IP: Ub
IP: K48
IP: K63
180
130
180
130
180
130
IP: NLRP3
Input: NLRP3
Input: USP13
100
100
100

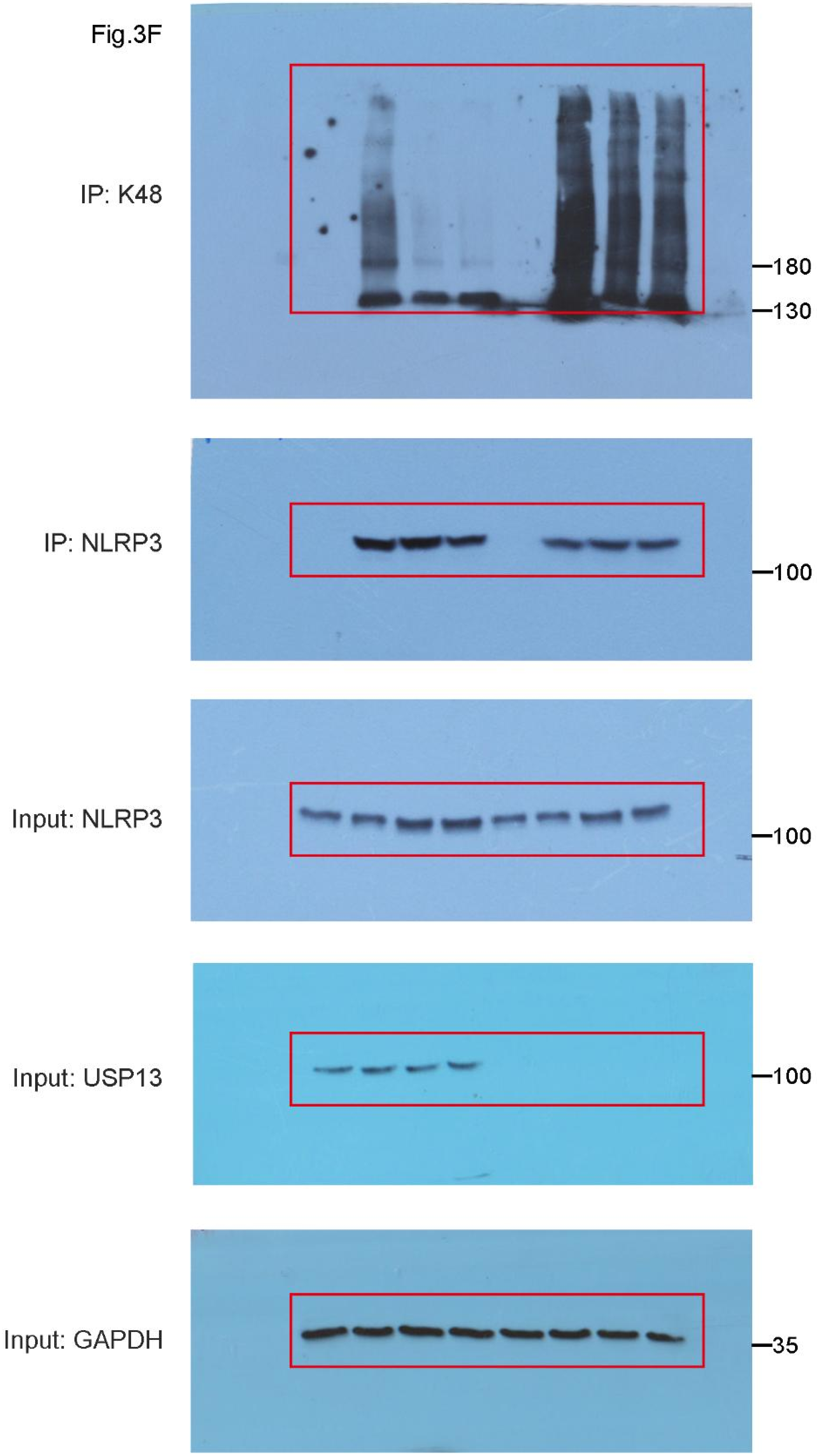

Fig.3F
IP: K48
180
130
IP: NLRP3
100
Input: NLRP3
100
Input: USP13
100
Input: GAPDH
35

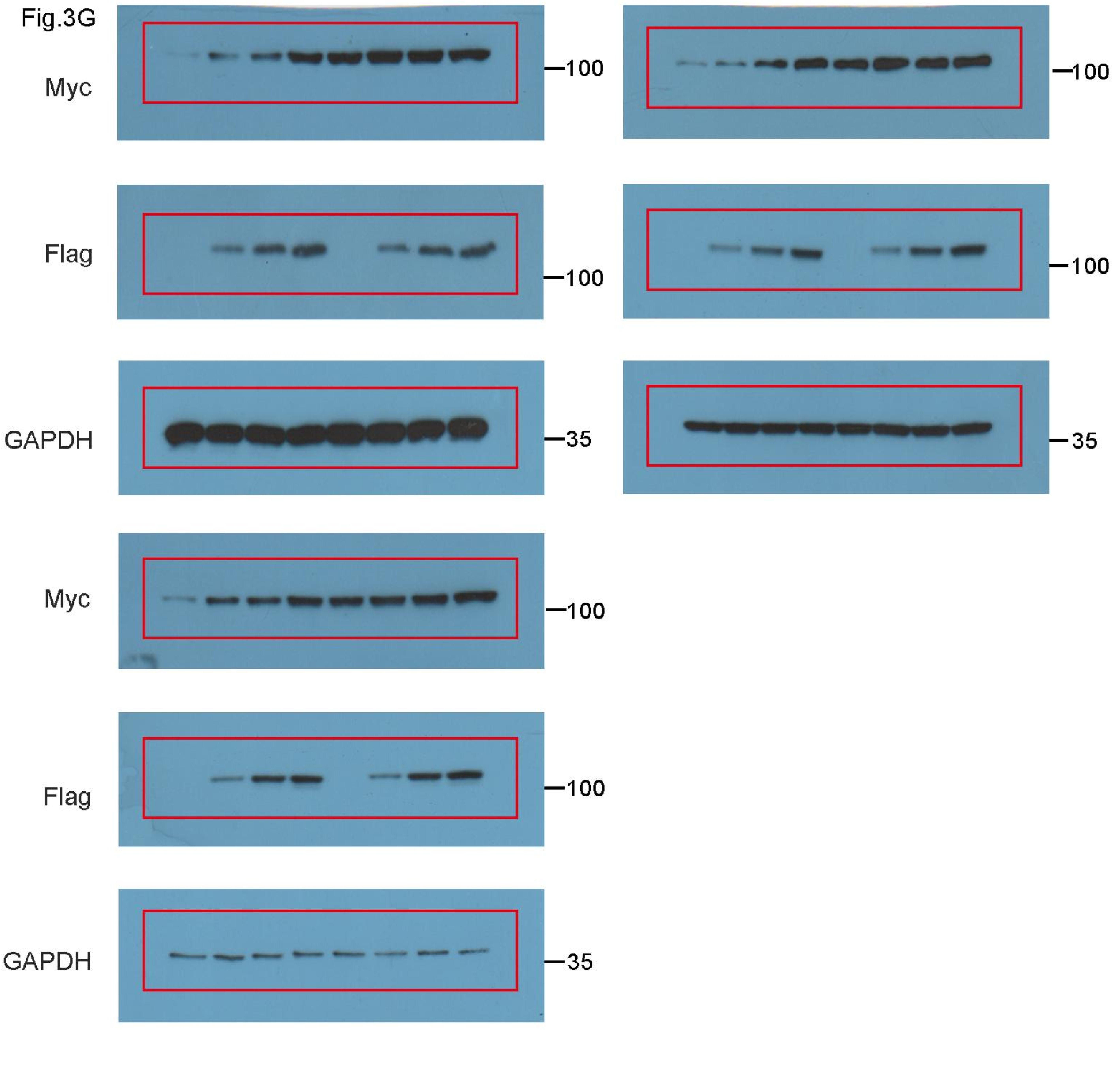
Fig.3G
Myc
Flag
GAPDH
Myc
Flag
GAPDH
100
100
35
100
100
35
100
100
35

Fig.3H

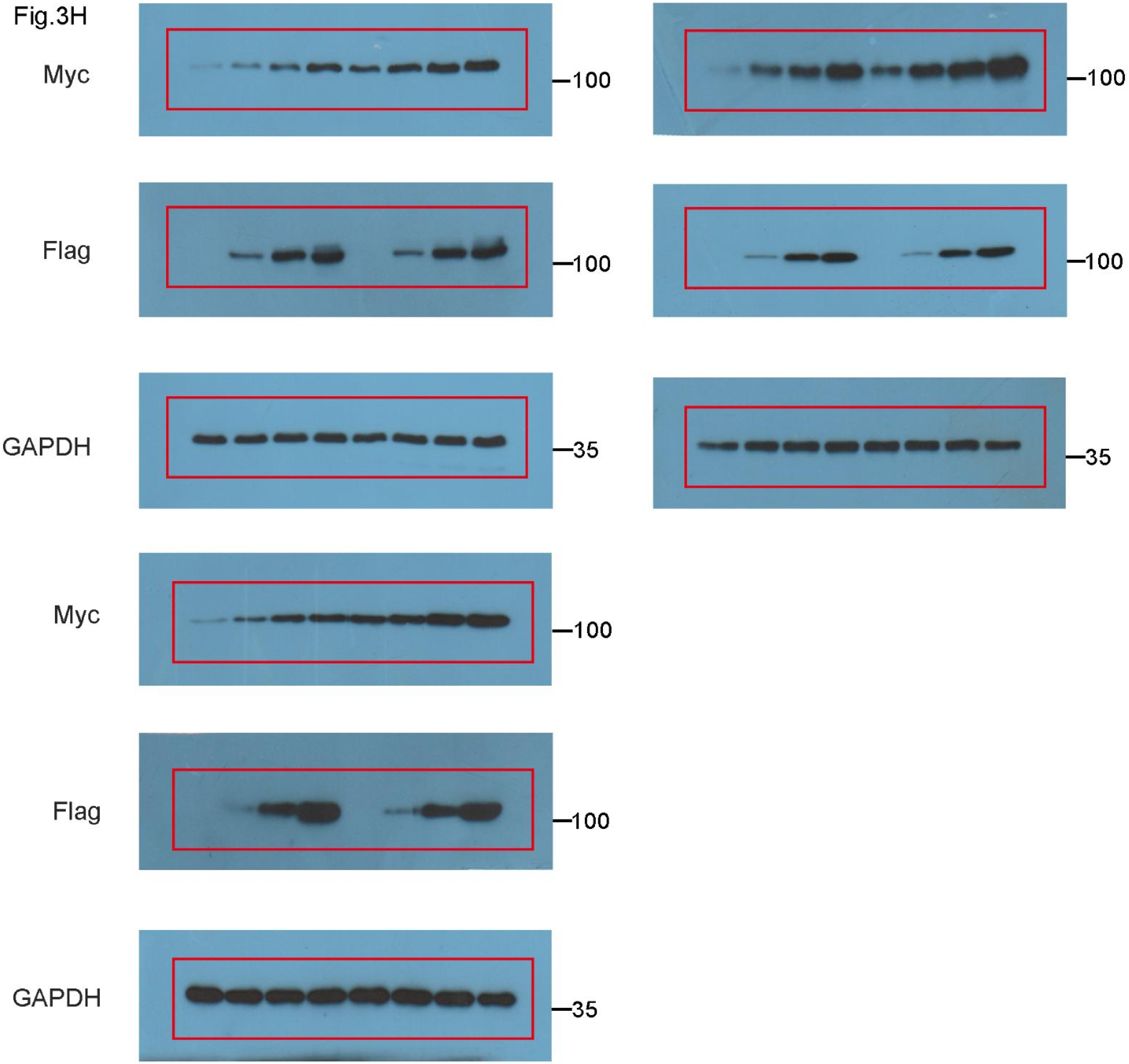

Fig.3I

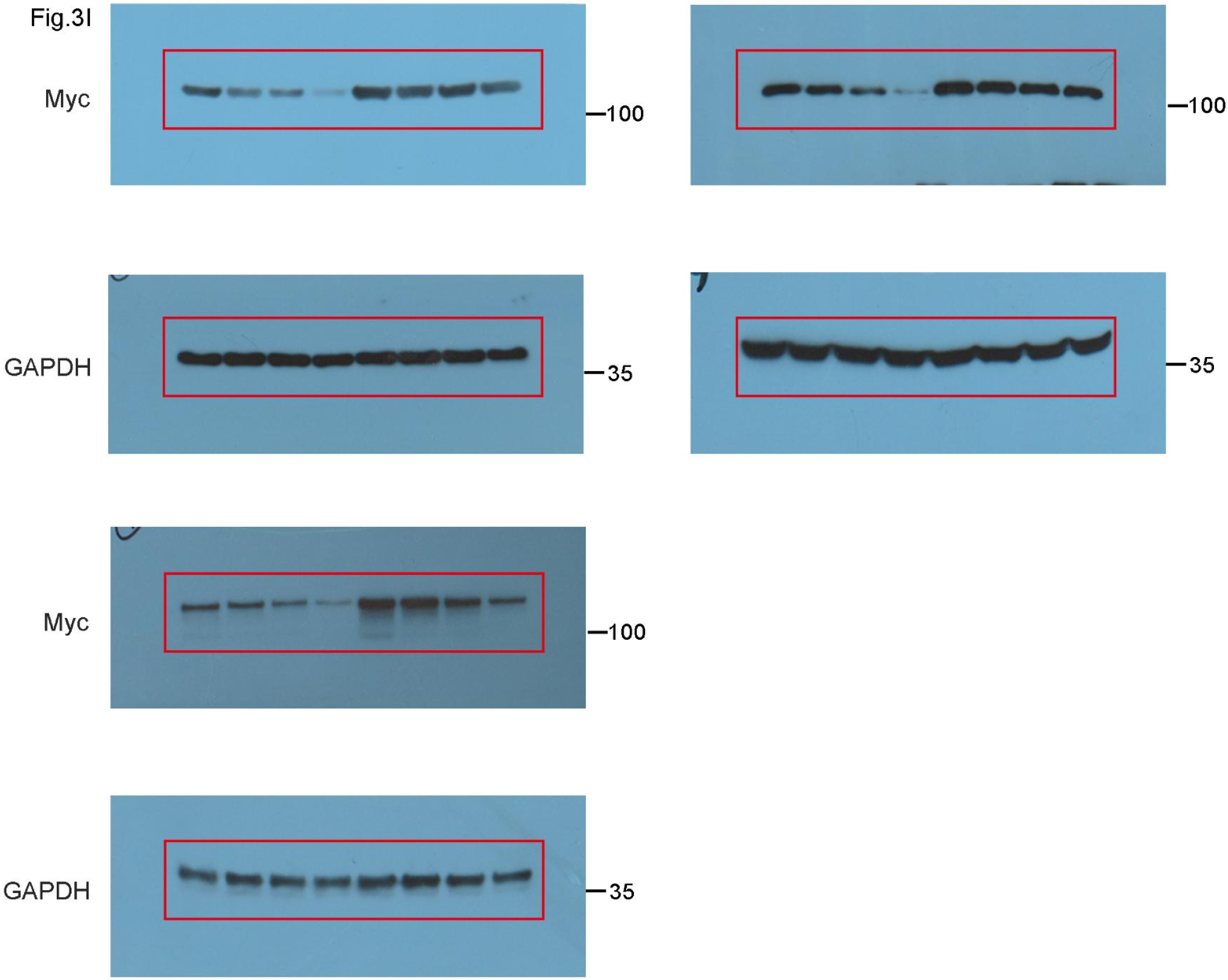

Fig.3J

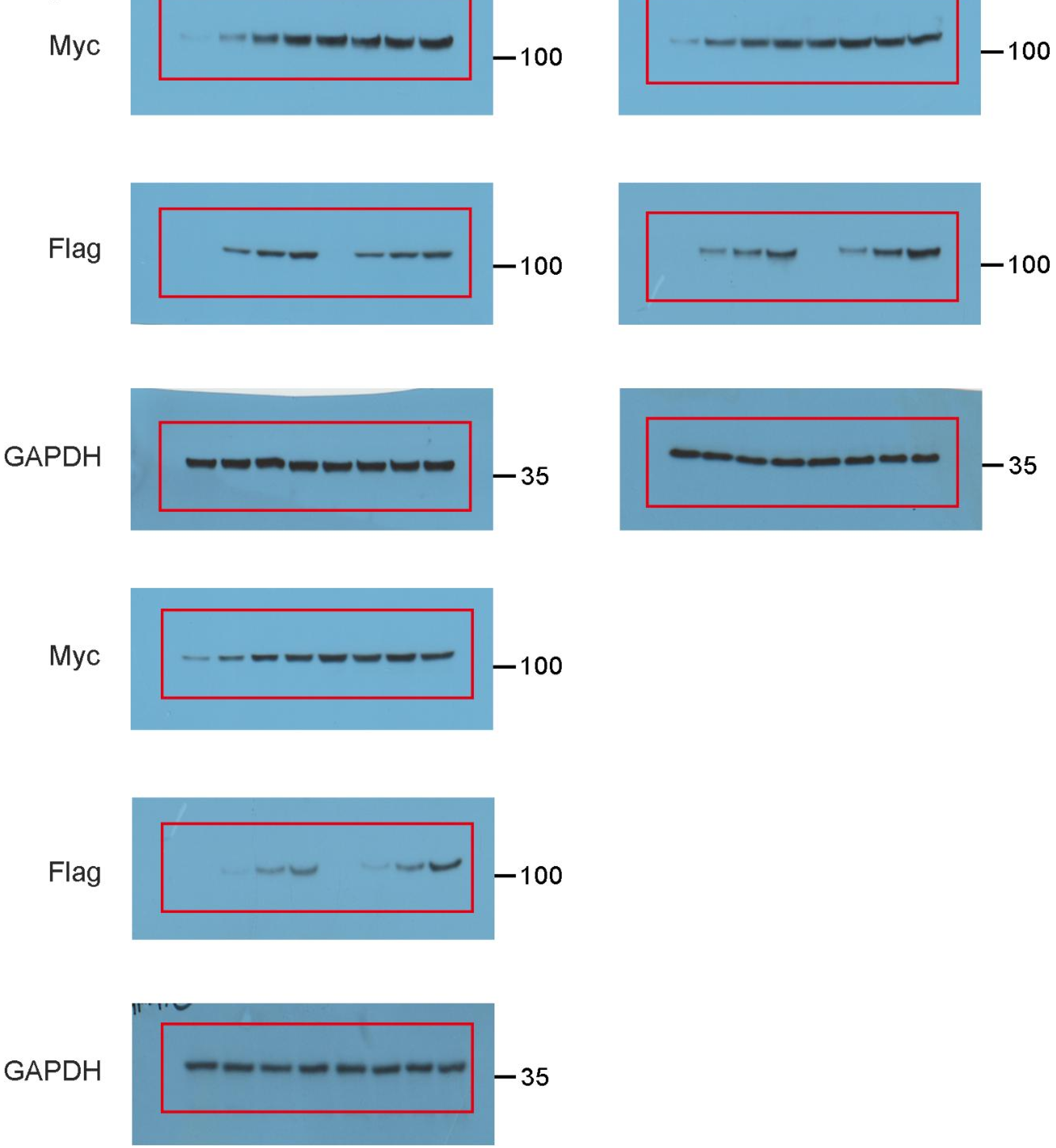

Fig.3K

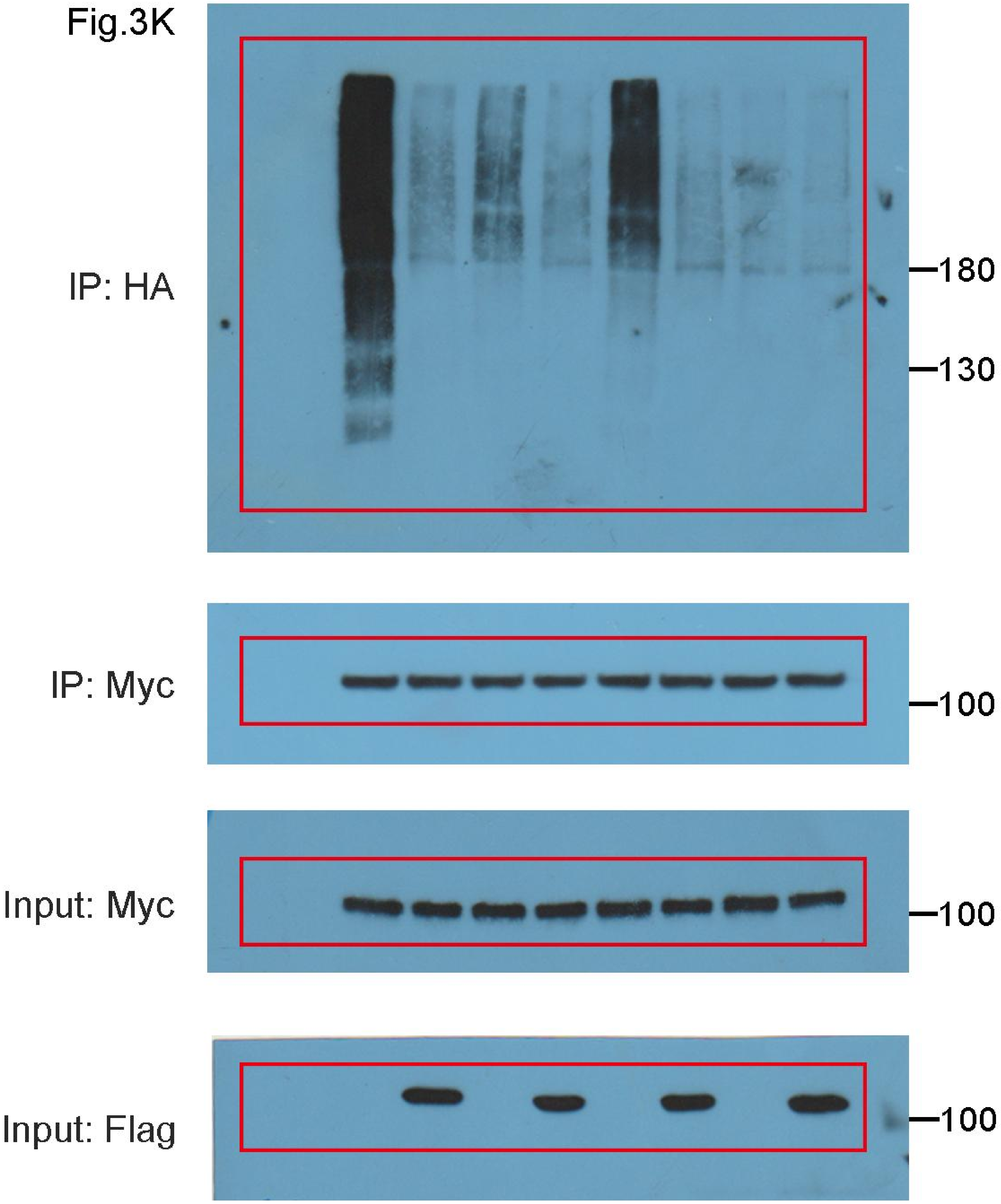

Fig.4A

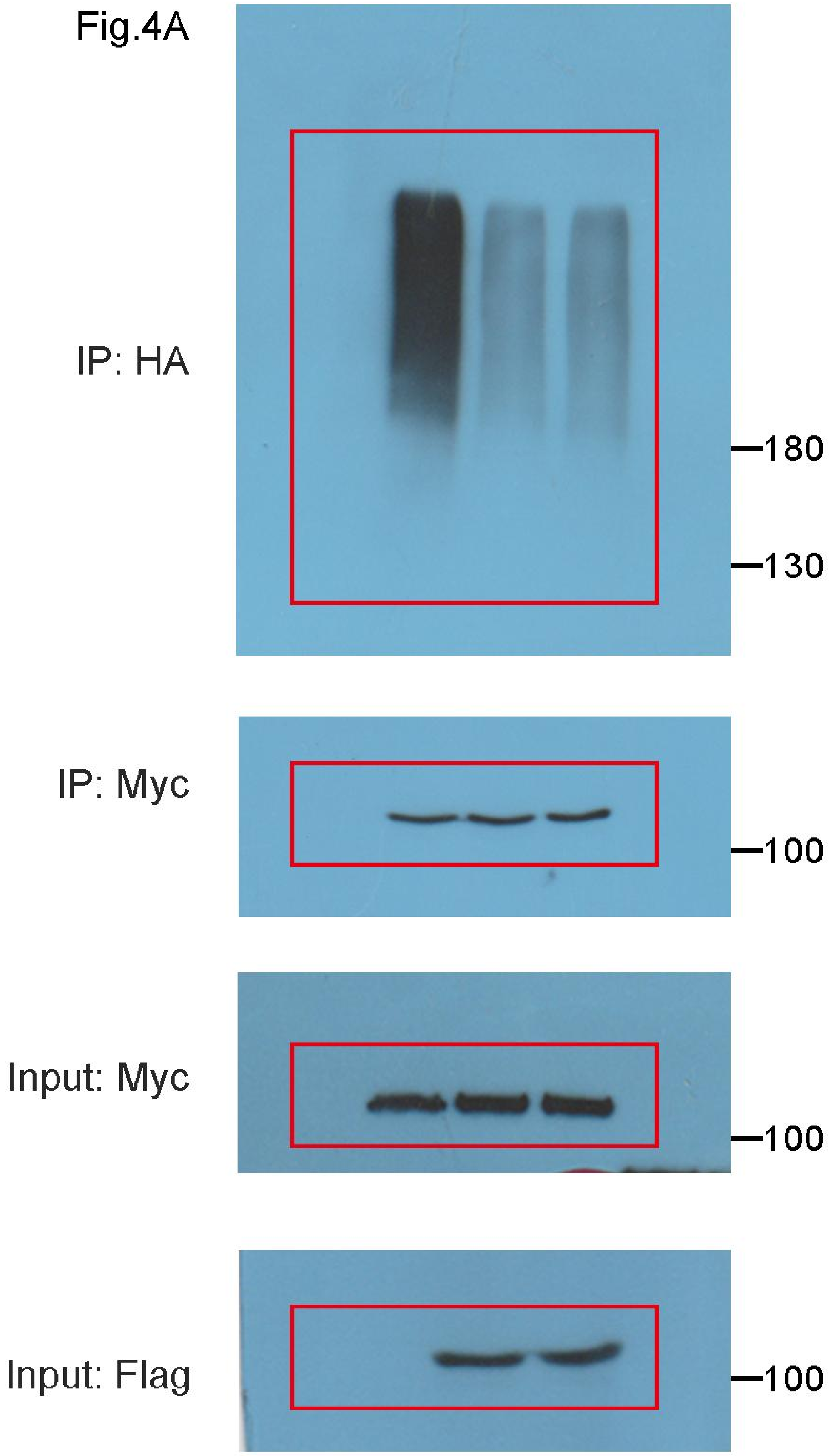

Fig.4B

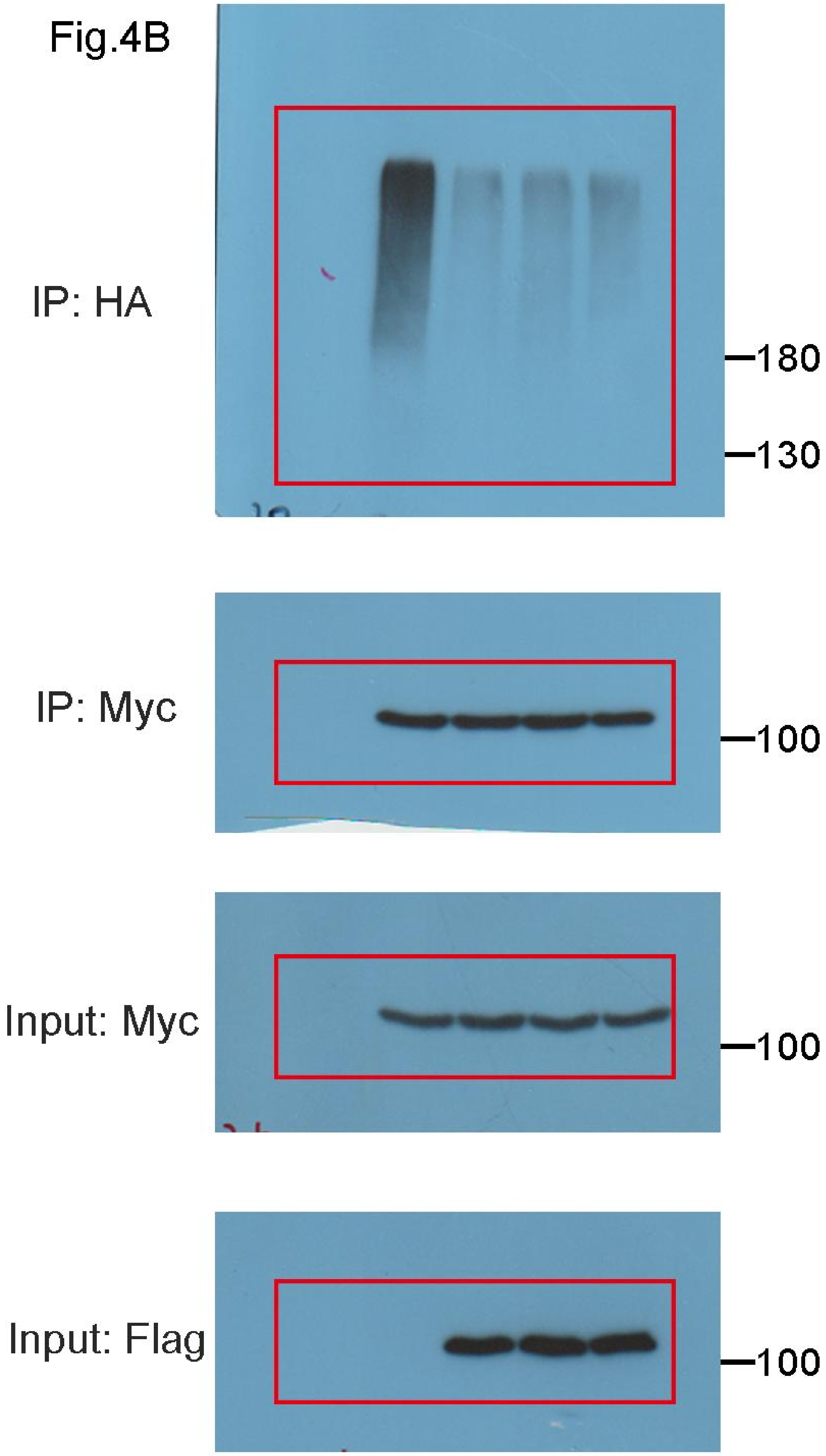

Fig.4C

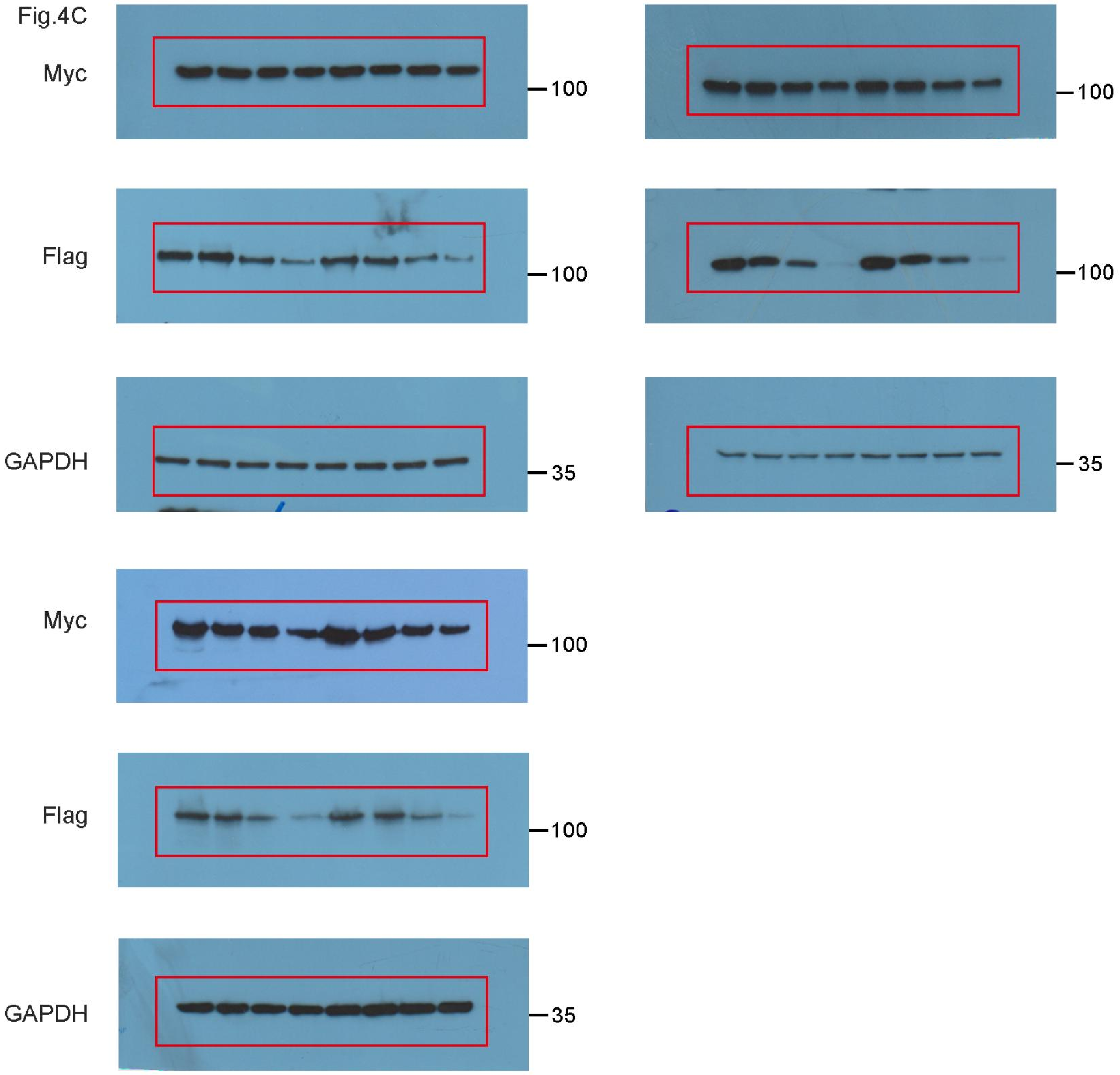

Fig.4D

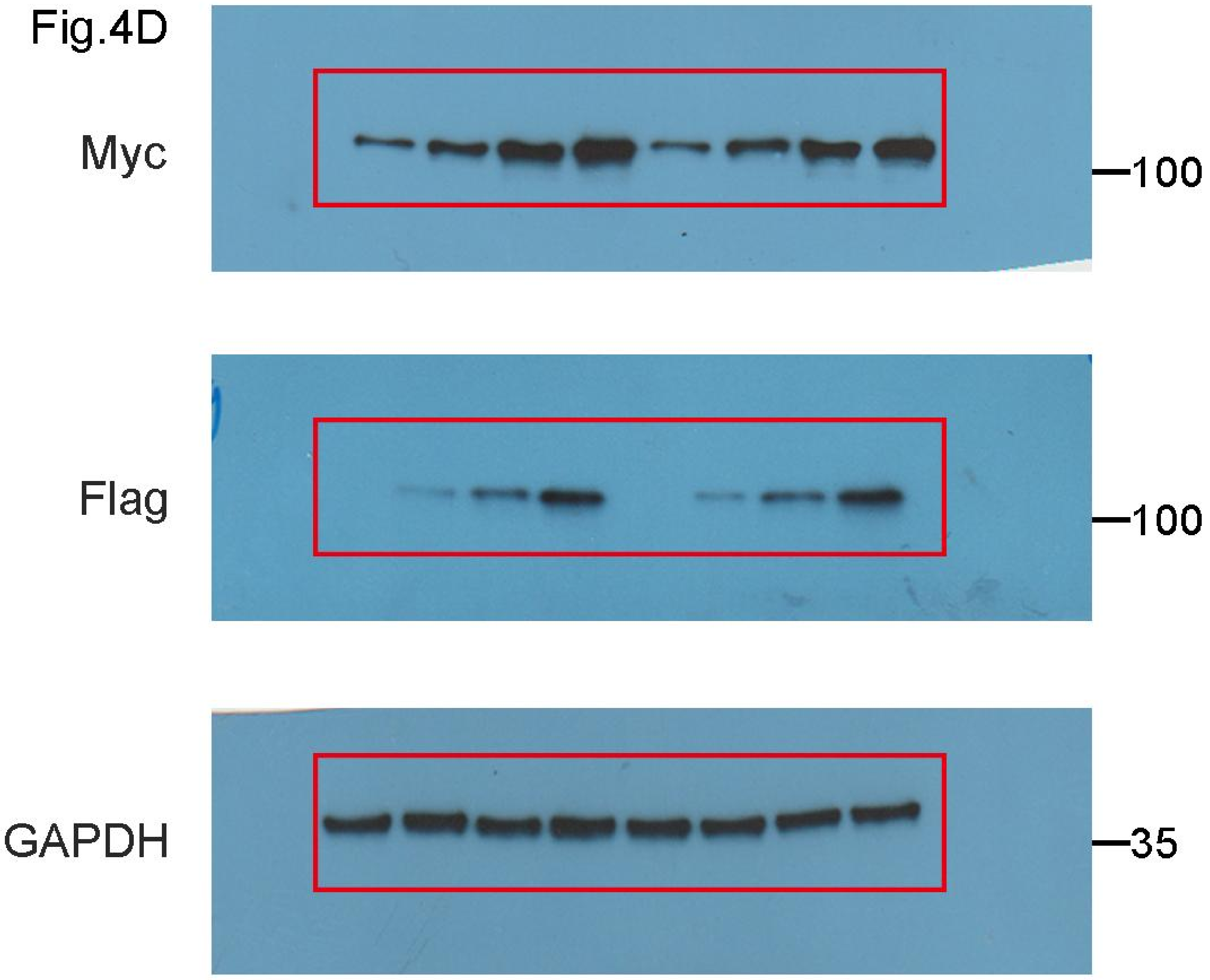

Fig.4E

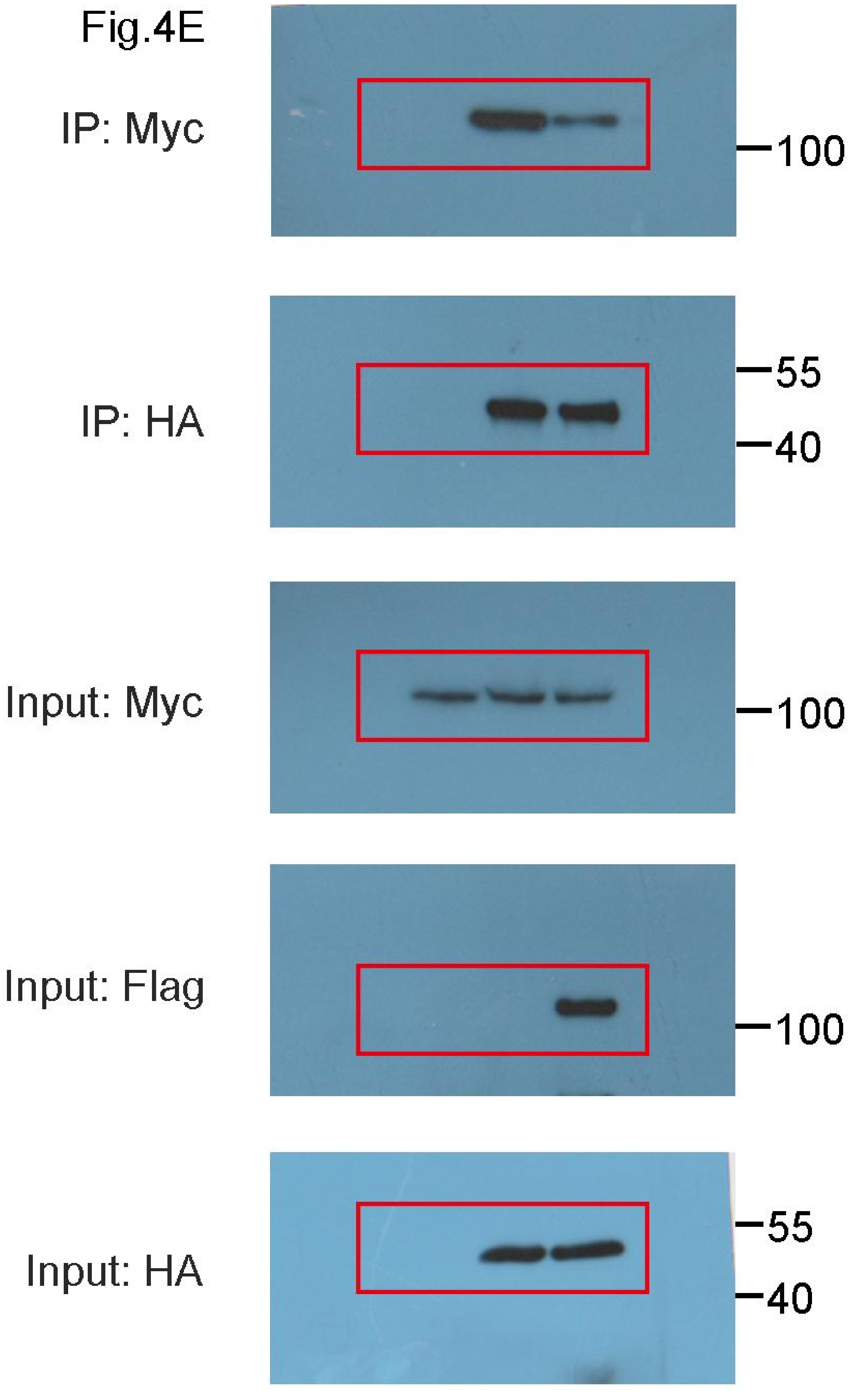

IP: Myc
100
IP: HA
55
40
Input: Myc
100
Input: Flag
100
Input: HA
55
40

Fig.4F

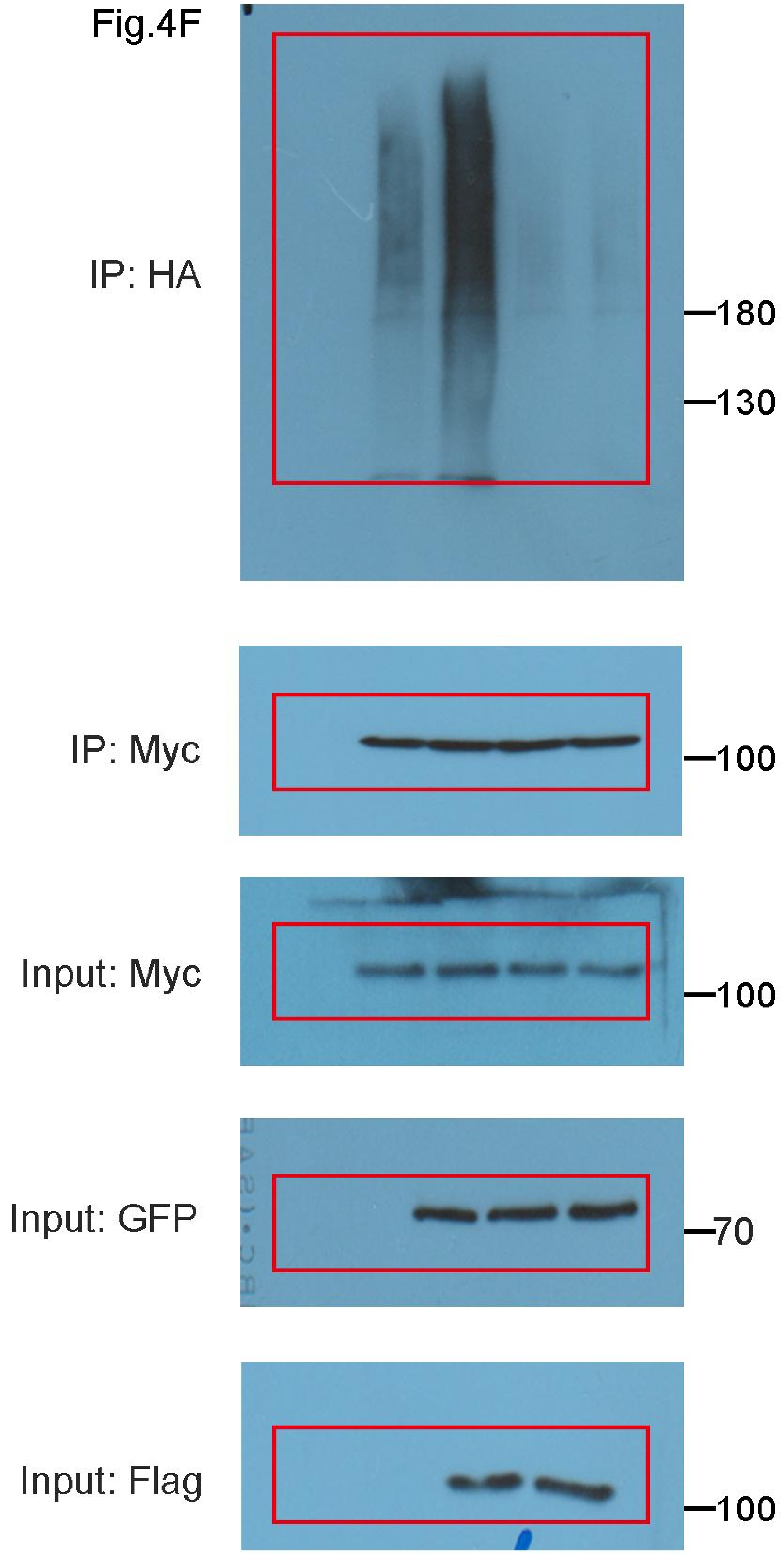
IP: HA
180
130
IP: Myc
100
Input: Myc
100
Input: GFP
70
Input: Flag
100

Fig.4G

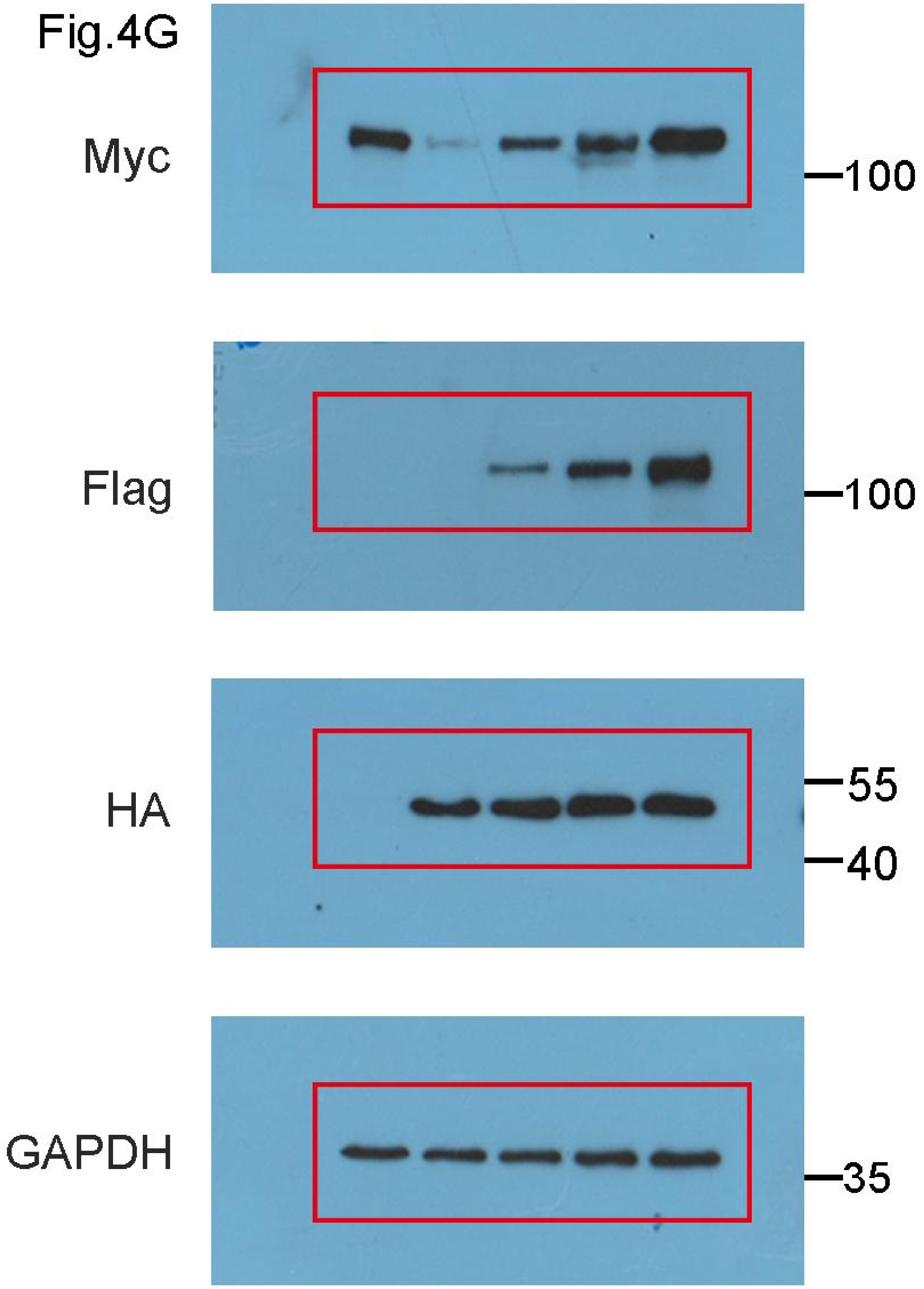

Fig.4H

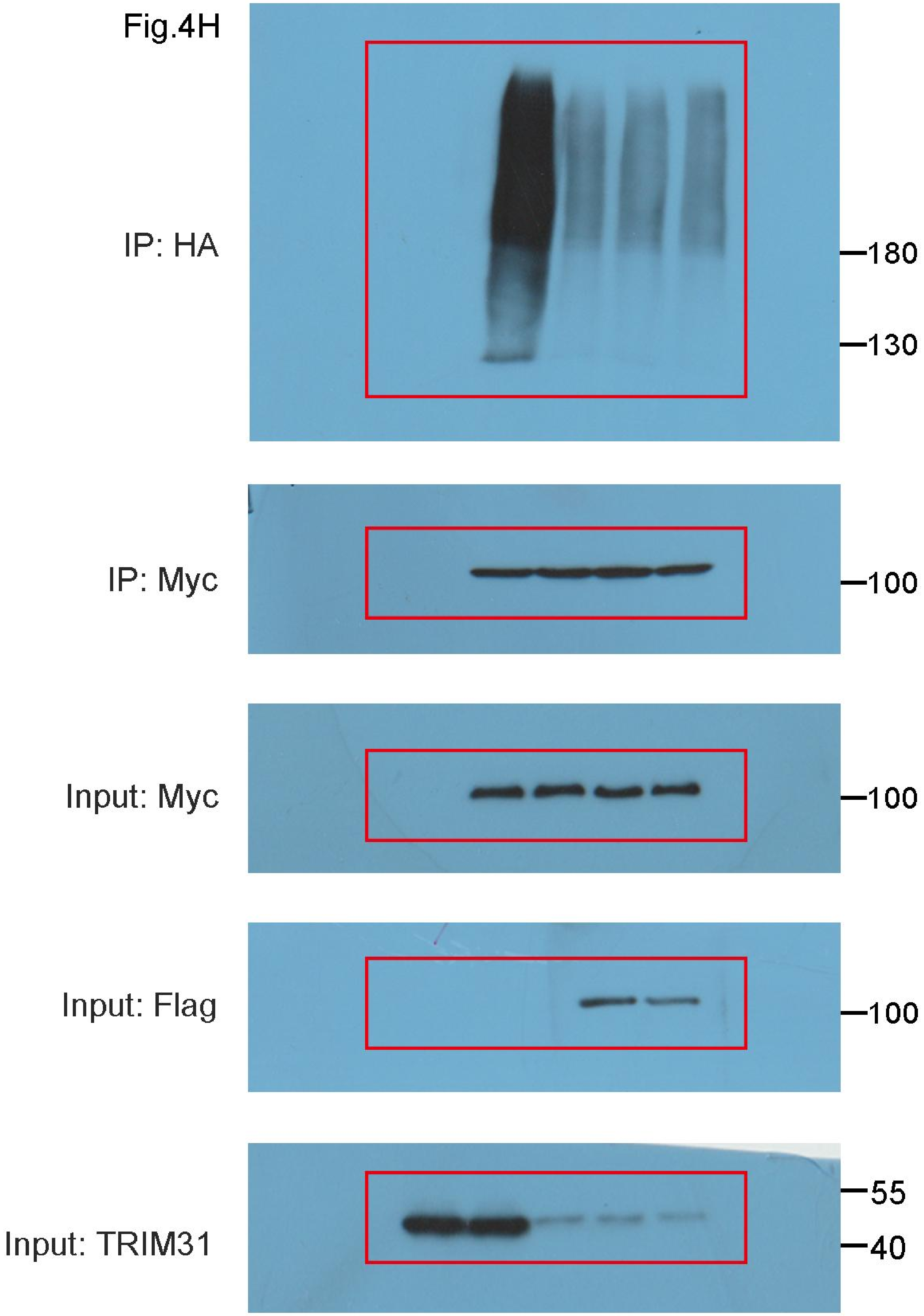
IP: HA
180
130
IP: Myc
100
Input: Myc
100
Input: Flag
100
Input: TRIM31
55
40

Fig.4I

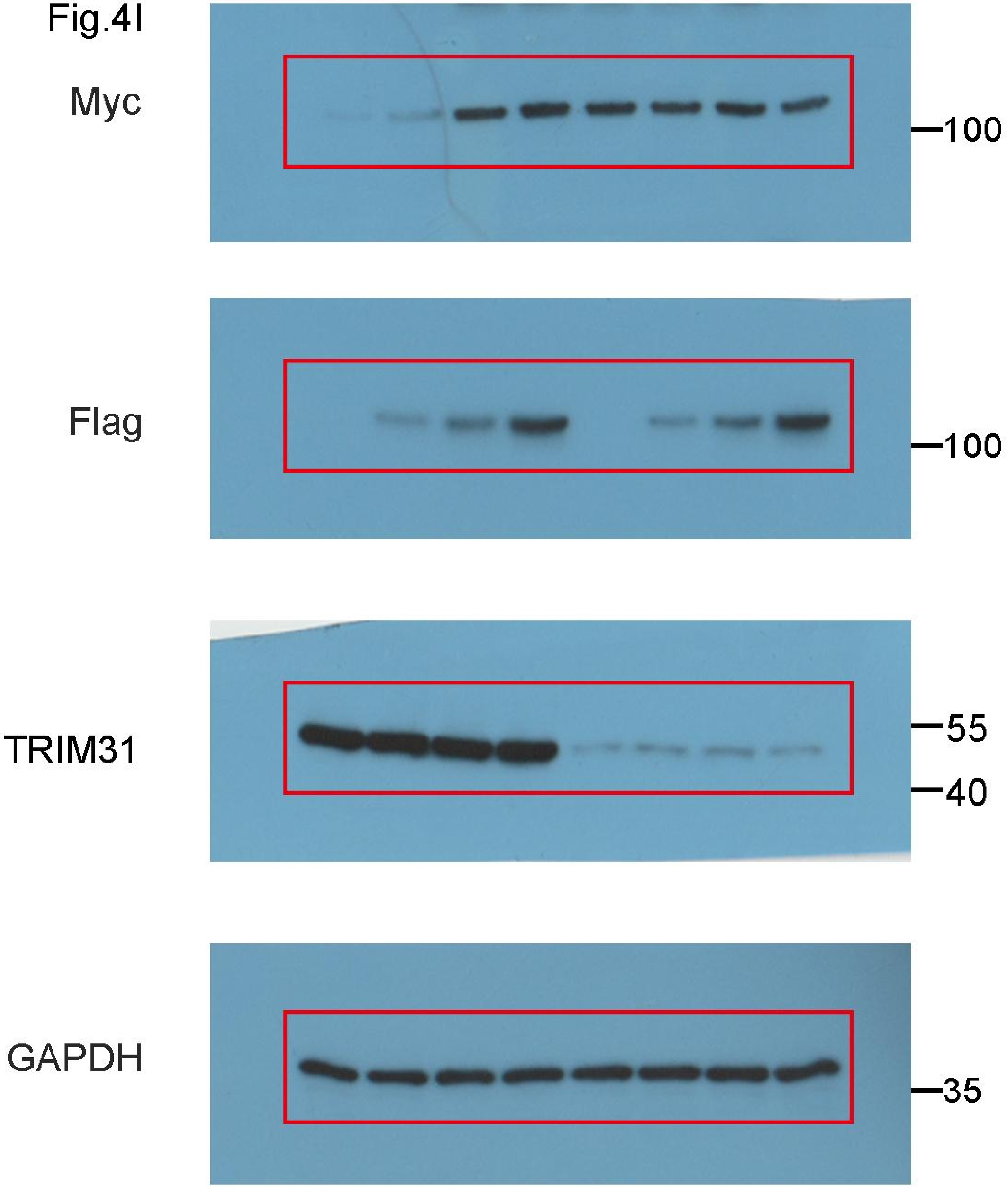

Fig.4J

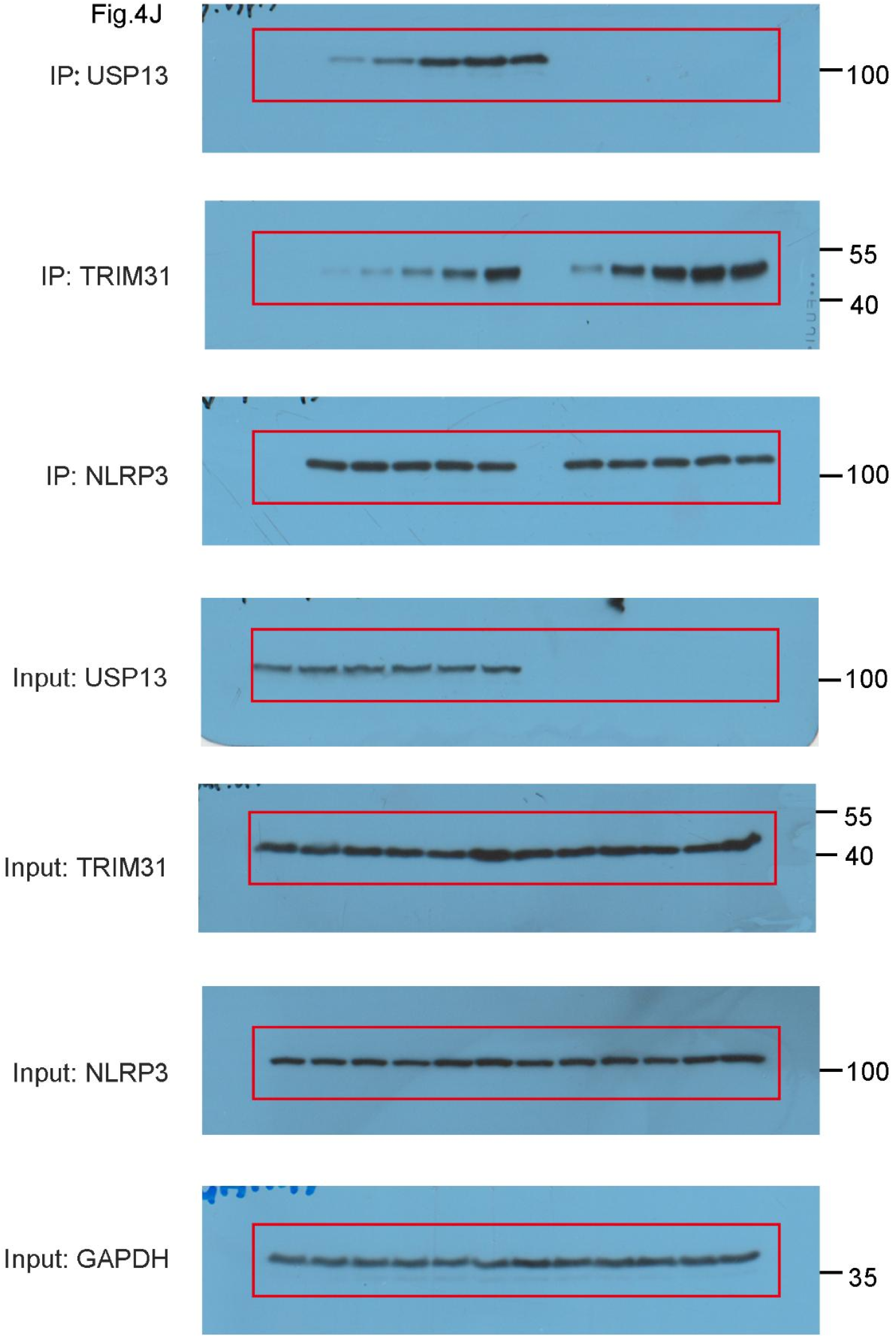
IP: USP13
100
IP: TRIM31
55
40
IP: NLRP3
100
Input: USP13
100
Input: TRIM31
55
40
Input: NLRP3
100
Input: GAPDH
35

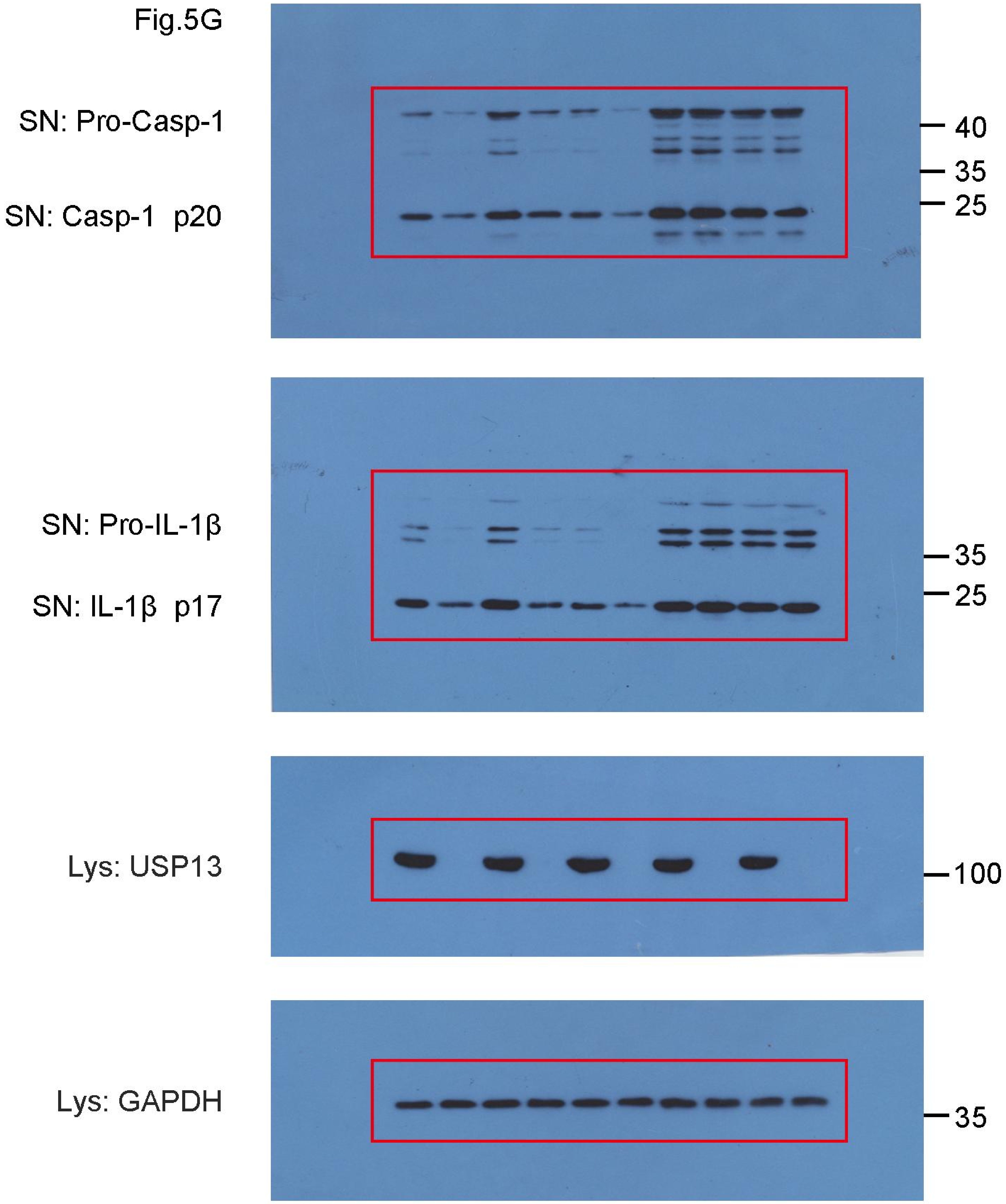

Fig.5G
SN: Pro-Casp-1
SN: Casp-1 p20
40
35
25
SN: Pro-IL-1β
SN: IL-1β p17
35
25
Lys: USP13
100
Lys: GAPDH
35

Fig.6A

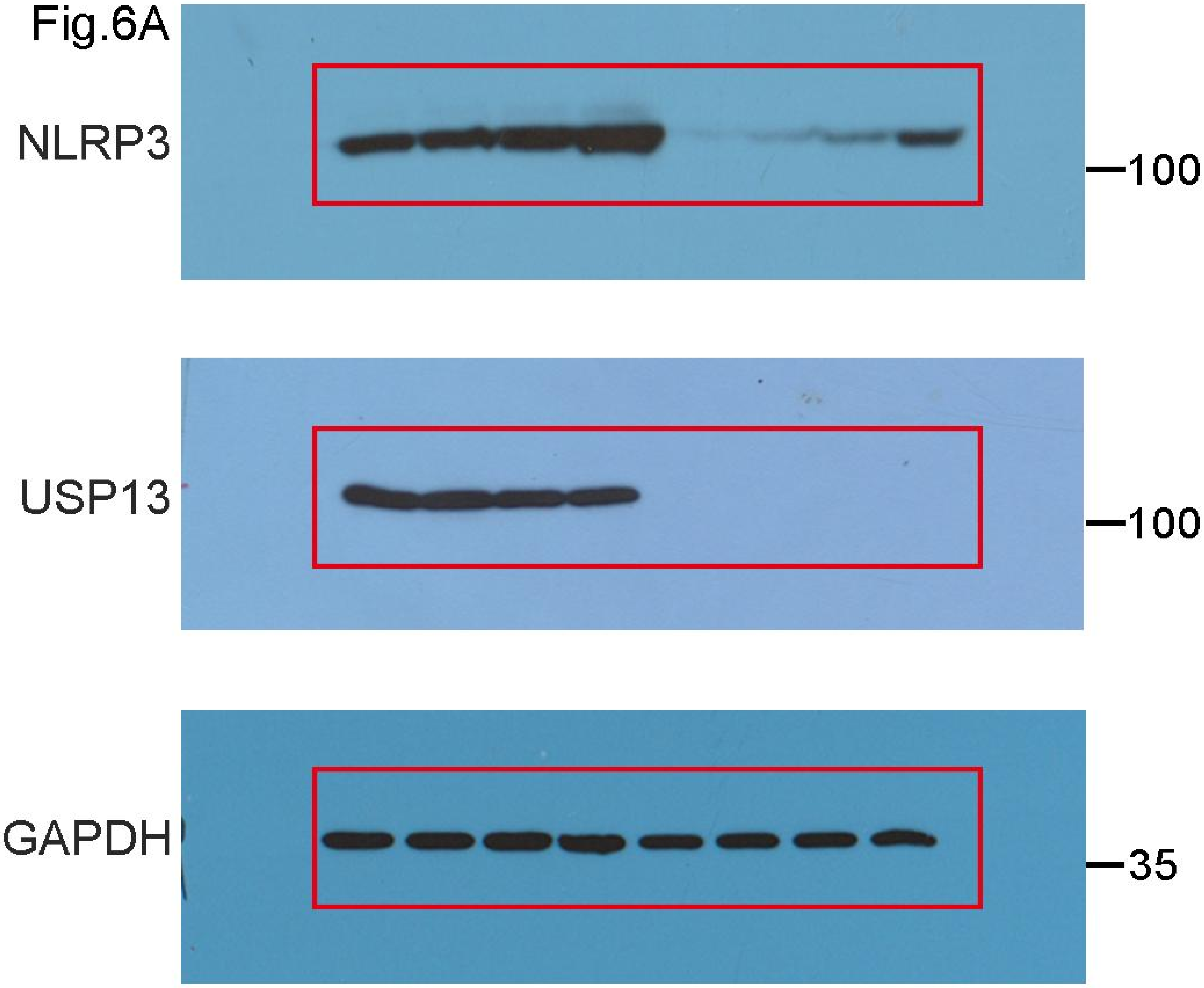

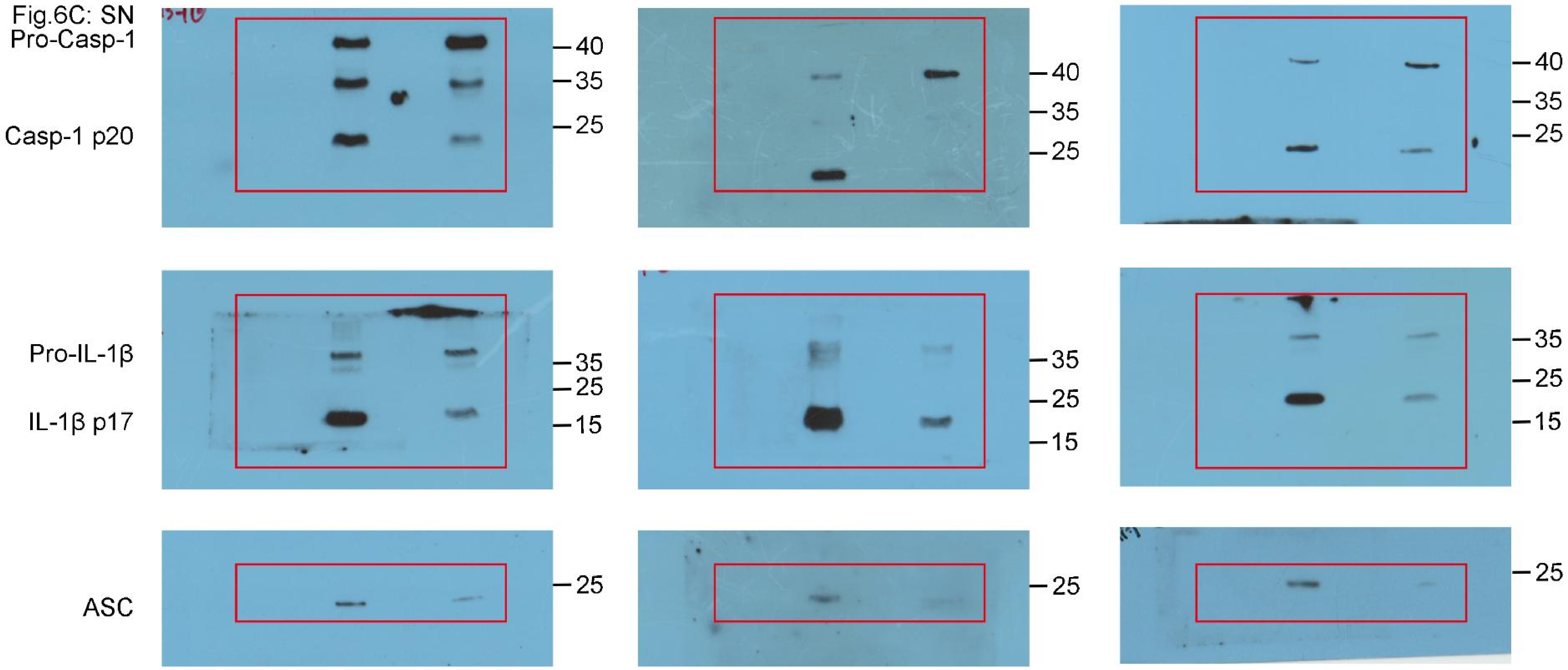

Fig.6C: SN
Pro-Casp-1
Casp-1 p20
Pro-IL-1β
IL-1β p17
ASC
40
35
25
15

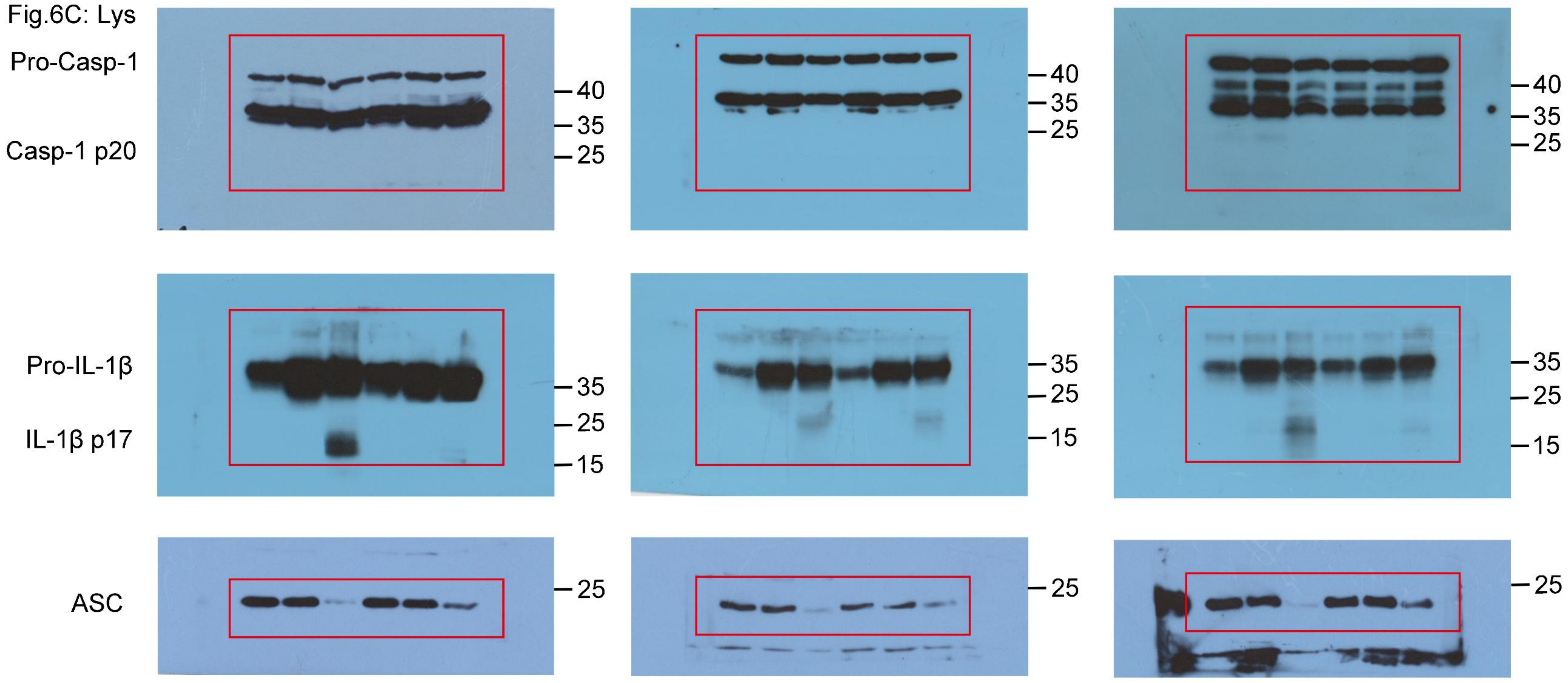

Fig.6C: Lys
Pro-Casp-1
Casp-1 p20
40
35
25
40
35
25
40
35
25
Pro-IL-1β
IL-1β p17
35
25
15
35
25
15
35
25
15
ASC
25
25
25

Fig.6C: Lys

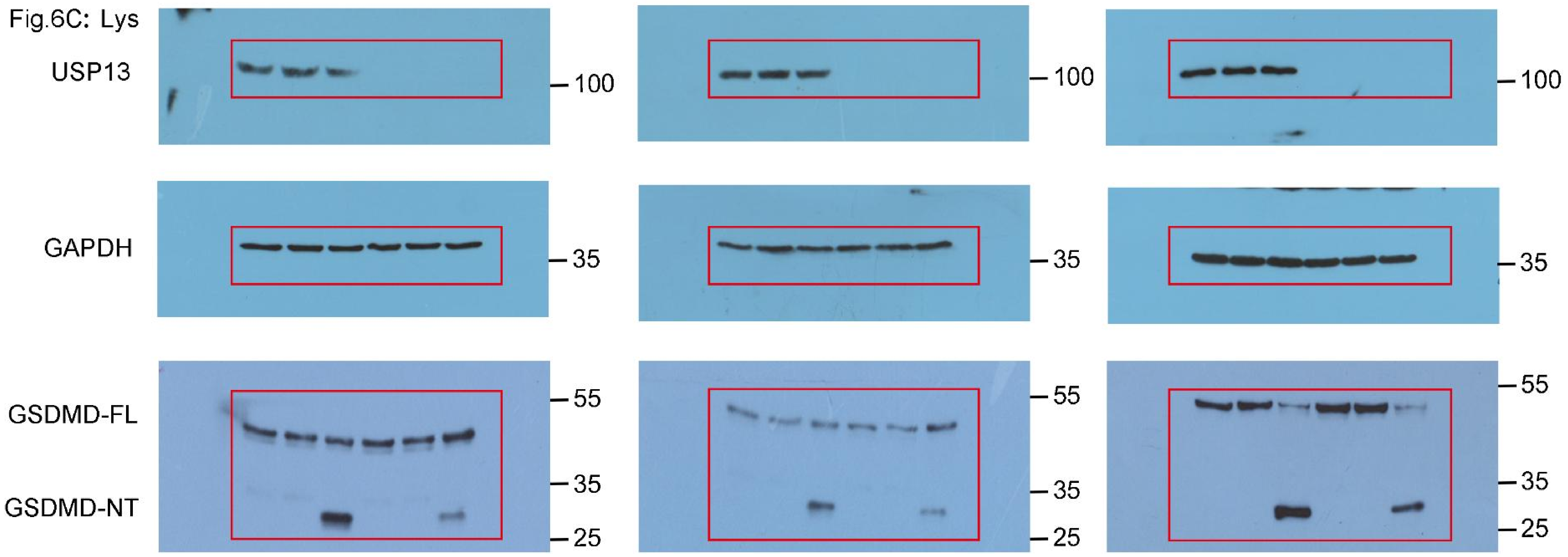

USP13
100
100
100
GAPDH
35
35
35
GSDMD-FL
GSDMD-NT
55
35
25
55
35
25
55
35
25

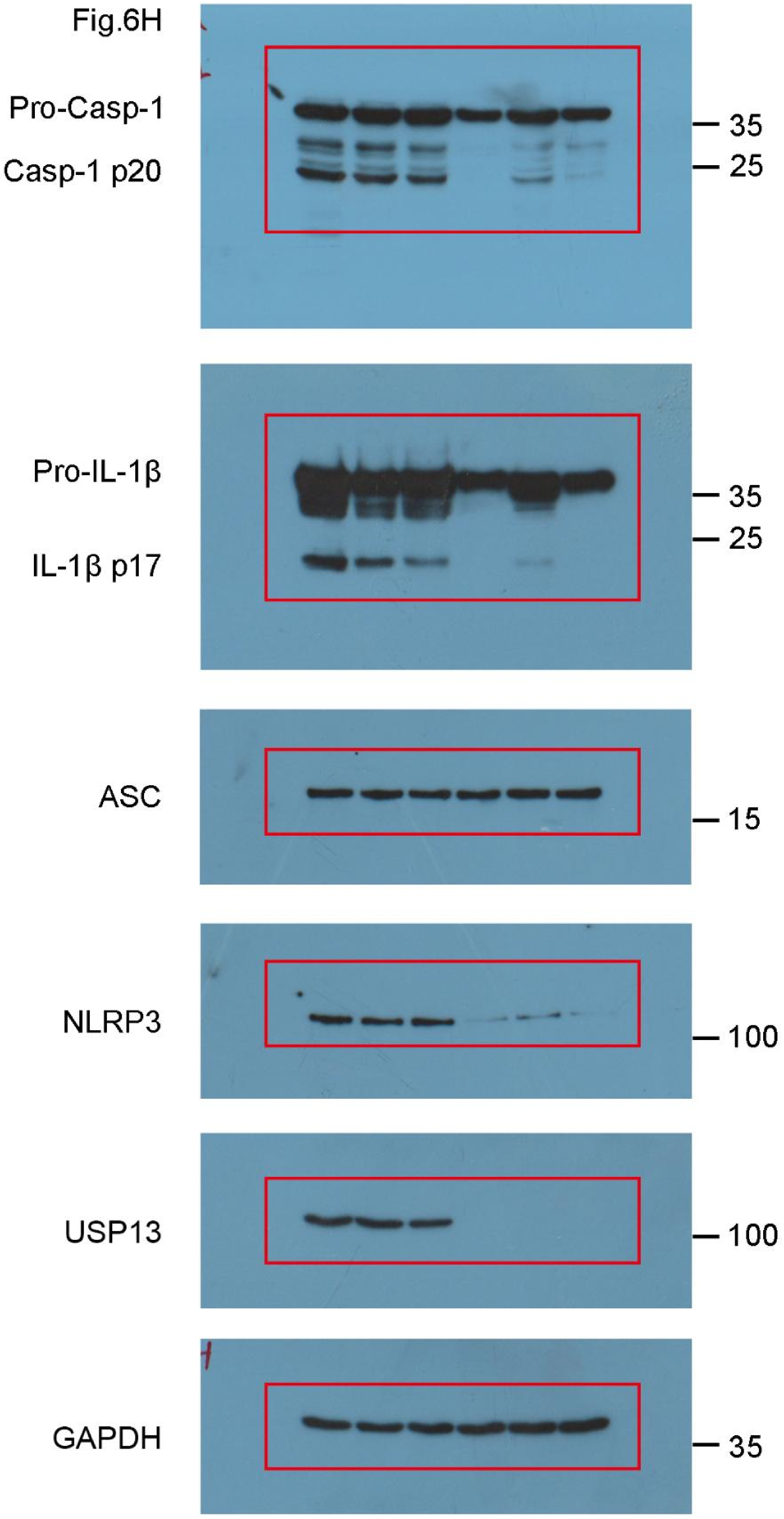
Fig.6H
Pro-Casp-1
Casp-1 p20
35
25
Pro-IL-1β
IL-1β p17
35
25
ASC
15
NLRP3
100
USP13
100
GAPDH
35

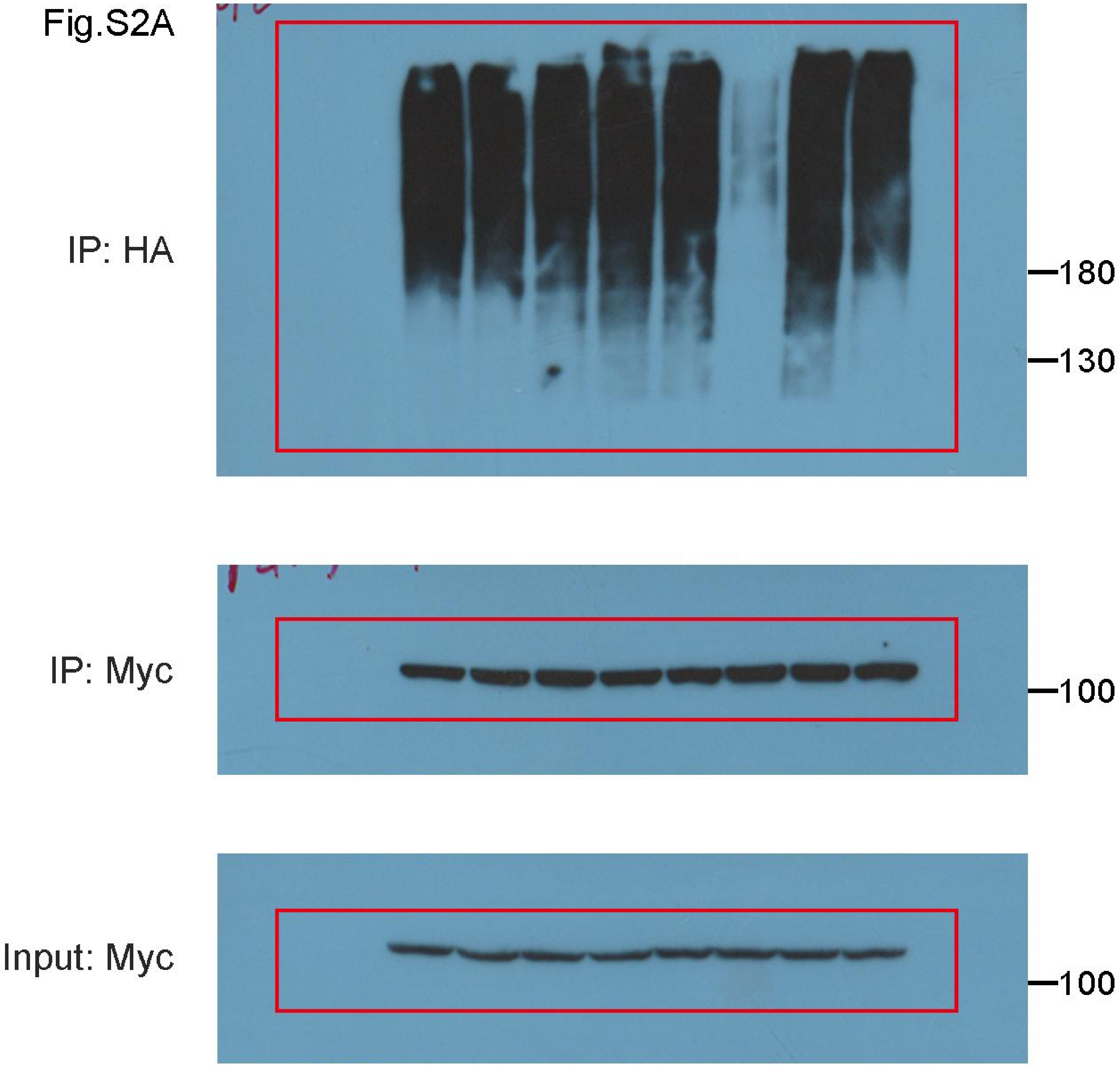
Fig.S2A
IP: HA
180
130
IP: Myc
100
Input: Myc
100

Fig.S2B

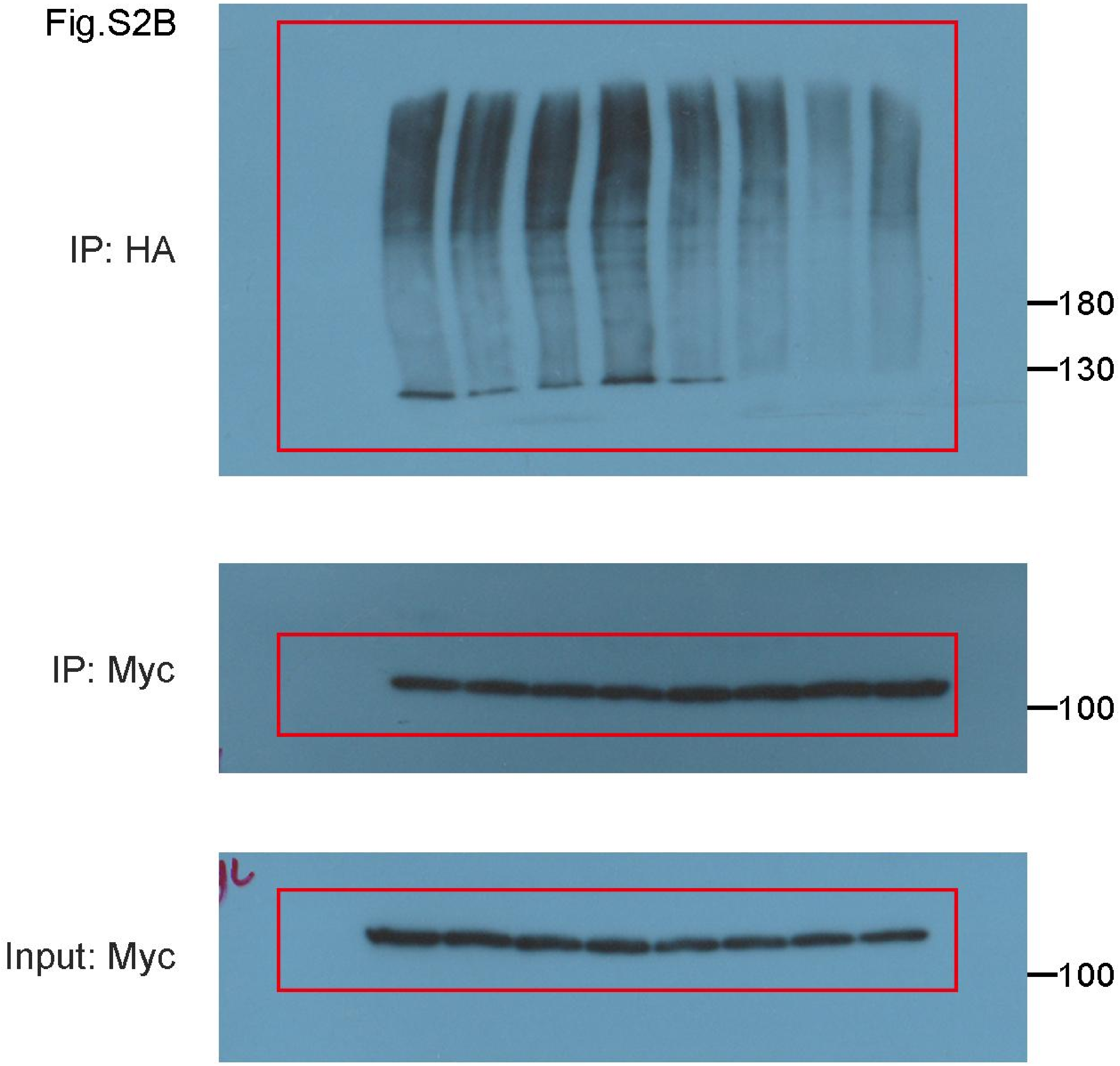

Fig.S2C

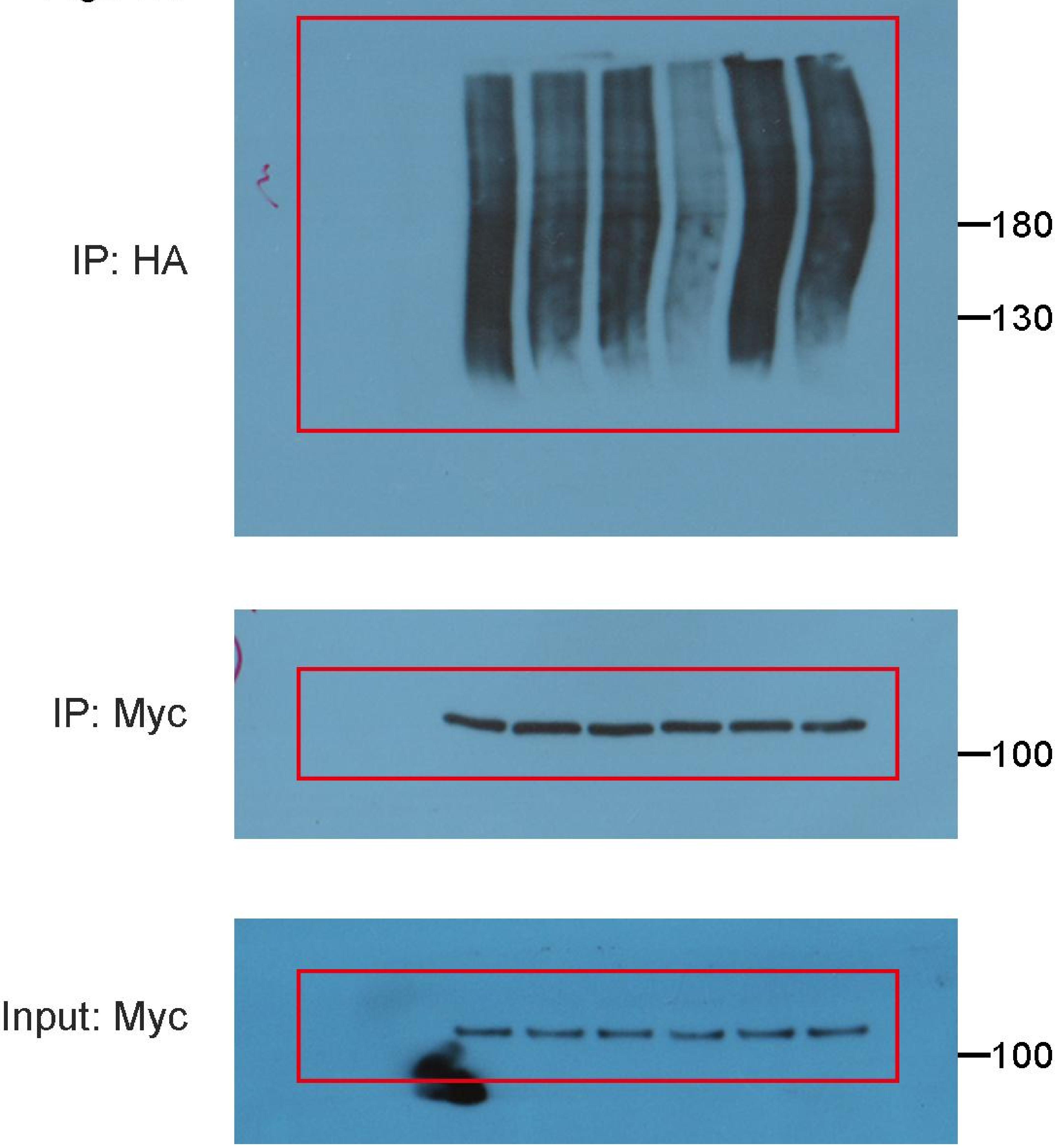

Fig.S3A

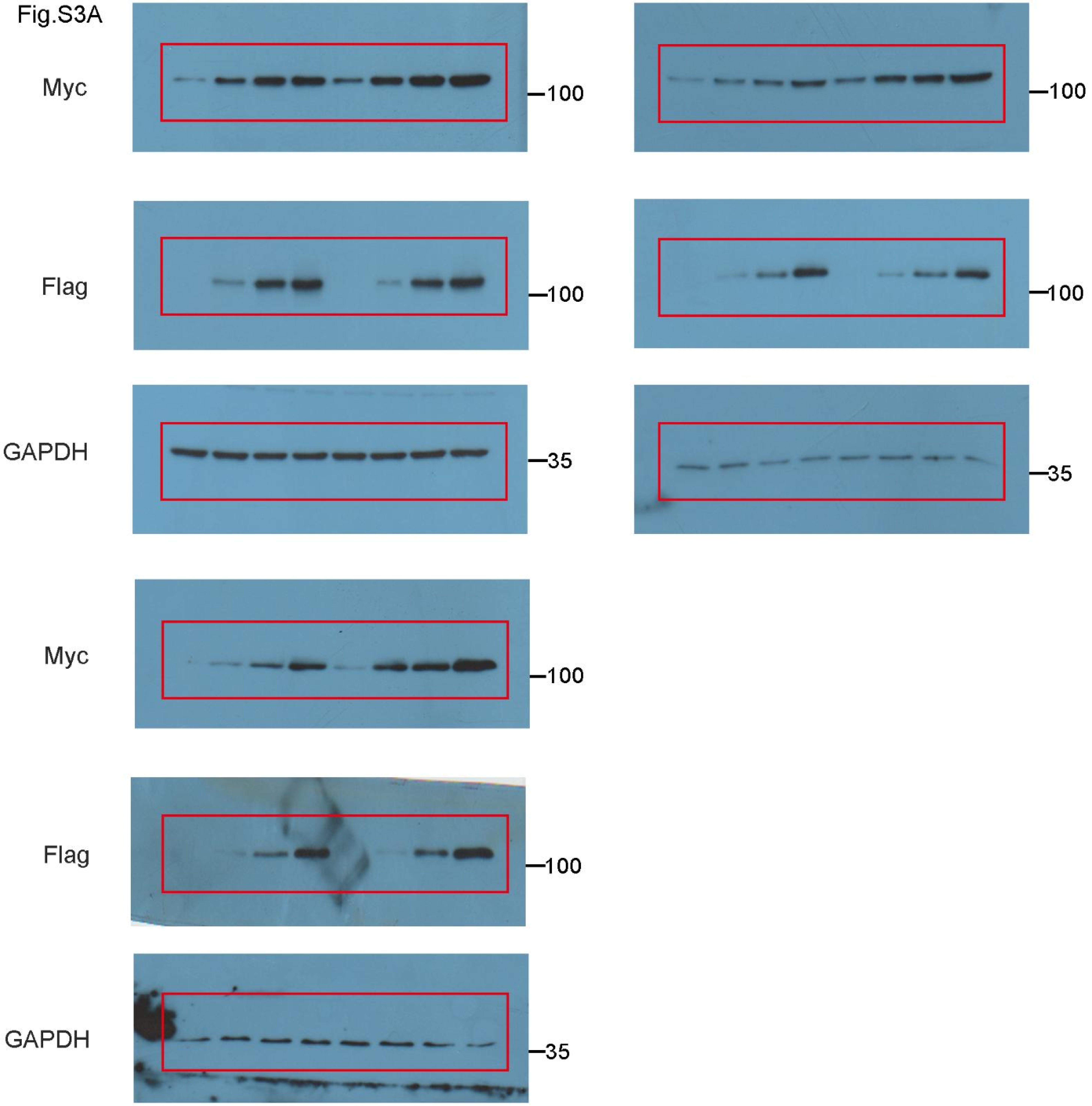

Fig.S4A

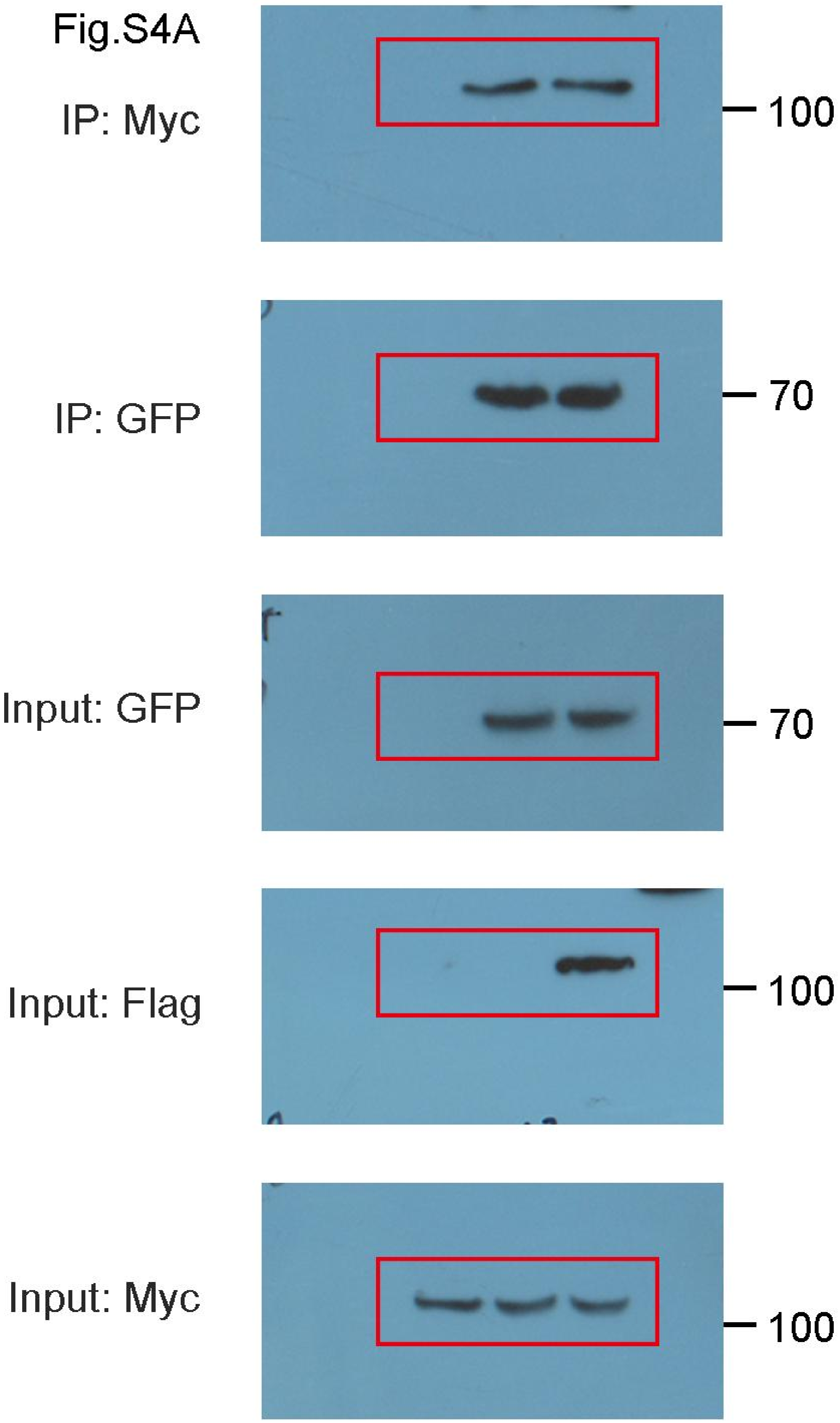

Fig.S4B

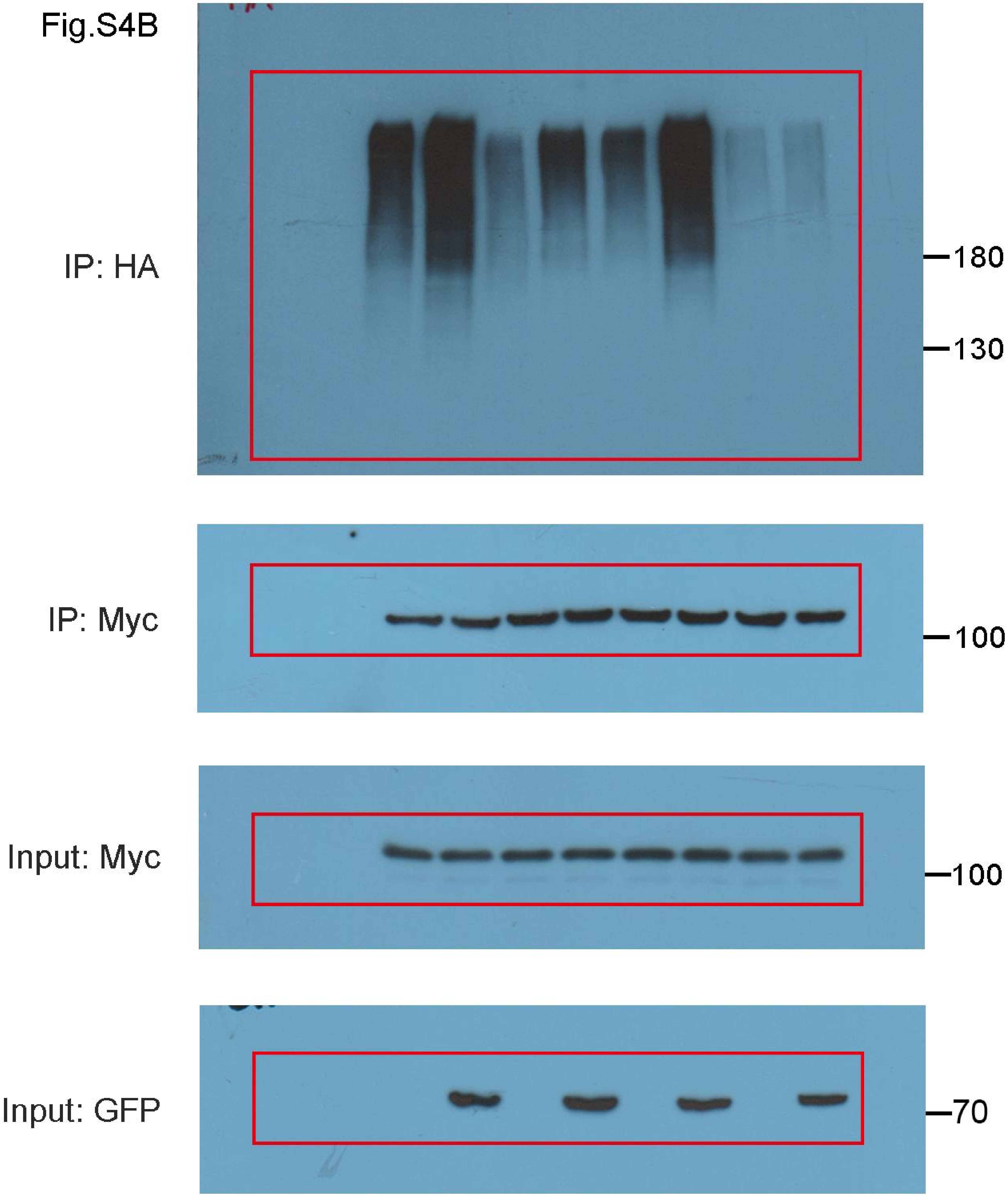

Fig.S4C

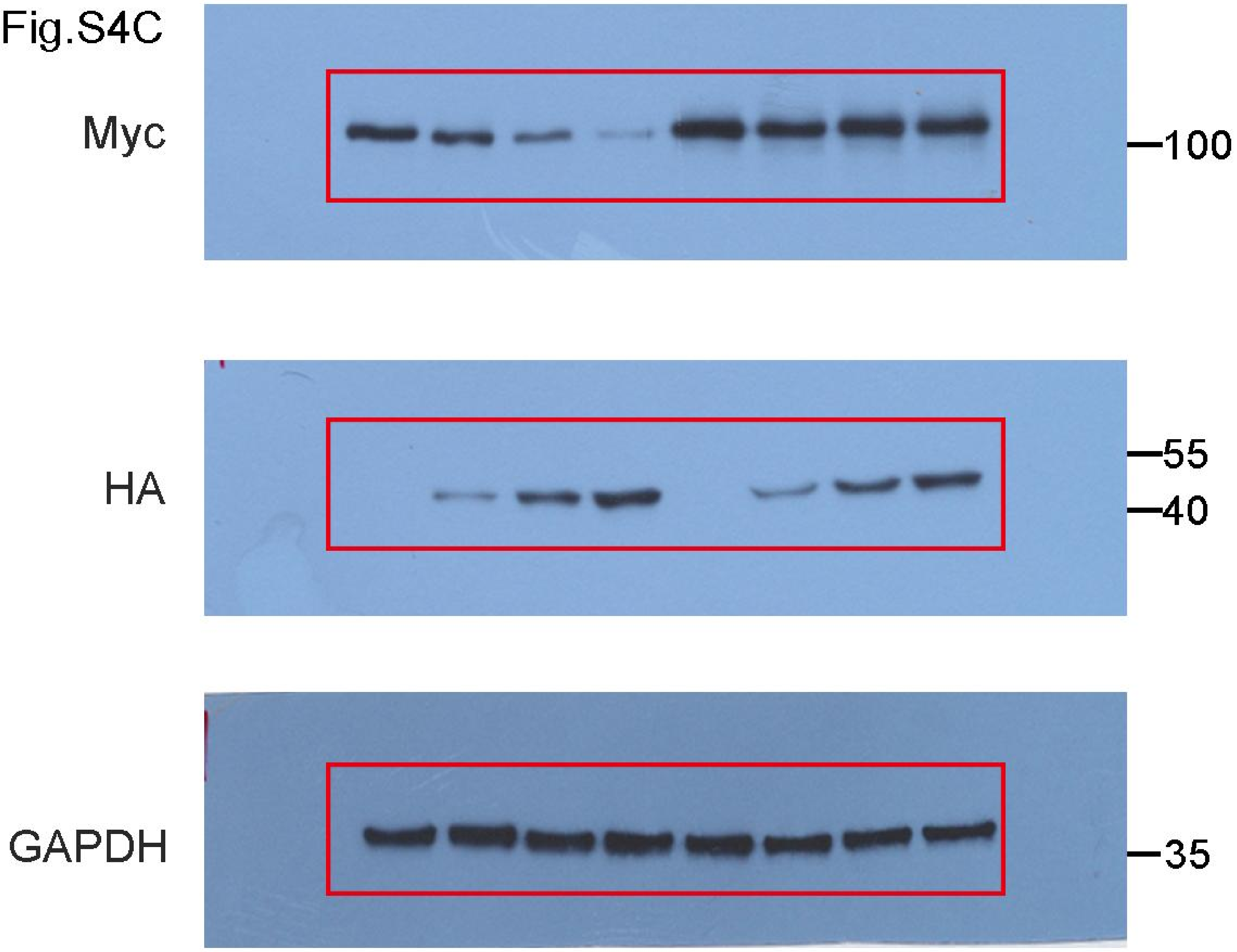

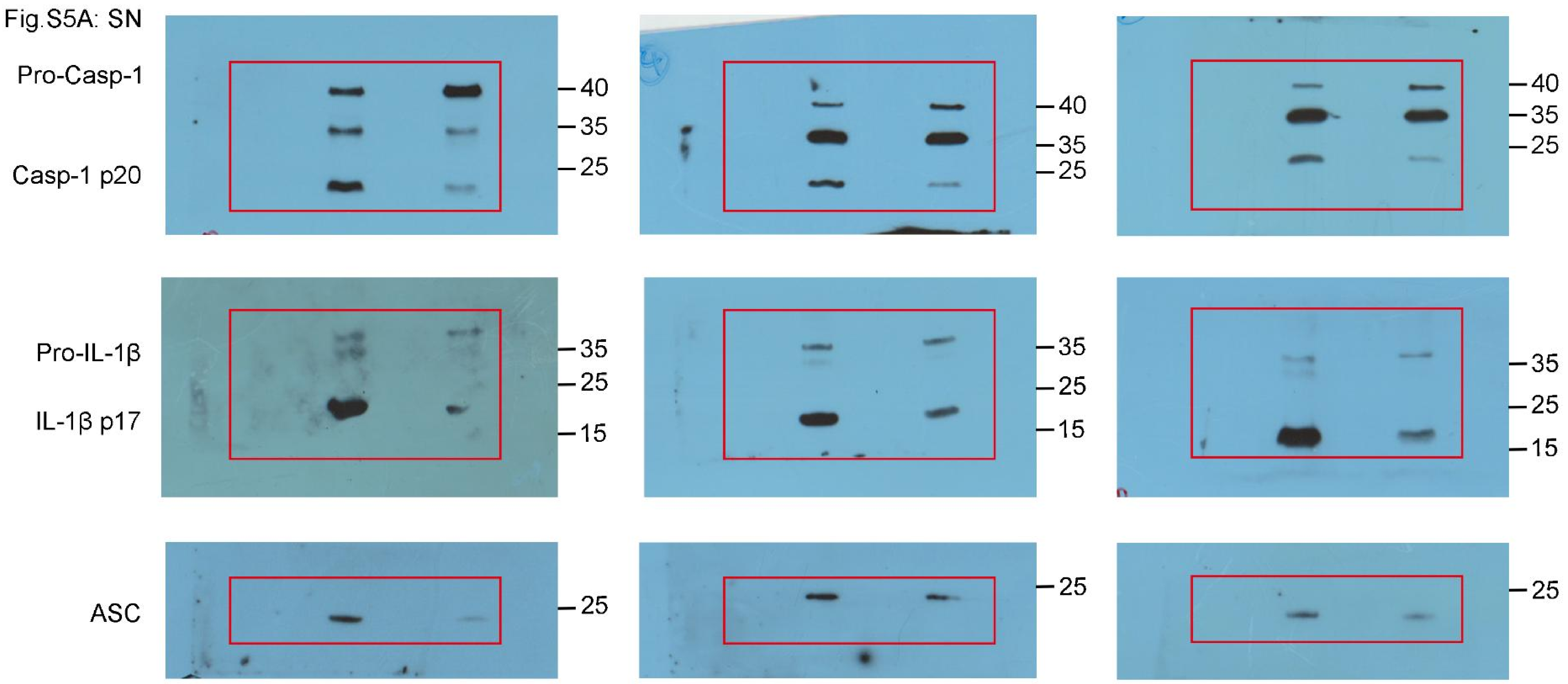
Fig.S5A: SN
Pro-Casp-1
Casp-1 p20
Pro-IL-1β
IL-1β p17
ASC
40
35
25
15

Fig.S5A: Lys

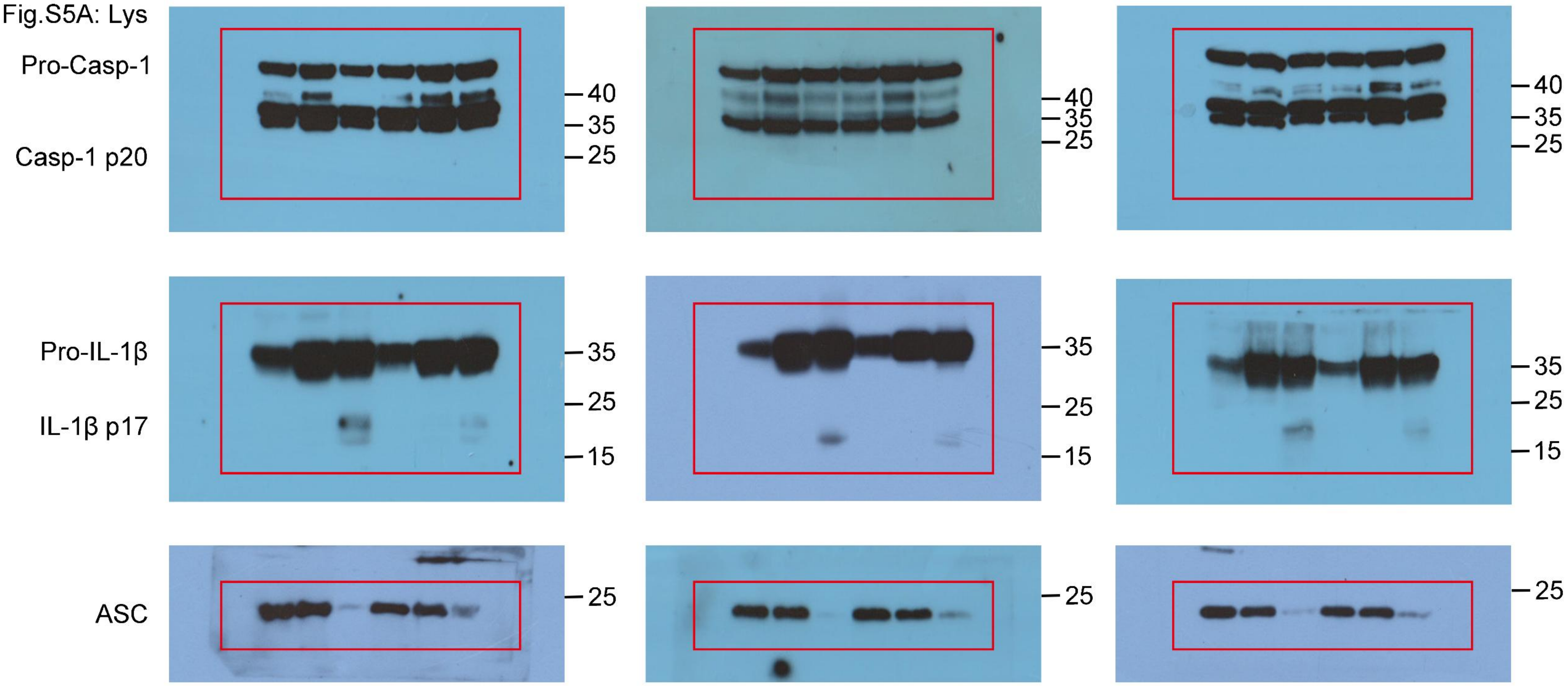

Fig.S5A: Lys

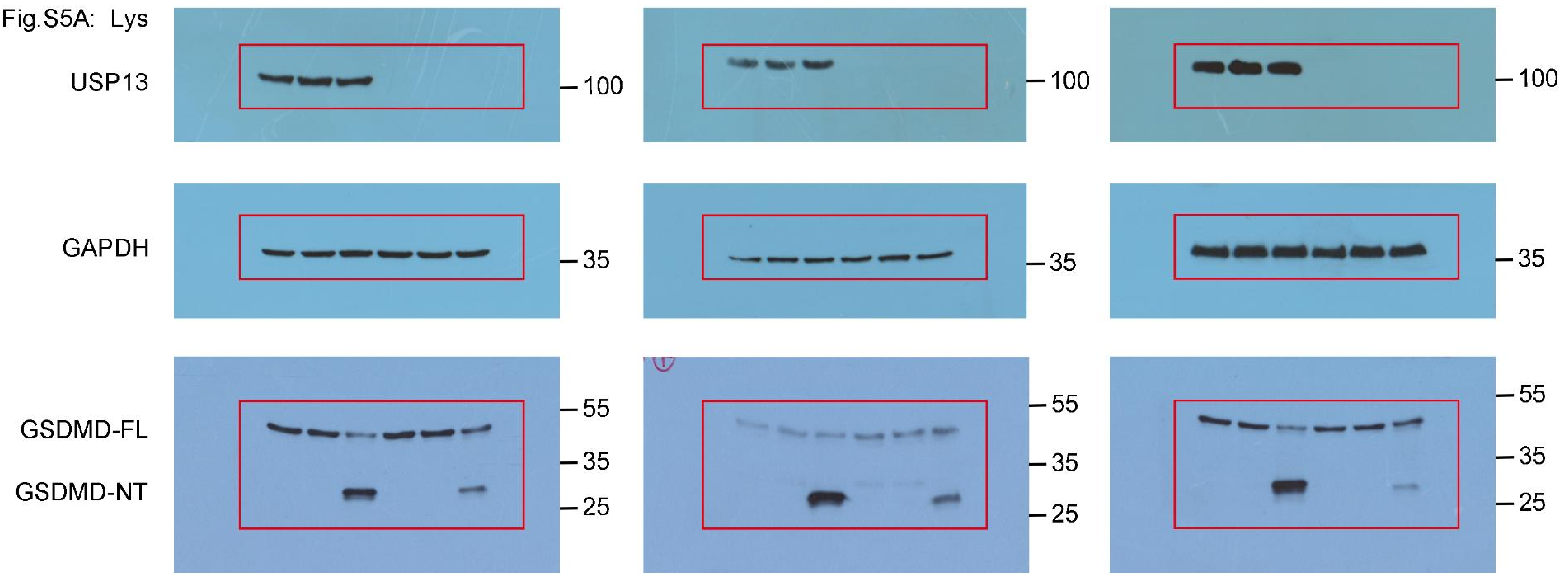

USP13
100
100
100
GAPDH
35
35
35
GSDMD-FL
GSDMD-NT
55
35
25
55
35
25
55
35
25

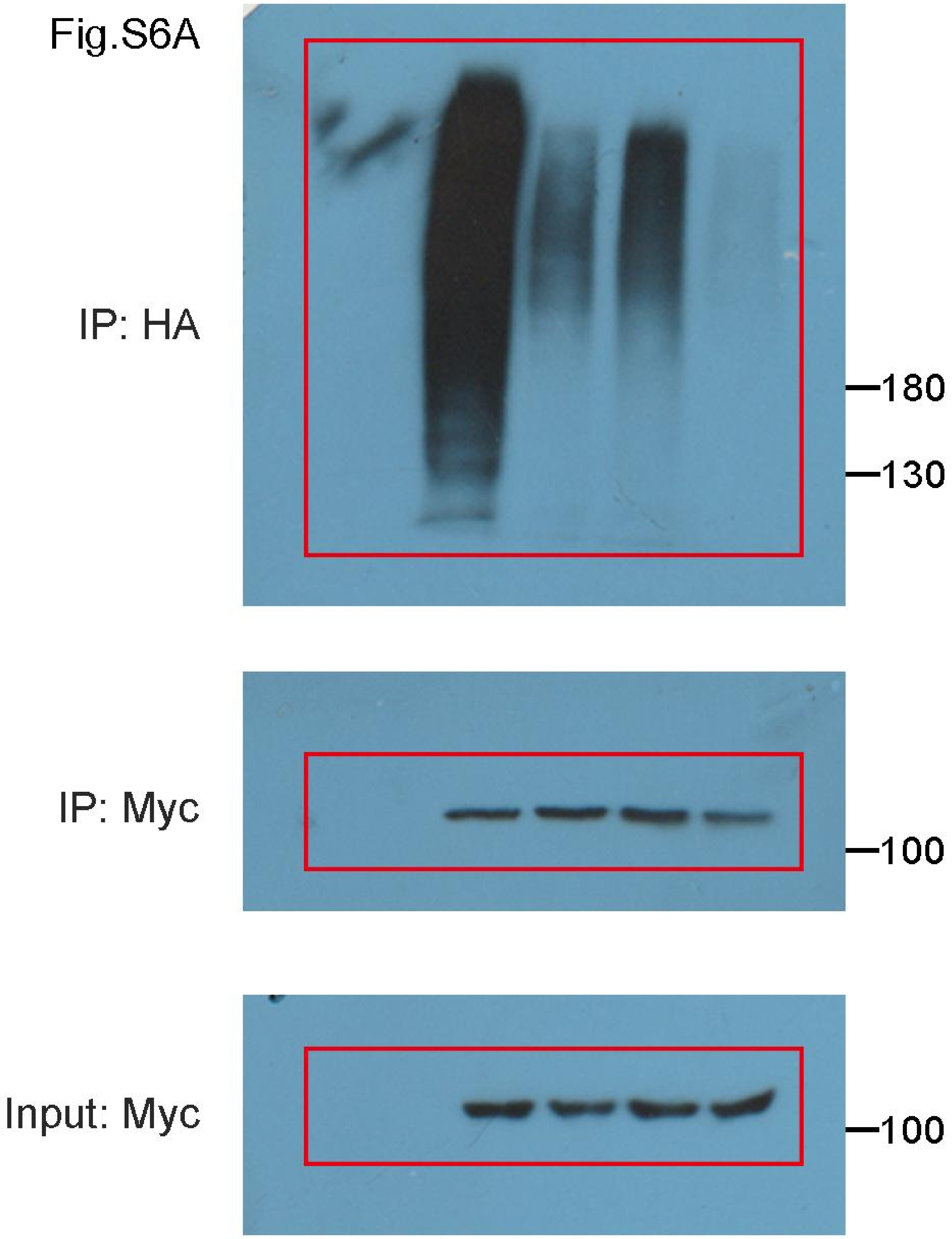
Fig.S6A
IP: HA
180
130
IP: Myc
100
Input: Myc
100

Fig.S6B

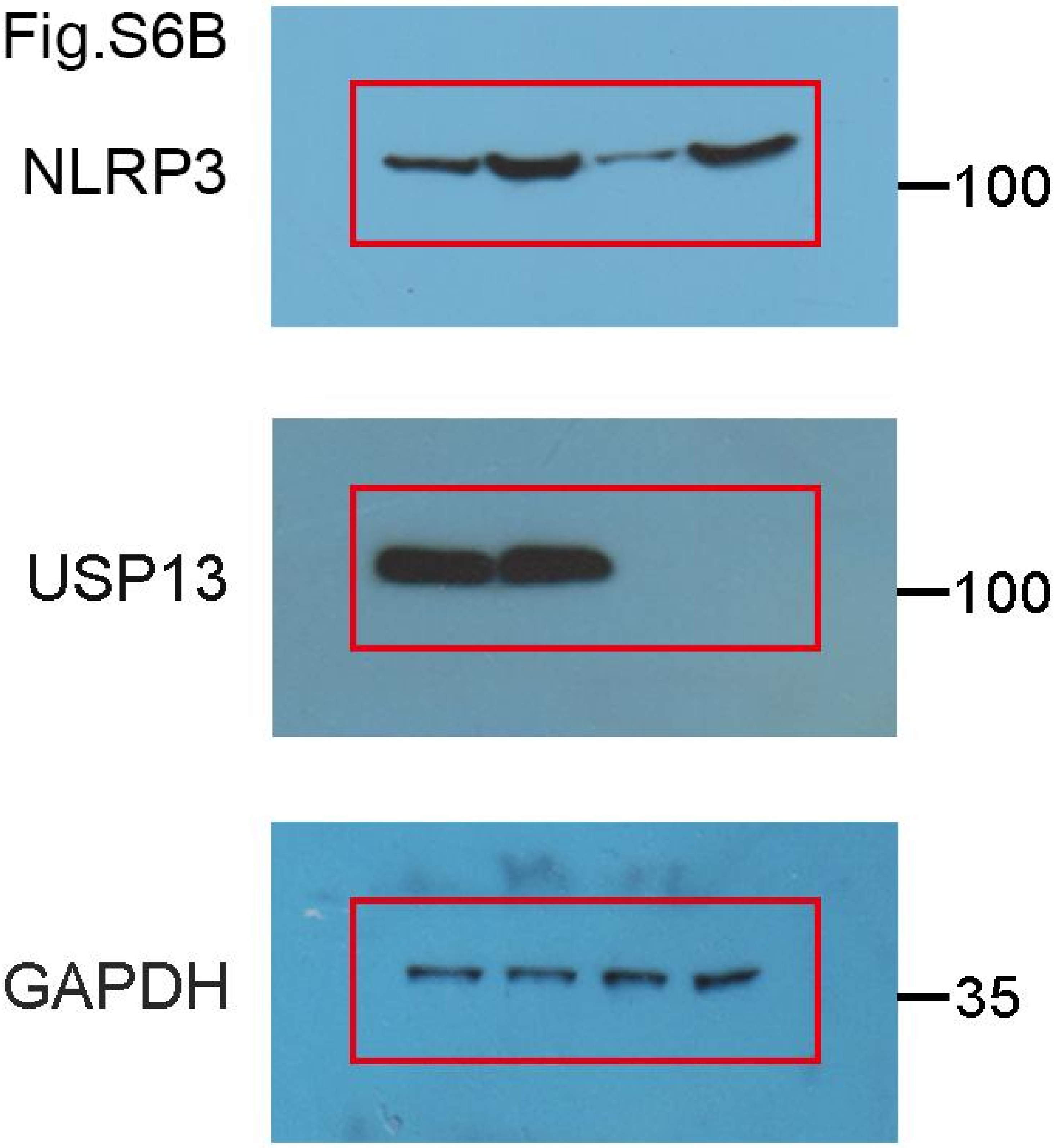

Supplement: Supplementary file 1 — Figs. S1 to S7 Legend for table S1 Legend for data S1 Data S2 [file sciadv.adx3827_sm.pdf]
